# Preconception lifestyle interventions for women—a systematic review and meta-analysis of intervention characteristics and behaviour change techniques

**DOI:** 10.1093/humupd/dmaf021

**Published:** 2025-08-22

**Authors:** Sophia Torkel, Evangeline Mantzioris, Anthony Villani, Nicole J Kellow, Dhruv Bhatnagar, Elaine K Osei-Safo, Margaret McGowan, Nur K Abdul Jafar, Nadia Bogatzke, Simon Alesi, Tuba Astarcioglu, Ben W Mol, Robert J Norman, Stephanie Cowan, Rui Wang, Lisa Moran

**Affiliations:** Monash Centre for Health Research and Implementation, Monash University, Clayton, Australia; Clinical and Health Sciences and Alliance for Research in Exercise, Nutrition and Activity (ARENA), University of South Australia, Adelaide, Australia; School of Health, University of the Sunshine Coast, Sippy Downs, Australia; Department of Nutrition, Dietetics & Food, Monash University, Notting Hill, Australia; Monash Centre for Health Research and Implementation, Monash University, Clayton, Australia; Monash Centre for Health Research and Implementation, Monash University, Clayton, Australia; Monash Centre for Health Research and Implementation, Monash University, Clayton, Australia; Monash Centre for Health Research and Implementation, Monash University, Clayton, Australia; Monash Health, Clayton, Australia; Monash Centre for Health Research and Implementation, Monash University, Clayton, Australia; Monash Centre for Health Research and Implementation, Monash University, Clayton, Australia; Department of Obstetrics and Gynaecology, Monash University, Clayton, Australia; Robinson Research Institute, University of Adelaide, Adelaide, Australia; Monash Centre for Health Research and Implementation, Monash University, Clayton, Australia; NHMRC Clinical Trials Centre, University of Sydney, Sydney, Australia; Monash Centre for Health Research and Implementation, Monash University, Clayton, Australia

**Keywords:** diet, physical activity, preconception care, intervention characteristics, behaviour change techniques

## Abstract

**BACKGROUND:**

The time before conception is an important opportunity to improve maternal lifestyle, and hence improve fertility and health. However, the components of effective preconception lifestyle interventions are unclear.

**OBJECTIVE AND RATIONALE:**

This review aimed to assess the association of intervention characteristics and behaviour change techniques with the effect of lifestyle interventions on fertility, obstetric, foetal, anthropometric, and metabolic outcomes in women planning a pregnancy. Understanding the optimal components of preconception lifestyle interventions is essential to improve success of future interventions.

**SEARCH METHODS:**

We searched Ovid MEDLINE, PsycINFO, Embase, Emcare, Scopus, Cochrane Central Register of Controlled Trials, and CINAHL (6 December 2024). We included randomized controlled trials on women planning a pregnancy which assessed the effect of lifestyle intervention compared to standard minimal care or no intervention on fertility, obstetric, foetal, anthropometric, and metabolic outcomes. We performed random-effects meta-analysis with subgroup analysis based on participant characteristics, intervention characteristics (using the Template for Intervention Description and Replication (TIDieR) framework), and behaviour change techniques (using the Behaviour Change Taxonomy v1). We assessed trustworthiness (using the Trustworthiness in Randomised Controlled Trials (TRACT) checklist), risk of bias (using the Cochrane Risk of Bias 2.0 tool), and certainty of the evidence (using the GRADE approach).

**OUTCOMES:**

Following eligibility screening and trustworthiness assessments, we included 24 studies (n = 7795 women), of which the majority were conducted in high-income countries (79%) and studied women with infertility (67%). Risk of bias was low for seven studies, some concerns for 15 studies and high for two studies. Overall, there was no difference in clinical pregnancy (odds ratio [95% CI]: 1.06 [0.84, 1.35], *I*^2^ = 24.22%) or live birth (odds ratio [95% CI]: 1.17 [0.82, 1.67], *I*^2^ = 48.73%) with lifestyle intervention. Odds of clinical pregnancy were higher for interventions delivered over ≥10 sessions (2.17 [1.21, 3.86] vs 0.88 [0.72, 1.07], *P* = 0.004 for subgroup differences) and with the behaviour change technique *Adding objects to the environment* (e.g. provision of intervention-compliant food and/or exercise equipment) (3.51 [1.70, 7.23] vs 0.90 [0.75, 1.08], *P* < 0.001 for subgroup differences). Lifestyle interventions reduced weight (mean difference [95% CI]: −3.87 kg [−5.76, −1.97], *I*^2^ = 95.03%) and fasting blood glucose (mean difference [95% CI]: −0.15 mM [−0.25, −0.04], *I*^2^ = 0%). Greater weight loss was observed for interventions with a weight loss aim (−4.19 kg [−6.30, −1.92] vs −0.81 kg [−1.48, −0.14], *P* = 0.003 for subgroup differences). Greater weight loss was observed for interventions delivered solely via face-to-face (−6.02 kg [−8.96, −3.07]) compared to those delivered via a combination of face-to-face and technology (−2.21 kg [−3.62, −0.81], *P* = 0.02 for subgroup differences).

**WIDER IMPLICATIONS:**

Effectiveness of preconception lifestyle interventions aiming to enhance fertility may be improved by a structured, intensive approach. Preconception lifestyle interventions reduce weight, particularly face-to-face interventions with a weight loss aim. However, these findings based on subgroup analyses should be interpreted with caution and warrant further investigation due to the exploratory nature of the analysis, limited number of studies included, and potential aggregation bias of study-level subgroup effects. Selection of intervention characteristics for future preconception lifestyle interventions should consider patient preferences and practical considerations.

**REGISTRATION:**

This review was prospectively registered in the Prospective Register of Systematic Reviews (PROSPERO) (CRD42022333066).

## Graphical abstract

**Figure:**
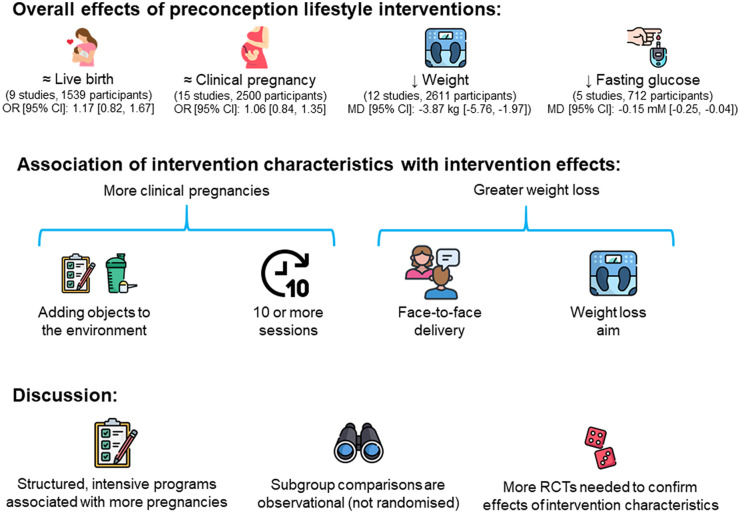
Effectiveness of preconception lifestyle interventions on weight and clinical pregnancy can be enhanced by selection of effective intervention characteristics and behaviour change techniques.

## Introduction

The time prior to conception is an important window of opportunity to optimize maternal health, to reduce the risk of pregnancy complications and improve long-term maternal and offspring health (Fleming *et al.*, 2018; Stephenson *et al.*, 2018). Evidence-based preconception care guidelines, which have been developed in many countries, recommend for screening and management of risk factors affecting pregnancy outcomes, encompassing medical, lifestyle, social, and environmental factors (Johnson *et al.*, 2006; Shawe *et al.*, 2015; Dorney and Black, 2018). Maternal lifestyle recommendations within these guidelines include weight management and optimising physical activity and diet in accordance with population guidelines. However, many women have suboptimal lifestyle behaviours during preconception, including inadequate fruit and vegetable intake, excessive consumption of discretionary foods and inadequate physical activity (Inskip *et al.*, 2009; Chivers *et al.*, 2020; Nkrumah *et al.*, 2020). While these behaviours increase the risk of infertility, pregnancy complications, and adverse neonatal outcomes (Grieger *et al.*, 2018; Oostingh *et al.*, 2019; Purewal *et al.*, 2019), they are modifiable risk factors, highlighting an important opportunity for intervention. However, systematic reviews of preconception lifestyle interventions show no overall effect on live birth and mixed results on the effect on clinical pregnancy (Lan *et al.*, 2017; Boedt *et al.*, 2021b; Caldwell *et al.*, 2024). Further research is therefore needed to characterize the interventions which are effective at promoting fertility and health for preconceptional women.

While existing systematic reviews have assessed some intervention characteristics (duration and medication use) in preconception weight loss interventions (Caldwell *et al.*, 2024), there is a need for further evidence synthesis on the effects of other intervention characteristics in preconception lifestyle interventions, including non-weight-centric interventions. Intervention characteristics can be characterized according to the Template for Intervention Design and Replication (TIDieR) framework across features including materials, procedures, intervention providers, format, setting, and duration, hence allowing for replication and implementation of interventions (Hoffmann *et al.*, 2014). In addition, behaviour change techniques (BCTs) are the active ingredients used to elicit behaviour change in interventions (Michie *et al.*, 2013) and it is important to understand what BCTs should be utilized within preconception lifestyle interventions. Previous systematic reviews have employed subgroup analysis by TIDieR characteristics and BCTs to determine the association of intervention characteristics with the effects of lifestyle interventions in a range of populations, including postpartum women (Lim *et al.*, 2019, 2020), adults at risk of type two diabetes mellitus (Chen *et al.*, 2021), adults of reproductive age (Awoke *et al.*, 2022), and adults above a healthy weight (Samdal *et al.*, 2017). The optimal intervention characteristics identified in these reviews differed according to the populations studied, and there is therefore a need to evaluate the effect of intervention characteristics in lifestyle interventions for preconceptional women.

The aims of this systematic review and meta-analysis were therefore: (i) to assess the effects of preconception lifestyle interventions on fertility, obstetric, foetal, anthropometric, and metabolic outcomes in women planning a pregnancy and (ii) to assess the association of intervention characteristics (as defined by TIDieR and BCTs) with the effect of the intervention.

## Methods

The study protocol for this systematic review was prospectively registered with PROSPERO (CRD42022333066), with the registration amended prior to data analysis to add details on planned analysis. This review is reported in accordance with the Preferred Reporting Items for Systematic Reviews and Meta-analyses (PRISMA) statement (Page *et al.*, 2021).

### Search strategy

Both databases and clinical trials registers were searched to identify potentially relevant studies. For database searches, a comprehensive search strategy was developed, with the use of relevant free text words, and subject headings adapted for different databases. The following databases were searched from inception to 6 December 2024: Ovid MEDLINE(R), Ovid PsycINFO, Ovid Embase, Ovid Emcare, Scopus, and Cochrane Central Register of Controlled Trials. CINAHL Plus was searched from inception to 31 May 2023 and CINAHL Complete was searched from 31 May 2023 to 6 December 2024 due to an upgrade in our access to CINAHL. The search strategy reflected three key concepts: eligible populations (terms related to preconception and infertility), eligible interventions (terms related to lifestyle interventions, defined as diet and/or physical activity), and eligible study designs (using the Cochrane Highly Sensitive Search Strategy for identifying randomized trials). Search terms within the same concept were combined using the ‘or’ Boolean operator, and the three concepts were combined using the ‘and’ Boolean operator. The full search strategies for each database are available in Supplementary Tables S1–S6. For the clinical trial register searches, both clinicaltrials.gov and WHO ICTRP were searched on 4 January 2023. Additional methods of study identification included expert referral and hand-searching of references of included studies and relevant systematic reviews.

### Eligibility criteria

The eligibility criteria were defined using the population, intervention, comparator, outcomes, and study design (PICOS) framework. Details of the eligibility criteria are available in Supplementary Table S7. Eligible participants were non-pregnant women of childbearing age with an intention to conceive. Animal studies and studies which included women with BMI <18 kg m^−2^ or a hereditary disorder in one or both parents were excluded. Eligible interventions were lifestyle modifications aiming to optimize nutritional and/or physical activity status. Trials focusing solely on micronutrient supplementation, alcohol, smoking cessation/reduction, or diabetes control were ineligible. Eligible comparators were no intervention or standard minimal care. Regarding concurrent medication use, studies were only eligible if medication use was consistent between groups (i.e. studies were ineligible if medications were used in the intervention group but not the control group, or vice versa). The primary outcomes were live birth, clinical pregnancy, anthropometric outcomes (defined as those pertaining to the measurement of dimensions of the human body, e.g. body weight), and metabolic outcomes (defined as metabolic parameters that can indicate a participant’s health status, e.g. blood glucose). The secondary outcomes were fertility outcomes (defined as outcomes relating to ability to have offspring, e.g. time to pregnancy), obstetric outcomes (defined as outcomes relating to maternal health before, during, and after parturition, e.g. gestational diabetes), foetal outcomes (defined as outcomes relating to offspring health, e.g. low birth weight), hormonal outcomes (defined as the level of hormones in the body, e.g. testosterone), quality of life, and maternal mortality. Fertility outcomes were applicable to infertility populations only. Eligible study designs were randomized controlled trials. Conference abstracts, clinical trial registrations, and protocols were excluded unless a full-text article reporting on the results was also available. There was no exclusion criterion based on language, and translations were sought for studies published in languages other than English.

### Study selection

For title and abstract screening, all records were screened by two independent reviewers, with all studies screened by S.T. and the second vote on eligibility distributed among five reviewers (A.V., D.B., E.M., E.K.O.-S., and T.A.). Conference abstracts, clinical trial registrations, and protocols of studies which otherwise appeared to meet eligibility criteria were progressed to full-text screening, at which stage we checked if an eligible publication of the study was available. For full-text screening, all English language records were screened by two independent reviewers, with all studies screened by S.T. and the second vote on eligibility distributed among seven reviewers (A.V., D.B., E.M., N.J.K., S.A., S.C., and T.A.). Two reports in full-text screening were published in Persian; for these, M.B. assessed eligibility and provided a partial translation according to the eligibility criteria for verification of eligibility by S.T. Eligibility assessment was performed using Covidence systematic review software (Veritas Health Innovation, Melbourne, Australia. Available at www.covidence.org). Conflicts were resolved by discussion until consensus was achieved.

### Data extraction

For all reports published in English, data were extracted by two independent reviewers in Microsoft Excel, with data extraction performed by one reviewer (S.T.) for all studies, and the second independent extraction distributed among D.B., E.M., M.M., N.K.A.J., and T.A. One included study published one report in Persian; the data from this study were extracted by M.B. The data extracted included: author, year of publication, country, recruitment source, participant demographic information, participant inclusion/exclusion criteria, sample size, attrition rates, intervention characteristics according to the TIDieR framework (reflecting the domains why, what, who provided, how, where, when and how much, tailoring, modifications, and how well) and outcomes. Data for all eligible outcomes were extracted. If outcome data were available for more than one time point, data were extracted for the latest time point in the primary study.

### Coding of behaviour change techniques

BCT Taxonomy Version 1 (BCTTv1) was used to identify BCTs utilized within the lifestyle interventions (Michie *et al.*, 2013); this document provides definitions and examples of each of the 93 BCTs. For each study, the intervention descriptions were reviewed, and the BCTs present were coded. In addition to the main text, the protocol paper and supplementary materials were reviewed where available, for identification of BCTs. BCTs were only coded if the BCT was delivered with the purpose of changing diet, physical activity and/or body weight. Both the intervention and control groups were coded, and only BCTs present in the intervention group but not the control group were included in the analysis. Prior to the commencement of coding, all coders completed the BCTTv1 online training course developed by the authors of BCTTv1 (available via https://www.bct-taxonomy.com/). Each study was independently coded by two reviewers (S.T. for all studies, L.M. for 13 studies, and S.C. for 11 studies). Discrepancies were resolved by discussion among the three reviewers involved in BCT coding (L.M., S.C., and S.T.).

### Risk of bias and trustworthiness assessment

Two reviewers (S.T. for all studies, N.J.K. for 19 studies and D.B. for 1 study and T.A. for 4 studies) independently reviewed the risk of bias in all included studies using version two of the Cochrane Risk of Bias tool (RoB 2.0) (Sterne *et al.*, 2019), with any discrepancies resolved by discussion. RoB 2.0 assesses bias arising from five domains: randomization procedures, deviations from intended interventions, missing outcome data, outcome measurement, and selective reporting of results. Additionally, integrity assessment was performed using the Trustworthiness in RAndomised Controlled Trials (TRACT) checklist (Mol *et al.*, 2023) by two independent reviewers (S.T. for all studies with the second assessment distributed among D.B., E.M., N.B., and T.A.), with any discrepancies in ratings resolved by discussion. The TRACT checklist evaluates seven domains: governance, author group, plausibility of intervention usage, timeframe, drop-out rates, baseline characteristics, and outcomes. Three authors (B.W.M., R.W., and S.T.) discussed the completed checklists to determine which studies required further investigation regarding integrity. For any studies which were deemed as requiring further investigation regarding integrity, corresponding authors were emailed with a request for clarification. If no adequate explanation was provided or no response from the authors, the study was categorized as ‘Awaiting Classification’.

### Synthesis methods

Random-effects meta-analyses using the Restricted Maximum-Likelihood method were performed using Stata 18. A random-effects model was used due to expected variations in treatment effect arising from between-study heterogeneity. Effect estimates were presented as odds ratio (OR) for categorical variables or mean difference (MD) for continuous variables with 95% CI. I^2^ was used to assess the proportion of variability due to heterogeneity. For continuous outcomes that were reported as median and interquartile range, mean, and SD were estimated, when appropriate, in accordance with the Cochrane Handbook (Chapter 7.7.3.5) (Chandler *et al.*, 2019). Unit conversions were calculated as required by S.T. with verification by R.W. If studies reported an SD of 0 for an outcome, the study was excluded from the analysis for this outcome. Where 10 or more studies were included in a meta-analysis, subgroup analyses were performed according to population (women with and without infertility) and intervention characteristics according to the TIDieR (use of a named theoretical framework, health professional delivery, intervention duration, number of sessions, format, use of technology, intervention type, whether weight loss was an aim, continuation of the intervention into pregnancy, tailoring and use of strategies to improve fidelity) and BCTs (any BCT from BCTTv1 which appeared uniquely in the intervention of at least one study reporting the outcome, as well as whether the total number of BCTs uniquely in the intervention was <5 or ≥5). In addition, for primary outcomes, sensitivity analysis restricted to women with obesity (BMI ≥30 kg m^−2^) was performed. For outcomes where 10 or more studies were included in a meta-analysis, a contour-enhanced funnel plot was used to evaluate small-study effect.

### Assessment of certainty of the evidence

We assessed the certainty of the evidence for the key outcomes (live birth, clinical pregnancy, weight, and waist circumference) using the GRADE criteria (Brożek *et al.*, 2009), which assesses the certainty of the evidence as very low, low, moderate, or high. The GRADE criteria include risk of bias, inconsistencies in results across studies, indirectness of the relationship of the evidence to the health care question, imprecision in effect estimates, and publication bias. Certainty assessments were conducted using GRADEpro (available via https://www.gradepro.org/) by S.T. with verification by R.W.

## Results

**Figure 1.**
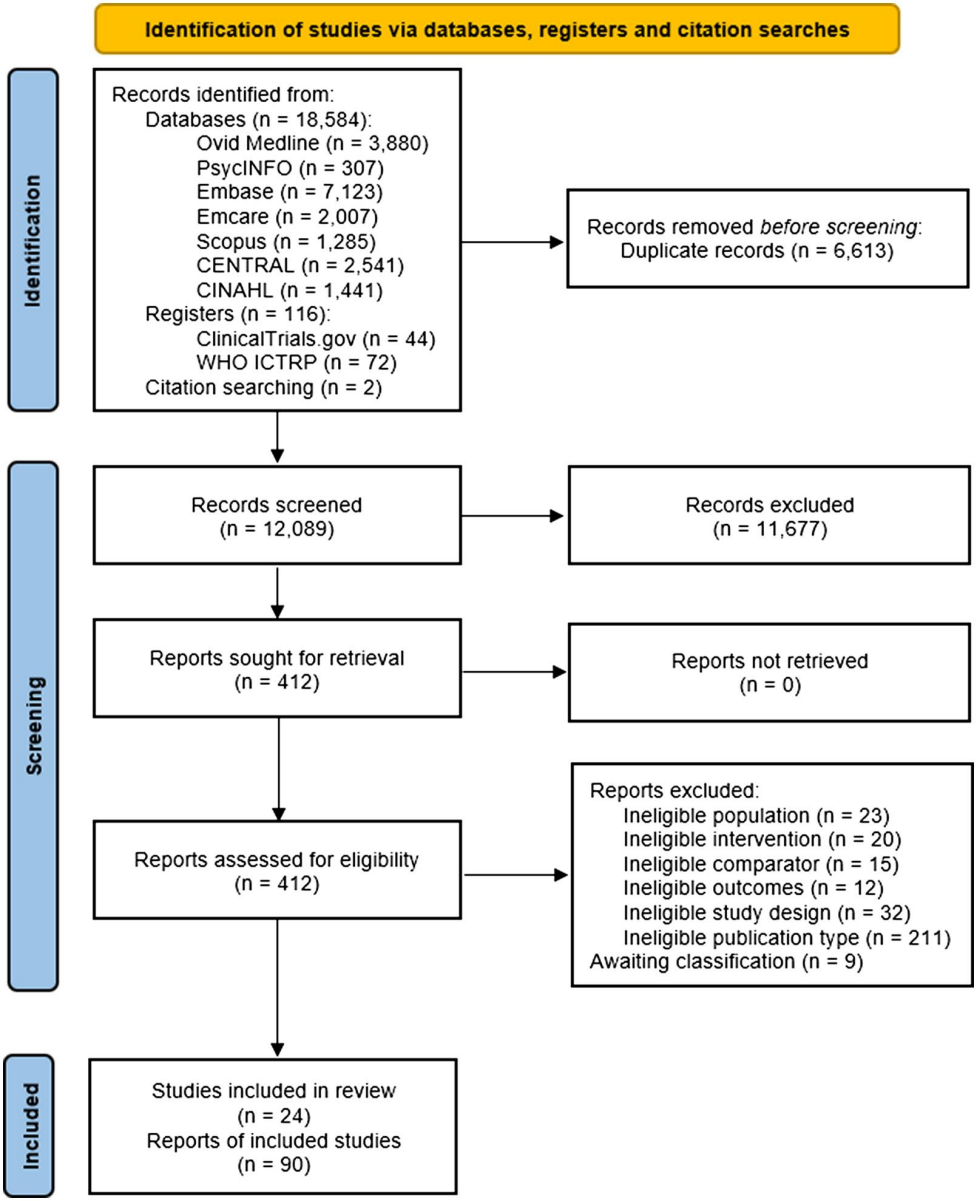
**Flowchart of study selection**.

The study selection process is illustrated in Fig. 1. The database searches returned 18,854 records, the clinical trial register searches returned 116 records and two additional records were identified via citation searching. After the removal of 6,613 duplicates, 12,089 records were screened based on titles and abstracts and 412 records were screened based on full-text. After identifying two additional eligible reports via citation searching, we identified 30 studies (99 reports) which met the eligibility criteria. However, 6 studies (9 reports) were categorized as awaiting classification due to integrity concerns, and we included the remaining 24 studies (90 reports) in our review. Exclusion reasons and citations for studies excluded based on full text or categorized as awaiting classification are available in Supplementary Table S8. Of the included studies, 23 studies published all reports in English. One study published one report in English and one report in Persian, with the English language article reporting on anthropometry data and the Persian language article reporting on quality of life data.

### Characteristics of included studies

The characteristics of the 24 included studies are presented in Table 1. The majority of studies (79%) were conducted in high-income countries, with the most frequently represented countries being The Netherlands (5 studies), Australia (4 studies), and the USA (3 studies). The number of participants randomized ranged from 18 to 1579. The majority of studies (67%) were conducted in women with infertility. Mean age was reported in 20 studies, and ranged from 25.9 years (Koduri *et al.*, 2024) to 33.7 years (Beerendonk *et al.*, 1996). Mean baseline BMI was reported in 16 studies, and ranged from 24.6 kg m^−2^ (Boedt *et al.*, 2023) to 36.4 kg m^−2^ (Sim *et al.*, 2014).

**Table 1.** Characteristics of included studies.

Authors	Country or countries	Sample (n randomized)	Mean age (years)	Mean baseline BMI (kg m^−2^)	**Ethnicity** ^a^	**Intervention (I) and comparator (C)**	Attrition rates
Becker 2015	Brazil	Women with overweight/obesity and female infertility referred for first IVF procedure (n = 35)	31.3	28.8	NR	I: Hypocaloric diet (20 kcal/kg) with low GI and low GL C: Instructed to maintain their usual diet	I: 2/16 (13%) C: 7/19 (37%)
Beerendonk 1996	The Netherlands	Women scheduled for IVF (n = 18)	33.7	NR	Caucasian: 100%	I: Sodium restriction (10–20 mg/day) before IVF C: *Ad libitum* dietary intake before IVF	I: 1/8 (13%) C: 2/10 (20%)
Beerendonk 1999	The Netherlands	Women with medical indication for IVF (n = 119)	33.2	NR	Caucasian: 93% Black: 4% Asian: 3%	I: Sodium restriction (10–20 mmol/day) before IVF C: *Ad libitum* dietary intake before IVF	I: 21/57 (37%) C: 29/62 (47%)
Boedt 2023	Belgium	Women with infertility starting their first IVF/ICSI cycle (n = 211)	30.6	24.6	NR	I: Mobile application with preconception lifestyle coaching and medical treatment information C: Attention control (mobile application with medical treatment information only)	I: 9/106 (8%) C: 8/105 (8%)
Einarsson 2017	Sweden, Denmark, and Iceland	Women with obesity and indications for IVF planning to start first, second, or third IVF treatment (n = 317)	31.6	33.1	Caucasian: 94%	I: Hypocaloric diet (880 kcal/day, aiming to attain BMI as close as possible to normal), followed by weight stabilization and then IVF C: IVF only	I: 8/160 (5%) C: 4/157 (3%)
Espinós 2017	Spain	Women with obesity and primary infertility presenting for first IVF cycle (n = 41)	32.4	34.3	NR	I: Hypocaloric diet (500–800 kcal/day reduction) and aerobic exercise, followed by IVF C: IVF only	I: 0/21 (0%) C: 0/20 (0%)
Hanafiah 2022	Malaysia	Newly married nulliparous women (n = 548)	28.2	26.6	Malay: 88%	I: Encouraged by community health promoter to adopt healthy diet and physical activity C: Standard care (no contact with community health promoter)	I: 127/272 (47%) C: 116/276 (42%)
Hillemeier 2008	USA	Non-pregnant women with no known infertility (n = 692)	26.0	NR	Non-Hispanic white: 91%	I: Guided physical activity and nutrition C: Baseline risk assessment and repeated assessment at 14 weeks	I: 221/473 (47%) C: 109/219 (50%)
Jiskoot 2020	The Netherlands	Women with PCOS, overweight/obesity and a wish to become pregnant (n = 183)	28.7 (median)	32.6	NR	I: Multidisciplinary lifestyle intervention aiming to produce weight loss by aligning diet with the Dutch food guide and aligning exercise with WHO recommendations, with or without SMS C: 5 sessions with short, unstructured consult with treating physician, with risk communication and encouragement to lose weight through publicly available services	I with SMS: 28/60 (47%) I without SMS: 22/63 (35%) C: 26/60 (43%)
Kiel 2018	Norway	Women with overweight/obesity accepted for assisted fertilization (n = 18)	32.3	30.2	NR	I: HIIT (3× per week) C: Regular advice from hospital about physical activity	I: 2/8 (25%) C: 3/10 (30%)
Kiel 2022	Norway and Australia	Women with PCOS undertaking <2 weekly endurance training sessions (n = 64)	29.6	30.5	NR	I: HIIT (3× per week), either high volume or low volume C: Given information about current recommendations for physical activity	I, High volume group: 0/20 (0%) I, Low volume group: 3/21 (14%) C: 2/23 (9%)
Koduri 2024	India	Women with PCOS, infertility, and BMI >23 (n = 60)	25.9	30.6	NR	I: Individualized lifestyle intervention aligned with consensus guidelines for the South Asian region C: One-time referral to dietitian	I: 19/30 (63%) C: 9/30 (30%)
LeBlanc 2021	USA	Non-pregnant women with overweight/obesity and a wish to become pregnant within next 2 years (n = 326)	31.3	34.8	White: 84% Asian: 1.2% Black: 4.2% >1 race: 8.3% Did not report: 2.4%	I: Hypocaloric DASH diet (aiming for weight loss of 0.2–0.4 kg/week) without sodium restriction and graded physical activity targets (working towards 60 min moderate-intensity physical activity/day and working towards 10,000 steps/day) C: Brief 7	I: 2/164 (1%) C: 2/162 (1%)
Lumley 2006	Australia	Interconception women with one previous pregnancy (n = 1579)	NR	NR	NR	I: Address individual lifestyle risk factors C: Home visit from midwife to discuss first pregnancy	I: 205/777 (26%) C: 408/802 (51%)
Mohseni 2021	Iran	Women with PCOS and undergoing infertility treatment and no history of yoga exercises (n = 67)	30.6	25.7	NR	I: Yoga (7× per week) C: Routine care provided by hospital	I: 3/33 (9%) C: 3/34 (9%)
Moran 2011	Australia	Women with overweight/obesity undergoing IVF with GnRH agonist protocols who have previously undergone at least one ART cycle (n = 46)	32.9	33.9	NR	I: Hypocaloric diet (1283 kcal/day) with 1 daily meal replacement and home-based physical activity program (walking and resistance training) C: Standard advice on appropriate diet and lifestyle factors influencing fertility (1 session with no active follow-up)	I: 3/21 (14%) C: 5/25 (20%)
Mutsaerts 2016	The Netherlands	Women with overweight/obesity and infertility seeking assisted reproduction (n = 577)	29.7	36.0 (median)	White: 87%	I: Hypocaloric diet (600 kcal/day reduction, aiming to decrease weight by 5–10%) and structured exercise regimen, followed by fertility treatment C: Fertility treatment only	I: 10/290 (3%) C: 3/287 (1%)
Ng 2021	UK	Women undergoing investigation or treatment for subfertility or recurrent miscarriage (n = 262)	NR	NR	NR	I: Personalized smartphone lifestyle coaching program, to encourage to change unhealthy habits and maintain healthy habits. C: Asked to visit ‘Smarter Pregnancy’ website, provided with standard advice and links to information about periconception health	I: 39/131 (30%) C: 34/131 (26%)
Oostingh 2020	The Netherlands	Women scheduled to start IVF/ICSI treatment within next 3 months (n = 626)	33.0 (median)	23.8 (median)	Dutch: 72% Western: 5% Non-Western: 14% Missing: 9%	I: Personalized smartphone lifestyle coaching program, to encourage to change unhealthy habits and maintain healthy habits. C: Access to online resources and one seasonal recipe per week via email	I: 10/308 (3%) C: 9/318 (3%)
Phelan 2023	USA	Women with overweight/obesity planning pregnancy within next 1–3 years diagnosed with GDM during previous pregnancy (n = 199)	32.6	32.8	American Indian or Alaskan Native: 1.6% Asian: 1.6% Black or African American: 3.2% Native Hawaiian or Pacific Islander: 3.2% White: 42.9% Other: 14.3% ^b^	I: Lifestyle modification program designed to produce 10% weight loss over 16 weeks and weight loss maintenance until conception+standard care with education C: Standard care consisting of general information about preconception health	I: 67/105 (64%) C: 69/94 (73%)
Rono 2018	Finland	Women planning pregnancy within 1 year, and either obesity or previous history of GDM (n = 228)	32.5	29.9	NR	I: Individualized dietary and physical activity counselling, with weight loss of 5–10% before pregnancy recommended for women with BMI ≥25 kg m^−2^ at inclusion C: Information leaflets on healthy diet and exercise similar in accordance with routine antenatal care	I: 51/116 (44%) C: 49/112 (44%)
Sim 2014	Australia	Women with obesity planning to commence IVF, ICSI, or cryostored embryo transfer treatment (n = 49)	32.9	36.4	NR	I: Hypocaloric diet (VLED consisting of 609 kcal/day, followed by mildly hypocaloric diet with a 598 kcal/day deficit) and increase physical activity up to 10 000 steps/day, before fertility treatment C: Advised to see GP for weight loss advice; referral to public weight loss service if BMI ≥ 35 kg m^−2^	I: 1/27 (4%) C: 0/22 (0%)
Van Uytsel 2022	Belgium	Interconception women with excessive GWG according to 2009 National Academy of Medicine guidelines (n = 1450)	31.3	NR. 36% had overweight and 16% had obesity before their first pregnancy.	White European: 88% Other: 10% Missing: 2%	I: Nutrition goal setting aligned with Belgian active food triangle and individualized physical activity goal setting C: No intervention	I: 294/724 (41%) C: 336/726 (46%)
Wang 2023	China	Women with infertility, overweight/obesity, and insulin resistance who are planning to start their first or second IVF/ICSI cycle	30.0	27.8	NR	I: Lifestyle modification program aiming to produce 5–10% weight loss via energy-reduced diet and physical activity before IVF/ICSI C: IVF/ICSI only	I: 4/40 (10%) C: 4/40 (10%)

a Ethnicity reported using the same terminology as the included studies. Where ethnicity data were unavailable, data on race, ancestry, or heritage were used where available.

b Participants could select multiple ethnicities; number of participants with missing ethnicity data not reported.

C, comparator; GDM, gestational diabetes mellitus; GP, general practitioner; GWG, gestational weight gain; I, intervention; NR, not reported; SMS, short message service; VLED, very low energy diet; WHO, World Health Organisation.

### Risk of bias of included studies

**Figure 2.**
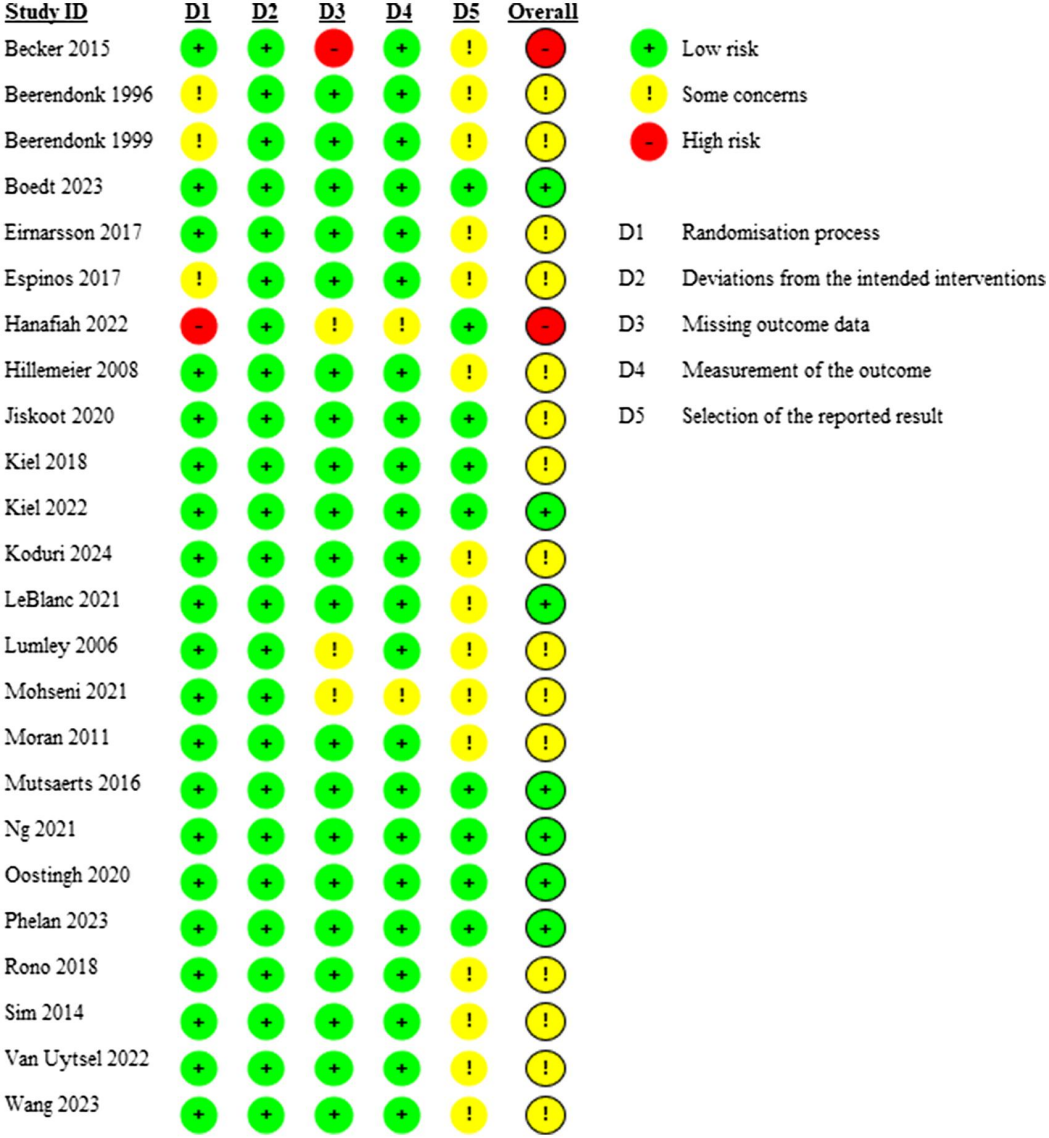
**Risk of bias of included studies. No studies were omitted based on risk of bias**.

The overall risk of bias was low for seven studies, some concerns for 15 studies and high for two studies (Fig. 2). No studies were omitted based on risk of bias. Sources of bias included the randomization process (some concerns for three studies and high risk for one study), missing outcome data (some concerns for three studies and high risk for one study), measurement of the outcome (some concerns for two studies), and selection of the reported result (some concerns for 15 studies).

### Interventions

#### Intervention characteristics according to the TIDieR checklist

The intervention characteristics according to the TIDieR checklist are summarized in Table 2, with detailed information provided in Supplementary Table S9.

**Table 2.** Intervention characteristics according to the template for intervention description and replication.

		Becker 2015	Beerendonk 1996	Beerendonk 1999	Einarsson 2017	Boedt 2023	Espinós 2017	Hanafiah 2022	Hillemeier 2008	Jiskoot 2020	Kiel 2018	Kiel 2022	Koduri 2024	Leblanc 2021	Lumley 2006	Mohseni 2021	Moran 2011	Mutsaerts 2016	Ng 2021	Oostingh 2020	Phelan 2023	Rono 2018	Sim 2014	Van Uytsel 2022	Wang 2023
Why	Named theoretical framework					✓			✓					✓					✓	✓	✓			✓	
What	Diet included	✓	✓	✓	✓	✓	✓	✓	✓	✓			✓	✓	✓		✓	✓	✓	✓	✓	✓	✓	✓	✓
	Physical activity included					✓	✓	✓	✓	✓	✓	✓	✓	✓	✓	✓	✓	✓			✓	✓	✓	✓	✓
	Weight loss component	✓			✓		✓			✓			✓	✓			✓	✓	✓		✓	✓	✓	✓	✓
	Comparator																								
	No lifestyle intervention	✓	✓	✓	✓	✓	✓	✓	✓						✓	✓		✓						✓	✓
	Brief lifestyle intervention									✓	✓	✓	✓	✓			✓		✓	✓	✓	✓	✓		
Who	Health professional delivery	✓	✓	✓	✓	✓	✓	✓		✓			✓		✓		✓	✓				✓	✓		✓
How	Format																								
	Individual	✓	✓	✓	✓	✓	✓	✓	✓	✓	✓	✓	✓	✓	✓	✓	✓	✓	✓	✓	✓	✓		✓	✓
	Group							✓	✓	✓						✓						✓	✓		
	Mode of delivery																								
	Face-to-face	✓	✓	✓	✓		✓	✓	✓	✓	✓	✓	✓	✓	✓	✓	✓	✓			✓	✓	✓	✓	✓
	Technology					✓		✓	✓	✓^a^				✓	✓	✓	✓	✓	✓	✓	✓			✓	✓
When and how much	Duration of intervention																								
	<10 weeks	✓	✓	✓											✓	✓	✓								
	10–25 weeks				✓		✓		✓		✓								✓	✓			✓		✓
	>25 weeks					✓		✓		✓		✓	✓	✓				✓			✓	✓		✓	
	Number of sessions		^b^	^b^	^b^																				
	<10	✓				✓		✓					✓		✓		✓	✓	✓	✓		✓		✓	✓
	≥10						✓		✓	✓	✓	✓		✓		✓					✓		✓		
	Continuation into pregnancy													✓						✓		✓		✓	
Tailoring	Interventions tailored				✓	✓	✓	✓		✓	✓	✓	✓	✓	✓			✓	✓	✓	✓	✓	✓	✓	✓
How well	Strategies to improve fidelity	✓					✓		✓	✓				✓		✓		✓			✓	✓	✓	✓	

a Technology only used for one of the two intervention arms (lifestyle intervention with short message service).

b Insufficient information to determine the number of sessions.

##### Why (theoretical framework)

Seven studies cited at least one theoretical framework to support the intervention, with the most commonly reported being the social cognitive theory (5 studies), the transtheoretical model (3 studies), and Fogg’s behaviour model (2 studies).

##### What (intervention type, materials, and procedures)

Six studies reported on diet-only interventions, 3 studies reported on physical activity-only interventions, and 15 studies reported on combined diet and physical activity interventions. Dietary strategies included sodium restriction, low glycaemic index diets, Dietary Approaches to Stop Hypertension (DASH) diet, aligning dietary intake with national/regional guidelines, and meal replacement programs. Physical activity strategies included yoga, high-intensity interval training, aerobic exercise (e.g. walking or stationary bicycling), and home-based resistance training. Fourteen studies included weight loss as an aim of the intervention, either for all participants (11 studies) or only for those above a healthy weight (3 studies). Twenty-two studies reported on materials used, which included written resources (e.g. menus, textbooks, and information cards), food (e.g. olive oil and dried fruit), meal replacement products, cardiovascular exercise machines (e.g. treadmills, stationary bicycles, and elliptical trainers), self-monitoring tools (e.g. heart rate monitors, pedometers, body weight scales, food diaries, and physical activity diaries), and mobile phone applications. All studies reported on procedures used, which included lifestyle counselling (pertaining to diet, physical activity, weight and/or psychological wellbeing), digital health contact (e.g. communication via email, short message service, or via a mobile phone application), and supervised exercise sessions. The comparator group in the studies received either no lifestyle intervention (13 studies) or brief verbal and/or written lifestyle advice (11 studies).

##### Who (intervention provider)

Fifteen studies reported at least one health professional was involved in delivery of the intervention, most commonly dietitians (11 studies) or nurses/midwives (5 studies).

##### How (use of technology, individual, or groups)

Three studies reported on interventions which used technology only with no face-to-face sessions. Face-to-face sessions only were used in 10 studies, plus one of the two intervention arms of an additional study. A combination of face-to-face and technology was used for 10 studies, plus one of the two intervention arms of an additional study. Technologies used to support interventions included telephone counselling, short message service contact, emails, websites, WhatsApp group chats, mobile phone applications, and video conferencing. Eighteen studies used individual format only, 1 study used group format only, and 5 studies used a combination of individual and group formats.

##### Where (location of intervention)

Seventeen studies reported on the location of the intervention, which included hospitals, community settings (e.g. church buildings, community centres), research facilities, gymnasiums, private clinics, participants’ homes, and online.

##### When and how much

For the 15 studies conducted in women undergoing fertility treatment, the timing of the lifestyle intervention commencement ranged from 6 months before the commencement of fertility treatment to the same time as the commencement of fertility treatment. The number of sessions ranged from 0 to 156. Nine studies reported on the duration of sessions, which ranged from 20 to 150 min. Four studies reported on interventions which continued into pregnancy, either with the same session frequency as the preconception phase of the intervention (2 studies) or with the session frequency fixed at three sessions over the course of the pregnancy (2 studies).

##### Tailoring

Eighteen studies reported on interventions which were tailored. Tailoring was based on individual risk factors, goals, anthropometry (e.g. individualized energy and macronutrient targets based on body weight), and contraindications to particular lifestyle strategies (e.g. exercise substitutions provided for participants with mobility limitations).

##### How well

Eleven studies reported on the use of at least one strategy to improve fidelity. Fidelity strategies included provision of manuals, mentoring, and supervision to intervention providers. Twenty studies reported on the use of at least one method to assess fidelity. Methods for assessing fidelity included self-reported lifestyle behaviours (diet or physical activity), weight measurement, biomarker testing, mobile phone application usage, and session attendance.

#### Behaviour change techniques

The BCTs uniquely present in the intervention group are summarized in Table 3, with examples provided in Supplementary Table S10. Thirty BCTs were identified across all studies. The range of BCTs uniquely present in the intervention group was 0–12. The BCTs most commonly reported uniquely in the intervention group were *Instruction on how to perform the behaviour* (12 studies) (e.g. advising participants on which foods are compliant with the dietary intervention), *Feedback on behaviour* (10 studies) (e.g. emailing participants to provide feedback on their progress with diet and physical activity changes), and *Self-monitoring of behaviour* (10 studies) (e.g. advising participants to track their dietary intake and physical activity).

**Table 3.** Behaviour change techniques uniquely present in the intervention group.

	Becker 2015	Beerendonk 1996	Beerendonk 1999	Boedt 2023	Einarsson 2017	Espinós 2017	Hanafiah 2022	Hillemeier 2008	Jiskoot 2020	Kiel 2018	Kiel 2022	Koduri 2024	Leblanc 2021	Lumley 2006	Mohseni 2021	Moran 2011	Mutsaerts 2016	Ng 2021	Oostingh 2020	Phelan 2023	Rono 2018	Sim 2014	Van Uytsel 2022	Wang 2023
1.1 Goal setting (behaviour)				✓			✓					✓	✓							✓	✓		✓	✓
1.2 Problem solving							✓	✓	✓				✓							✓			✓	
1.3 Goal setting (outcome)							✓	✓	✓				✓				✓			✓			✓	✓
1.4 Action planning																							✓	
1.5 Review behaviour goal(s)				✓									✓				✓				✓			
1.7 Review outcome goal(s)							✓																	
1.8 Behavioural contract																	✓							
2.1 Monitoring of behaviour by others without feedback							✓																	
2.2 Feedback on behaviour				✓			✓	✓	✓								✓	✓	✓			✓	✓	✓
2.3 Self-monitoring of behaviour				✓			✓	✓	✓^a^				✓				✓			✓		✓	✓	✓
2.4 Self-monitoring of outcome(s) of behaviour													✓							✓			✓	✓
2.7 Feedback on outcome(s) of behaviour																						✓		✓
3.1 Social support (unspecified)								✓	✓				✓	✓			✓					✓	✓	
3.3 Social support (emotional)				✓			✓																	
4.1 Instruction on how to perform the behaviour	✓			✓	✓	✓	✓	✓	✓					✓	✓		✓						✓	✓
5.1 Information about health consequences								✓									✓							
5.3 Information about social and environmental consequences																							✓	
6.1 Demonstration of the behaviour								✓		✓	✓				✓									
7.1 Prompts/cues															✓									
8.1 Behavioural practice/rehearsal								✓		✓	✓				✓									
8.2 Behaviour substitution							✓		✓				✓											
8.6 Generalization of target behaviour										✓	✓				✓									
8.7 Graded tasks				✓									✓			✓				✓		✓		
9.1 Credible source	✓			✓	✓	✓	✓										✓					✓		✓
10.8 Incentive (outcome)																	✓							✓
10.10 Reward (outcome)																	✓							✓
11.2 Reduce negative emotions								✓																
12.5 Adding objects to the environment	✓					✓		✓					✓			✓				✓		✓		
12.6 Body changes								✓																
13.2 Framing/reframing									✓											✓				

a Behaviour change technique present in only one of the two intervention arms (lifestyle intervention with short message service).

### Outcomes

Results from meta-analysis are presented in Supplementary Table S11. The summary of findings table (using the GRADE criteria) is presented in Supplementary Table S12.

#### Primary outcomes

##### Live birth

**Figure 3.**
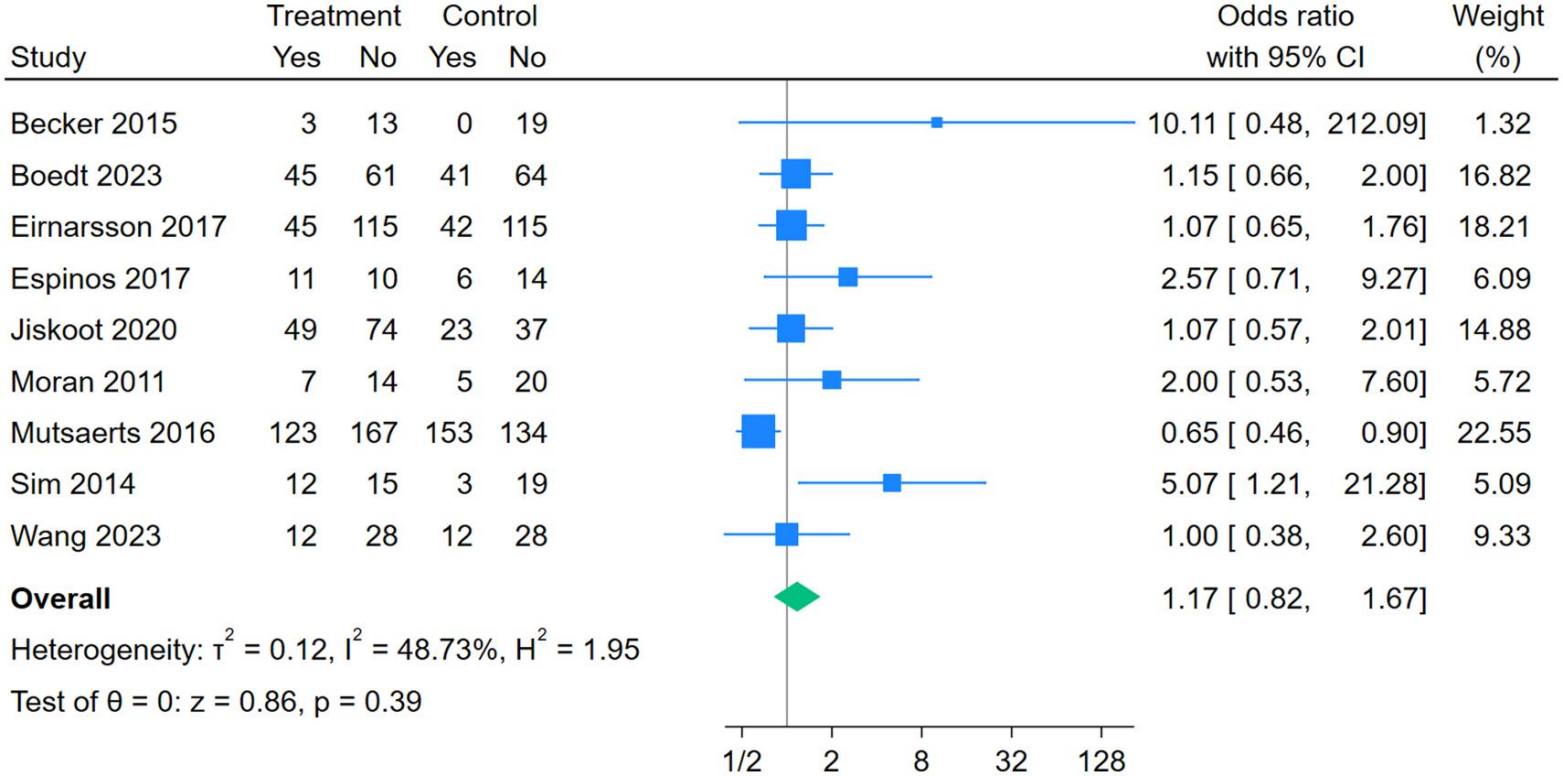
**Random-effects meta-analysis of association between participation in a lifestyle intervention program and live birth.** Odds ratios (95% CI) shown for individual and pooled trials.

Nine studies (Moran *et al.*, 2011; Sim *et al.*, 2014; Becker *et al.*, 2015; Mutsaerts *et al.*, 2016; Einarsson *et al.*, 2017; Espinós *et al.*, 2017; Jiskoot *et al.*, 2020; Boedt *et al.*, 2023; Wang *et al.*, 2023) (1539 women) were included in the meta-analysis for live birth. There was no significant difference in odds of live birth between intervention and control groups (OR [95% CI]: 1.17 [0.82, 1.67], *I*^2^ = 48.73%, moderate certainty evidence) (Fig. 3). If we assume the live birth rate in the control group is 38.8%, we would expect lifestyle intervention results in 42.6% live births (95% CI from 34.2% to 51.4%). No subgroup analysis was performed due to the small number of studies included for this outcome. Sensitivity analysis restricted to studies on women with obesity showed similar findings (3 studies (Sim *et al.*, 2014; Einarsson *et al.*, 2017; Espinós *et al.*, 2017), 397 women, OR [95% CI]: 1.98 [0.77, 5.08], *I*^2^ = 59.30%) (Supplementary Table S13).

##### Clinical pregnancy

**Figure 4.**
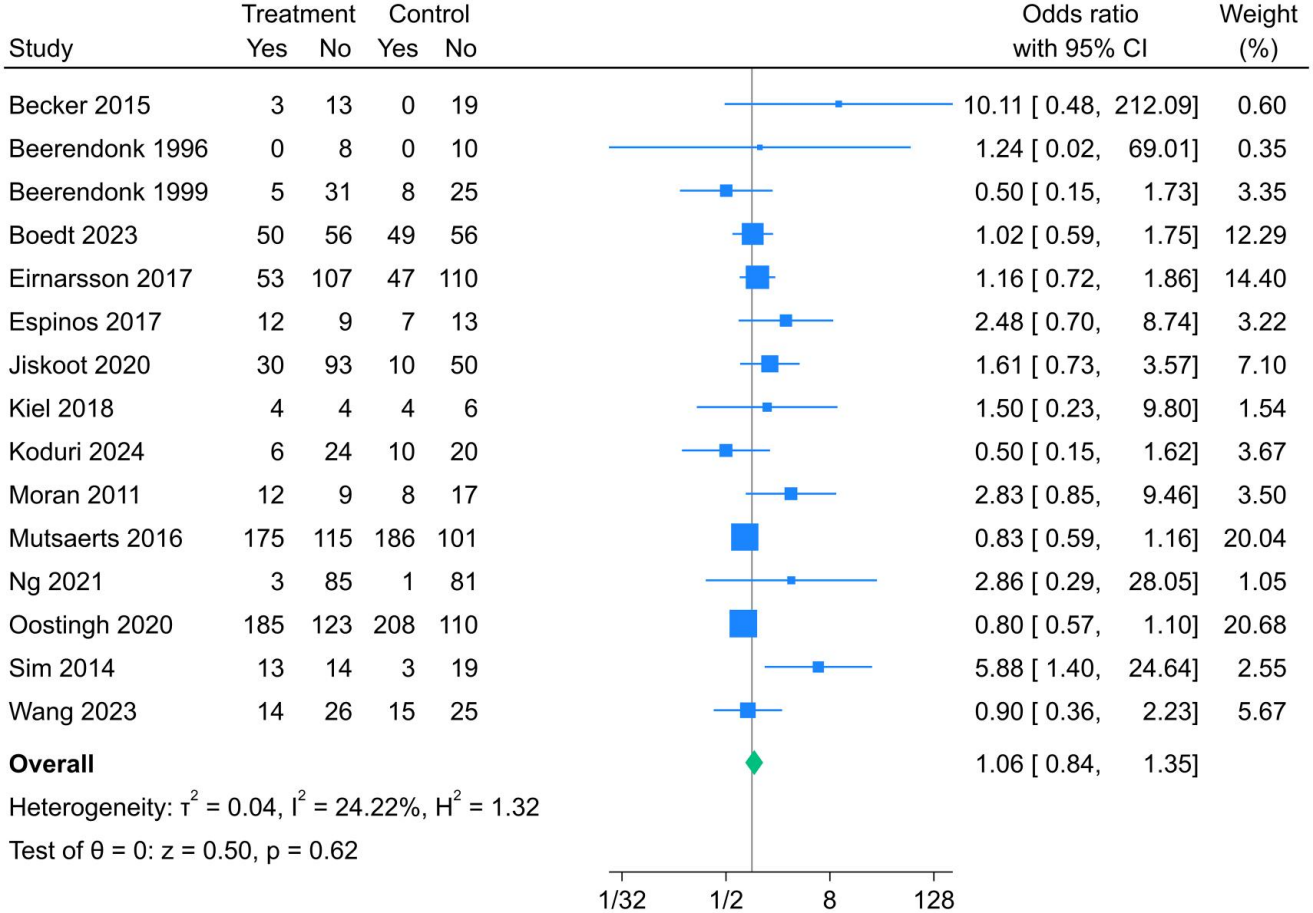
**Random-effects meta-analysis of association between participation in a lifestyle intervention program and clinical pregnancy.** Odds ratios (95% CI) shown for individual and pooled trials.

Fifteen studies (Beerendonk *et al.*, 1996, 1999; Moran *et al.*, 2011; Sim *et al.*, 2014; Becker *et al.*, 2015; Mutsaerts *et al.*, 2016; Einarsson *et al.*, 2017; Espinós *et al.*, 2017; Kiel *et al.*, 2018; Jiskoot *et al.*, 2020; Oostingh *et al.*, 2020; Ng *et al.*, 2021; Boedt *et al.*, 2023; Wang *et al.*, 2023; Koduri *et al.*, 2024) (2500 women) were included in the meta-analysis for clinical pregnancy. There was no significant difference in odds of clinical pregnancy between intervention and control groups (OR [95% CI]: 1.06 [0.84, 1.35], *I*^2^ = 24.22%, moderate certainty evidence) (Fig. 4). If we assume the clinical pregnancy rate in the control group is 45.0%, lifestyle intervention results in 46.6% clinical pregnancies (95% CI from 40.7% to 52.5%). Findings of subgroup analyses are presented in Table 4. Higher odds of clinical pregnancy were observed for group interventions (5.88 [1.40, 24.64]) compared to those delivered in an individual format (0.93 [0.77, 1.11]) or with a combination of individual and group sessions (1.61 [0.73, 3.57]) (*P* = 0.01 for subgroup differences). Higher odds of clinical pregnancy were observed for interventions delivered with 10 or more sessions (2.17 [1.21, 3.86]) compared to those delivered with fewer than 10 sessions (0.88 [0.72, 1.07]). Subgroup differences were not significant for any other TIDieR components. Higher odds of clinical pregnancy were observed with the presence of the BCT *Adding objects to the environment* (e.g. provision of intervention-compliant food and/or exercise equipment) (3.51 [1.70, 7.23] vs 0.90 [0.75, 1.08], *P* < 0.001 for subgroup differences) uniquely in the intervention group, but not the total number of BCTs (≥5 compared to <5) or the presence of any other BCTs.

**Table 4.** Results from subgroup meta-analysis for clinical pregnancy.

Subgroups	Number of studies	Number of participants	Odds ratio [95% CI]	I^2^ (%)	*P*-value for group differences
**Intervention characteristics according to the template for intervention description and replication**					
Named theoretical framework					0.13
* No*	12	1493	1.23 [0.85, 1.77]	38.93	
* Yes*	3	1007	0.86 [0.66, 1.14]	0.00	
Health professional delivery					0.23
* No*	3	814	0.85 [0.58, 1.25]	2.50	
* Yes*	12	1686	1.14 [0.86, 1.51]	25.89	
Duration of intervention					0.34
* <10 weeks*	4	168	1.63 [0.43, 6.19]	48.71	
* 10–25 weeks*	7	1301	1.51 [0.56, 2.82]	65.86	
* >25 weeks*	4	1031	0.91 [0.70, 1.18]	0.00	
Number of sessions^a^					0.004
* <10*	8	1805	0.88 [0.72, 1.07]	0.00	
* ≥10*	4	291	2.17 [1.21, 3.86]	0.00	
Format					0.02
* Individual*	13	2268	0.93 [0.77, 1.11]	0.00	
* Group*	1	49	5.88 [1.40, 24.64]	N/A	
* Both individual and group*	1	183	1.61 [0.73, 3.57]	N/A	
Use of technology					0.29
* Face to face only*	9^b^	700	1.35 [0.78, 2.32]	36.97	
* Technology only*	3	1007	0.86 [0.66, 1.14]	0.00	
* Face to face and technology*	4^b^	793	1.15 [0.67, 1.95]	42.10	
Intervention type					0.51
* Diet only*	6	1235	0.93 [0.67, 1.29]	12.09	
* Physical activity only*	1	18	1.50 [0.23, 9.80]	N/A	
* Diet and physical activity*	8	1247	1.25 [0.83, 1.89]	51.12	
Was weight loss or an energy deficit stated as the aim of the intervention?					0.053
* No*	5	942	0.84 [0.64, 1.10]	0.00	
* Yes*	10	1558	1.37 [0.90, 2.06]	49.66	
Did the intervention continue into pregnancy?					0.08
* No*	14	1874	1.16 [0.88, 1.54]	23.68	
* Yes*	1	626	0.80 [0.57, 1.10]	N/A	
Was the intervention tailored or personalized?					0.48
* No*	4	168	1.63 [0.43, 6.19]	48.71	
* Yes*	11	2332	1.00 [0.81, 1.24]	15.66	
Were any strategies used to improve fidelity?					0.09
* No*	10	1615	0.94 [0.75, 1.18]	2.37	
* Yes*	5	885	1.93 [0.87, 4.28]	67.86	
Comparator type					0.22
* No lifestyle intervention*	8	1348	0.96 [0.77, 1.21]	0.00	
* Brief lifestyle intervention*	7	1152	1.46 [0.78, 2.75]	60.19	
**Behaviour change techniques uniquely present in the intervention group** ^c^					
1.1 Goal setting (behaviour)					0.22
* No*	12	2149	1.30 [0.88, 1.91]	56.00	
* Yes*	3	351	0.90 [0.58, 1.39]	0.00	
1.2 Problem solving					0.25
* No*	14	2317	1.00 [0.80, 1.25]	14.23	
* Yes*	1	183	1.61 [0.73, 3.57]	N/A	
1.3 Goal setting (outcome)					0.38
* No*	12	1660	1.22 [0.83, 1.79]	44.92	
* Yes*	3	840	0.95 [0.65, 1.40]	19.81	
1.5 Review behaviour goal(s)					0.14
* No*	13	1712	1.25 [0.86, 1.82]	41.61	
* Yes*	2	788	0.88 [0.66, 1.17]	0.00	
1.8 Behavioural contract					0.13
* No*	14	1923	1.17 [0.87, 1.57]	29.81	
* Yes*	1	577	0.83 [0.59, 1.16]	N/A	
2.2 Feedback on behaviour					0.29
* No*	8	604	1.24 [0.75, 2.05]	23.79	
* Yes*	7	1896	0.92 [0.75, 1.12]	0.00	
2.3 Self-monitoring of behaviour					0.97
* No*	11^d^	1493	1.10 [0.77, 1.58]	25.99	
* Yes*	5^d^	1007	1.11 [0.74, 1.69]	40.79	
2.4 Self-monitoring of outcome(s) of behaviour					0.66
* No*	14	2420	1.11 [0.84, 1.46]	35.01	
* Yes*	1	80	0.90 [0.36, 2.23]	N/A	
2.7 Feedback on outcome(s) of behaviour					0.42
* No*	13	2371	0.99 [0.80, 1.22]	11.09	
* Yes*	2	129	2.11 [0.34, 13.22]	78.75	
3.1 Social support (unspecified)					0.35
* No*	12	1691	1.00 [0.78, 1.28]	8.78	
* Yes*	3	809	1.65 [0.60, 4.52]	80.19	
3.3 Social support (emotional)					0.75
* No*	14	2289	1.13 [0.83, 1.53]	39.75	
* Yes*	1	211	1.02 [0.59, 1.75]	N/A	
4.1 Instruction on how to perform the behaviour					0.64
* No*	8	1056	1.25 [0.63, 2.49]	57.10	
* Yes*	7	1444	1.05 [0.82, 1.34]	9.73	
5.1 Information about health consequences					0.13
* No*	14	1923	1.17 [0.87, 1.57]	29.81	
* Yes*	1	577	0.83 [0.59, 1.16]	N/A	
6.1 Demonstration of the behaviour					0.72
* No*	14	2482	1.06 [0.83, 1.35]	25.65	
* Yes*	1	18	1.50 [0.23, 9.80]	N/A	
8.1 Behavioural practice/rehearsal					0.72
* No*	14	2482	1.06 [0.83, 1.35]	25.65	
* Yes*	1	18	1.50 [0.23, 9.80]	N/A	
8.2 Behaviour substitution					0.25
* No*	14	2317	1.00 [0.80, 1.25]	14.23	
* Yes*	1	183	1.61 [0.73, 3.57]	N/A	
8.6 Generalization of the target behaviour					0.72
* No*	14	2482	1.06 [0.83, 1.35]	25.65	
* Yes*	1	18	1.50 [0.23, 9.80]	N/A	
8.7 Graded tasks					0.11
* No*	12	2194	0.92 [0.76, 1.11]	0.00	
* Yes*	3	306	2.18 [0.77, 6.19]	67.36	
9.1 Credible source					0.77
* No*	8	1190	1.04 [0.65, 1.65]	32.19	
* Yes*	7	1310	1.13 [0.82, 1.54]	28.04	
10.8 Incentive (outcome)					0.10
* No*	13	1843	1.23 [0.88, 1.74]	39.87	
* Yes*	2	657	0.83 [0.61, 1.15]	0.00	
10.10 Reward (outcome)					0.10
* No*	113	1843	1.23 [0.88, 1.74]	39.87	
* Yes*	2	657	0.83 [0.61, 1.15]	0.00	
12.5 Adding objects to the environment					<0.001
* No*	11	2329	0.90 [0.75, 1.08]	0.00	
* Yes*	4	171	3.51 [1.70, 7.23]	0.00	
13.2 Framing/reframing					0.25
* No*	14	2317	1.00 [0.80, 1.25]	14.23	
* Yes*	1	183	1.61 [0.73, 3.57]	N/A	
Number of behaviour change techniques					0.93
* <5*	10	1576	1.09 [0.74, 1.62]	30.67	
* ≥5*	5	924	1.12 [0.76, 1.66]	41.70	

N/A, not applicable.

a Three studies were excluded from subgroup analysis of the number of sessions due to insufficient information to determine the number of sessions.

b One study (Jiskoot *et al.*, 2020) utilized technology for one of the two intervention arms.

c The following behaviour change techniques were not uniquely present in the intervention of any studies which reported clinical pregnancy as an outcome: 1.4 Action planning, 1.7 Review outcome goal(s), 2.1 Monitoring of behaviour by others without feedback, 5.3 Information about social and environmental consequences, 7.1 Prompts/cues, 11.2 Reduce negative emotions, and 12.6 Body changes.

d One study (Jiskoot *et al.*, 2020) utilized the behaviour change technique 2.3 Self-monitoring of behaviour for one of the two intervention arms.

In sensitivity analysis restricted to studies on women with obesity, the finding was similar to that of the main analysis (3 studies (Sim *et al.*, 2014; Einarsson *et al.*, 2017; Espinós *et al.*, 2017), 397 women, OR [95% CI]: 2.12 [0.82, 5.45], *I*^2^ = 60.70%) (Supplementary Table S13). Funnel plot asymmetry indicated potential small study effects for clinical pregnancy (Supplementary Fig. S1).

##### Anthropometric outcomes

**Figure 5.**
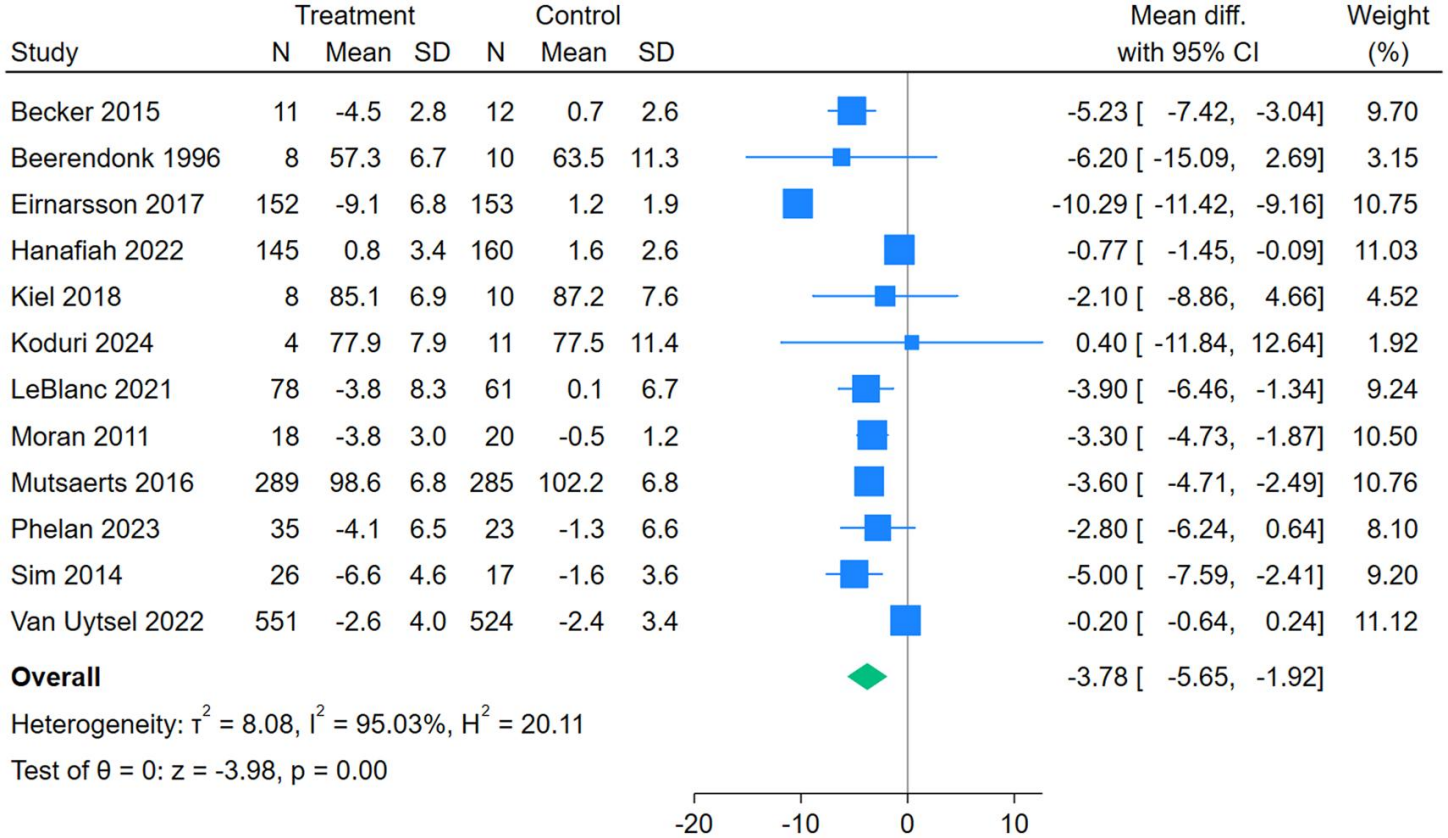
**Random-effects meta-analysis of association between participation in a lifestyle intervention program and weight.** Odds ratios (95% CI) shown for individual and pooled trials.

Twelve studies (Beerendonk *et al.*, 1996; Moran *et al.*, 2011; Sim *et al.*, 2014; Becker *et al.*, 2015; Mutsaerts *et al.*, 2016; Einarsson *et al.*, 2017; Kiel *et al.*, 2018; Leblanc *et al.*, 2021; Hanafiah *et al.*, 2022; Van Uytsel *et al.*, 2022; Phelan *et al.*, 2023; Koduri *et al.*, 2024) (2611 women) were included in the meta-analysis for weight. There were greater reductions in weight for the intervention compared to the control group (MD [95% CI]: −3.78 kg, [−5.65, −1.92], *I*^2^ = 95.03%, low certainty evidence) (Fig. 5). Findings of subgroup analyses are presented in Table 5. Women with infertility lost more weight with lifestyle intervention compared to women without infertility (−5.14 kg [−7.40, −2.87] vs −1.42 kg [−3.02, 0.17], *P* = 0.01 for subgroup differences). Weight loss was significantly different for format (Individual: −4.05 kg [−6.20, −1.91]; Group: −5.00 kg [−7.59, −2.41]; Both individual and group: −0.77 kg [−1.45, −0.09]; *P* < 0.001 for subgroup differences). Greater weight loss was observed for interventions delivered solely via face-to-face (−6.02 kg [−8.96, −3.07]) compared to those delivered via a combination of face-to-face and technology (−2.21 kg [−3.62, −0.81]) (*P* = 0.02 for subgroup differences). Greater weight loss was observed for interventions which stated weight loss as an aim of the intervention (−4.19 kg [−6.30, −1.92]) compared to those which did not (−0.81 kg [−1.48, −0.14]) (*P* = 0.003 for subgroup differences). Subgroup differences were not significant for any other TIDieR components. Less weight loss was observed with the presence of the BCTs *Goal setting (behaviour)*, *Problem solving*, *Goal setting (outcome)*, *Action planning*, *Review outcome goal(s)*, *Monitoring of behaviour by others without feedback*, *Social support (emotional)*, and *Information about social and environmental consequences* uniquely in the intervention group but not the total number of BCTs (<5 compared to ≥5) or the presence of any other BCTs.

**Table 5.** Results from subgroup meta-analysis for weight.

Subgroups	Number of studies	Number of participants	Mean difference [95% CI]	I^2^ (%)	*P*-value for group differences
**Population characteristics**					
Infertility					0.01
* Women without infertility*	4	1577	−1.42 [−3.02, 0.17]	89.03	
* Women with infertility*	8	1034	−5.14 [−7.40, −2.87]	89.33	
**Intervention characteristics according to the template for intervention description and replication**					
Named theoretical framework					0.15
* No*	9	1339	−4.43 [−6.71, −2.15]	95.03	
* Yes*	3	1272	−1.97 [−4.44, 0.51]	75.41	
Health professional delivery					0.11
* No*	4	1290	−1.93 [−4.14, 0.28]	65.10	
* Yes*	8	1321	−4.59 [−7.01, −2.17]	95.03	
Duration of intervention					0.07
* <10 weeks*	3	79	−4.15 [−5.92, −2.38]	34.44	
* 10–25 weeks*	3	366	−6.51 [−11.17, −1.86]	88.16	
* >25 weeks*	6	2166	−1.96 [−3.53, −0.38]	95.03	
Number of sessions^a^					0.19
* <10*	6	2030	−2.40 [−4.16, −0.65]	93.62	
* ≥10*	4	258	−3.98 [−5.54, −2.41]	0.00	
Individual or group format?					<0.001
* Individual*	10	2263	−4.05 [−6.20, −1.91]	93.78	
* Group*	1	43	−5.00 [−7.59, −2.41]	N/A	
* Both individual and group*	1	305	−0.77 [−1.45, −0.09]	N/A	
Use of technology					0.02
* Face to face only*	6	422	−6.02 [−8.96, −3.07]	78.64	
* Face to face and technology*	6	2189	−2.21 [−3.62, −0.81]	90.52	
Intervention type					0.05
* Diet only*	3	346	−7.65 [−11.58, −3.72]	85.26	
* Physical activity only*	1	18	−2.10 [−8.86, 4.66]	N/A	
* Diet and physical activity*	8	2204	−2.51 [−3.89, −1.13]	88.88	
Was weight loss or an energy deficit stated as the aim of the intervention?					0.003
* No*	3	341	−0.81 [−1.48, −0.14]	0.00	
* Yes*	9	2270	−4.19 [−6.30, −1.92]	95.03	
Did the intervention continue into pregnancy?					0.25
* No*	10	1397	−4.27 [−6.31, −2.19]	95.03	
* Yes*	2	1214	−1.83 [−5.43, 1.77]	87.17	
Was the intervention tailored or personalized?					0.69
* No*	3	79	−4.15 [−5.92, −2.38]	34.44	
* Yes*	9	2532	−3.54 [−5.92, −1.17]	96.68	
Were any strategies used to improve fidelity?					0.66
* No*	6	699	−4.22 [−7.95, −0.48]	95.70	
* Yes*	6	1912	−3.28 [−5.002, −1.56]	86.44	
Comparator					0.74
* No lifestyle intervention*	6		−4.16 [−7.44, −0.87]	98.46	
* Brief lifestyle intervention*	6		−3.58 [−4.63, −2.53]		
**Behaviour change techniques uniquely present in the intervention group** ^b^					
1.1 Goal setting (behaviour)					0.005
* No*	7	1019	−5.30 [−7.61, −3.00]	89.33	
* Yes*	5	1592	−1.38 [−2.91, 0.16]	85.07	
1.2 Problem solving					0.01
* No*	8	1034	−5.14 [−7.40, −2.87]	87.75	
* Yes*	4	1577	−1.42 [−3.02, 0.17]	89.03	
1.3 Goal setting (outcome)					0.03
* No*	7	460	−5.44 [−8.06, −2.83]	85.03	
* Yes*	5	2151	−2.00 [−3.61, −0.39]	91.95	
1.4 Action planning					<0.001
* No*	11	1536	−4.24 [−6.13, −2.36]	90.87	
* Yes*	1	1075	−0.20 [−0.64, 0.24]	N/A	
1.5 Review behaviour goal(s)					0.84
* No*	10	1898	−3.79 [−6.08, −1.49]	96.01	
* Yes*	2	713	−3.65 [−4.66, −2.63]	0.00	
1.7 Review outcome goal(s)					0.001
* No*	11	2306	−4.16 [−6.10, −2.22]	92.76	
* Yes*	1	305	−0.77 [−1.45, −0.09]	N/A	
1.8 Behavioural contract					0.87
* No*	11	2037	−3.80 [−5.87, −1.73]	95.24	
* Yes*	1	574	−3.60 [−4.71, −2.49]	N/A	
2.1 Monitoring of behaviour by others without feedback					0.001
* No*	11	2306	−4.16 [−6.10, −2.22]	92.76	
* Yes*	1	305	−0.77 [−1.45, −0.09]	N/A	
2.2 Feedback on behaviour					0.10
* No*	8	614	−4.90 [−7.32, −2.48]	84.19	
* Yes*	4	1997	−2.18 [−4.31, −0.05]	96.03	
2.3 Self-monitoring of behaviour					0.10
* No*	6	417	−5.44 [−8.62, −2.26]	87.79	
* Yes*	6	2194	−2.45 [−4.07, −0.83]	92.11	
2.4 Self-monitoring of outcome (s) of behaviour					0.15
* No*	9	1339	−4.43 [−6.71, −2.15]	93.34	
* Yes*	3	1272	−1.97 [−4.44, 0.51]	75.41	
2.7 Feedback on outcome(s) of behaviour					0.64
* No*	11	2611	−3.66 [−5.69, −1.63]	95.70	
* Yes*	1	43	−5.00 [−7.59, −2.41]	N/A	
3.1 Social support (unspecified)					0.52
* No*	8	780	−4.23 [−6.94, −1.52]	94.19	
* Yes*	4	1831	−2.95 [−5.14, −0.76]	91.05	
3.3 Social support (emotional)					0.001
* No*	11	2306	−4.16 [−6.10, −2.22]	92.76	
* Yes*	1	305	−0.77 [−1.45, −0.09]	N/A	
4.1 Instruction on how to perform the behaviour					0.85
* No*	7	329	−3.61 [−4.66, −2.57]	0.00	
* Yes*	5	2282	−3.98 [−7.58, −0.39]	98.87	
5.1 Information about health consequences					0.87
* No*	11	2037	−3.80 [−5.87, −1.73]	95.24	
* Yes*	1	574	−3.60 [−4.71, −2.49]	N/A	
5.3 Information about social and environmental consequences					<0.001
* No*	11	1536	−4.24 [−6.13, −2.36]	90.87	
* Yes*	1	1075	−0.20 [−0.64, 0.24]	N/A	
6.1 Demonstration of the behaviour					0.62
* No*	11	2593	−3.87 [−5.81, −1.92]	95.63	
* Yes*	1	18	−2.10 [−8.86, 4.66]	N/A	
8.1 Behavioural practice/rehearsal					0.62
* No*	11	2593	−3.87 [−5.81, −1.92]	95.63	
* Yes*	1	18	−2.10 [−8.86, 4.66]	N/A	
8.2 Behaviour substitution					0.27
* No*	10	2167	−4.18 [−6.33, −2.03]	93.80	
* Yes*	2	444	−2.08 [−5.11, 0.95]	81.38	
8.6 Generalization of the target behaviour					0.62
* No*	11	2593	−3.87 [−5.81, −1.92]	95.63	
* Yes*	1	18	−2.10 [−8.86, 4.66]	N/A	
8.7 Graded tasks					0.93
* No*	8	2333	−3.77 [−6.65, −0.90]	97.55	
* Yes*	4	278	−3.64 [−4.71, −2.58]	0.00	
9.1 Credible source					0.15
* No*	7	1361	−2.35 [−4.08, −0.62]	68.80	
* Yes*	5	1250	−4.96 [−8.11, −1.81]	96.90	
10.8 Incentive (outcome)					0.87
* No*	11	2037	−3.80 [−5.87, −1.73]	95.24	
* Yes*	1	574	−3.60 [−4.71, −2.49]	N/A	
10.10 Reward (outcome)					0.87
* No*	11	2037	−3.80 [−5.87, −1.73]	95.24	
* Yes*	1	574	−3.60 [−4.71, −2.49]	N/A	
12.5 Adding objects to the environment					0.80
* No*	7	2310	−3.50 [−6.83, −0.17]	98.09	
* Yes*	5	301	−3.96 [−4.93, −2.98]	1.52	
13.2 Framing/reframing					0.60
* No*	11	2553	−3.87 [−5.88, −1.86]	95.73	
* Yes*	1	58	−2.80 [−6.24, 0.64]	N/A	
Number of behaviour change techniques					0.10
* <5*	6	417	−5.44 [−8.62, −2.26]	95.03	
* ≥5*	6	2194	−2.45 [−4.07, −0.83]	92.11	

N/A, not applicable.

a Two studies were excluded from subgroup analysis of the number of sessions due to insufficient information to determine the number of sessions.

b The following behaviour change techniques were not uniquely present in the intervention of any studies which reported weight as an outcome: 7.1 Prompts/cues, 11.2 Reduce negative emotions, and 12.6 Body changes.

Sensitivity analysis on studies restricted to women with obesity showed similar findings to the main analysis (2 studies (Sim *et al.*, 2014; Einarsson *et al.*, 2017), 348 women) (MD [95% CI]: −7.78 [−12.96, −2.60], *I*^2^ = 92.58%) (Supplementary Table S13). Funnel plot asymmetry indicated potential small study effects for weight (Supplementary Fig. S2).

Lifestyle interventions decreased BMI (9 studies (Moran *et al.*, 2011; Sim *et al.*, 2014; Becker *et al.*, 2015; Mutsaerts *et al.*, 2016; Einarsson *et al.*, 2017; Kiel *et al.*, 2018; Leblanc *et al.*, 2021; Mohseni *et al.*, 2021; Hanafiah *et al.*, 2022), 1506 women, MD [95% CI]: −1.45 kg m^−2^ [−2.23, −0.67], *I*^2^ = 94.17%), waist circumference (9 studies (Moran *et al.*, 2011; Sim *et al.*, 2014; Mutsaerts *et al.*, 2016; Kiel *et al.*, 2018; Mohseni *et al.*, 2021; Hanafiah *et al.*, 2022; Van Uytsel *et al.*, 2022; Wang *et al.*, 2023; Koduri *et al.*, 2024), 2224 women, MD [95% CI]: −2.37 cm [−4.25, −0.49], *I*^2^ = 85.54%, moderate certainty evidence), and hip circumference (5 studies (Becker *et al.*, 2015; Mutsaerts *et al.*, 2016; Mohseni *et al.*, 2021; Wang *et al.*, 2023; Koduri *et al.*, 2024), 745 women, MD [95% CI]: −2.58 cm [−4.54, −0.62], *I*^2^ = 66.93%). Lifestyle interventions increased the odds of excessive gestational weight gain (2 studies (Leblanc *et al.*, 2021; Phelan *et al.*, 2023), 525 women, OR [95% CI]: 1.67 [1.07, 2.59], *I*^2^ = 0.00%). There was no significant difference between intervention and control group in waist to hip ratio, body fat percentage, gestational weight gain (continuous), body fat percentage, fat free mass, or visceral fat. Similar results to our main analysis were found in studies restricted to women with obesity, with reductions in BMI (2 studies (Sim *et al.*, 2014; Einarsson *et al.*, 2017), 348 women, MD [95% CI]: −1.68 kg m^−2^ [−2.54, −0.82], *I*^2^ = 83.31%) and waist circumference (1 study (Sim *et al.*, 2014), 43 women, MD [95% CI]: −1.35 cm [−2.02, −0.69]). No studies restricted to women with obesity reported on other anthropometric outcomes.

##### Metabolic outcomes

Lifestyle interventions significantly decreased fasting blood glucose (5 studies (Becker *et al.*, 2015; Mutsaerts *et al.*, 2016; Kiel *et al.*, 2018; Leblanc *et al.*, 2021; Phelan *et al.*, 2023), 712 women, MD [95% CI]: −0.15 mmol/l [−0.25, −0.04], *I*^2^ = 0.00%, moderate certainty evidence), total cholesterol (3 studies (Becker *et al.*, 2015; Mutsaerts *et al.*, 2016; Kiel *et al.*, 2018), 615 women, MD [95% CI]: −0.14 mmol/l [−0.27, −0.01], *I*^2^ = 0.00%), triglycerides (4 studies, 678 women, MD [95% CI]: −0.22 mmol/l [−0.35, −0.10], *I*^2^ = 0.00%), and the odds of having metabolic syndrome at 6 months after randomization (1 study (Mutsaerts *et al.*, 2016), 577 women, OR [95% CI]: 0.52 [0.37, 0.74]). Lifestyle interventions did not significantly affect fasting insulin, low-density lipoprotein cholesterol, high-density lipoprotein cholesterol, heart rate, C-reactive protein, HOMA-IR (homeostatic model of insulin resistance), HOMA2-IR, haemoglobin A1c, systolic blood pressure, or diastolic blood pressure.

In sensitivity analysis restricted to studies on women with obesity, similar results to our main analysis were found, with no significant difference between intervention and control in heart rate, systolic blood pressure, or diastolic blood pressure. Sensitivity on other metabolic outcomes was not performed as no studies restricted to women with obesity reported these outcomes.

#### Secondary outcomes

##### Other fertility outcomes

Lifestyle intervention may increase the odds of natural conception but this did not reach statistical significance (OR [95% CI]: 1.71 [0.99, 2.95], *I*^2^ = 36.50%). Lifestyle intervention did not significantly affect the odds of conception after ART or pregnancy loss. One study (Mutsaerts *et al.*, 2016) reported time to pregnancy resulting in live birth, and reported a median time to pregnancy of 8.8 months (interquartile range 3.5–13.2 months) in the intervention group and 5.2 months (interquartile range 2.6–9.4 months) in the control group (*P* = 0.04). One study (Boedt *et al.*, 2023) reported time to ongoing pregnancy, which was similar between intervention and control groups (hazard ratio [95% CI]: 0.94 [0.63, 1.40], *P* = 0.75). No studies reported on menstrual regularity or ovulation.

##### Obstetric outcomes

Lifestyle intervention did not significantly affect gestational age at delivery or the odds of multiple pregnancy, ovarian hyperstimulation syndrome, pre-term birth, caesarean section, gestational hypertension, preeclampsia, gestational diabetes mellitus, shoulder dystocia, total perineal rupture, or postpartum haemorrhage.

##### Foetal outcomes

Lifestyle intervention decreased birth weight (7 studies (Lumley and Donohue, 2006; Mutsaerts *et al.*, 2016; Einarsson *et al.*, 2017; Rono *et al.*, 2018; Jiskoot *et al.*, 2020; Leblanc *et al.*, 2021; Boedt *et al.*, 2023), 1607 newborns, MD [95% CI]: −59.07 g [−105.34, −12.81] *I*^2^ = 19.12%) but did not significantly affect the odds of low birth weight, macrosomia, small for gestational age, large for gestational age, low Apgar score, congenital anomaly, admission to neonatal intensive care unit or neonatal mortality.

##### Infant and child outcomes

A follow-up study of two included studies (Mutsaerts *et al.*, 2016; Rono *et al.*, 2018) reported that, for both studies, there was no significant difference between intervention and control groups in offspring neurodevelopmental scores at the age of 3–6 years according to the Ages and Stages Questionnaire, either for the total questionnaire score or any subdomain scores.

##### Other outcomes

Lifestyle intervention significantly decreased leptin (1 study (Becker *et al.*, 2015), 23 women, MD [95% CI]: −21.13 ng/ml [−40.38, −1.88]) but did not significantly affect testosterone, prolactin, sex hormone binding globulin, free androgen index, or ghrelin. Four studies (Mutsaerts *et al.*, 2016; Mohseni *et al.*, 2021; Boedt *et al.*, 2023; Koduri *et al.*, 2024) reported on maternal quality of life, but used different scoring systems and were not included in meta-analysis. One study (Mutsaerts *et al.*, 2016) used the physical and mental component scores, and reported that physical quality of life was significantly higher with lifestyle intervention, but mental quality of life did not differ. One study (Mohseni *et al.*, 2021) used the modified version of the polycystic ovary syndrome health-related quality of life questionnaire (MPCOSQ), and reported that quality of life improved with lifestyle intervention. One study (Koduri *et al.*, 2024) used the polycystic ovary syndrome quality of life questionnaire, consisting of five domains (emotion, body hair, weight, infertility, and menstrual irregularities), and reported no significant difference between intervention and control groups in any domain of the questionnaire following lifestyle intervention. One study (Boedt *et al.*, 2023) used the fertility-related quality of life questionnaire (FERTIQOL) and reported no significant difference between intervention and control groups following lifestyle intervention. No studies reported on maternal mortality.

## Discussion

### Summary of key findings

In this systematic review and meta-analysis, we report for the first time on the association of intervention characteristics and BCTs with the effects of preconception lifestyle interventions. Despite no overall effect of lifestyle interventions on odds of clinical pregnancy or live birth, we identified that delivering lifestyle interventions over 10 or more sessions and including the BCT *Adding objects to the environment* was associated with increased odds of clinical pregnancy. Additionally, weight loss induced by preconception lifestyle interventions was greater for women with infertility compared to without infertility. Intervention characteristics associated with greater weight loss include face-to-face intervention delivery and a weight loss aim. Significant subgroup differences such as associations of intervention format with odds of clinical pregnancy and intervention format and certain BCTs (*Action planning*, *Review outcome goal(s)*, *Monitoring of behaviour by others without feedback*, *Social support (emotional)*, and *Information about social and environmental consequences*) with weight loss should be interpreted with caution due to small subgroup sizes.

### Interpretation and implications

We report interventions including the BCT *Adding objects to the environment* (e.g. provision of intervention-compliant food, meal replacement products, exercise equipment and/or self-monitoring tools) and delivery of over 10 or more sessions were associated with higher odds of clinical pregnancy. *Adding objects to the environment* may assist intervention compliance by overcoming barriers to lifestyle management experienced by people with infertility, including limited time, money, and resources (Torkel *et al.*, 2024). Implementing this BCT in a real-world clinical setting may be challenging contingent on resource availability; potential real-world applications could include repurposing existing objects or providing shopping lists of intervention-compliant products which are appropriate for patients’ circumstances. We additionally report that interventions delivered over 10 or more sessions were associated with higher odds of clinical pregnancy, but note that increasing the number of sessions will increase health care costs and similarly may be challenging in a real-world setting. Further research is needed to elucidate the dose–response relationship between number of sessions and effect of the intervention on odds of pregnancy, in order to inform decision-making on balancing cost and effectiveness. Our findings suggest that preconception lifestyle interventions aiming to enhance fertility should consider a structured and more intensive approach, where clinically appropriate and feasible. Our systematic review also showed potentially increased natural conception rates following lifestyle interventions although the CIs just crossed 1. This is biologically plausible due to the improved menstrual cycle regularity and ovulation for those with ovulation disorders (Hakimi and Cameron, 2017; Ruiz-González *et al.*, 2024), but the findings would need to be confirmed in future studies.

Consistent with prior findings (Boedt *et al.*, 2021b; Ruiz-González *et al.*, 2024), we report preconception interventions improve anthropometric outcomes (weight, BMI, and waist circumference). We additionally report for the first time that preconception interventions in women with infertility achieved greater weight loss compared to those without infertility, consistent with qualitative literature identifying the desire to enhance fertility as a strong motivator for preconception weight loss (Pico *et al.*, 2023). Furthermore, although interventions aiming to reduce weight resulted in greater weight loss, those without weight loss as a stated aim still achieved weight loss (MD −0.81 kg, 95% CI −1.48 kg to −0.14 kg) likely due to improvements in diet and physical activity. Women with infertility have an elevated risk of eating disorders and disordered eating (Hecht *et al.*, 2022), and unsuccessful weight loss attempts can induce shame and self-blame for women with infertility, exacerbating stress during the infertility journey (Porter and Bhattacharya, 2008). Non-weight-centric preconception lifestyle interventions could circumvent these adverse consequences, while still potentially inducing favourable anthropometric changes. Additionally, observational studies report healthy dietary patterns, including the Mediterranean diet, can promote fertility independently of weight (Karayiannis *et al.*, 2018; Sun *et al.*, 2019) with further interventional research needed.

The inclusion of technology in interventions decreased the magnitude of weight loss which may reflect reduced in-person contact. Nevertheless, technology-supported interventions still reduced weight (MD −2.21 kg, 95% CI −3.62 kg to −0.81 kg). Telehealth can enhance health service productivity (Law *et al.*, 2024) and health equity for individuals living in rural and remote communities (Butzner and Cuffee, 2021) while reducing health service costs (Carrandi *et al.*, 2023) and logistical challenges (Boedt *et al.*, 2021a). The decision to include technology in preconception lifestyle interventions should therefore consider patient circumstances, resource availability, and intended effect on weight. Additionally, the inclusion of some BCTs relating to goals and planning (e.g. goal setting and problem solving) was associated with interventions being less effective at reducing weight, contrasting prior findings where these BCTs enhanced effectiveness of lifestyle interventions in adults above a healthy weight (Samdal *et al.*, 2017). This difference in findings may reflect unique challenges experienced by preconceptional women, such as feeling stressed and overwhelmed about entering an unfamiliar life stage (Khan *et al.*, 2019), potentially resulting in improved effectiveness of structured support in favour of self-guided support. Therefore, careful consideration is needed to limit burden of lifestyle interventions and avoid exacerbating stress in preconceptional women.

We report for the first time in a meta-analysis that preconception lifestyle interventions increased the odds of excessive gestational weight gain (OR 1.67, 95% CI 1.07–2.59), consistent with large observational studies (Piccinini-Vallis *et al.*, 2021; Yu *et al.*, 2024). However, we note that only two studies were included in the meta-analysis for gestational weight gain (Leblanc *et al.*, 2021; Phelan *et al.*, 2023), and hence, further research is needed to confirm our findings. Mechanisms for this elevated risk of excessive gestational weight gain may be similar to well-studied mechanisms for weight regain after weight loss in the general population, including changes in gut hormones, adipokines, appetite, and energy expenditure (Sumithran and Proietto, 2013; Van Baak and Mariman, 2019). Excessive gestational weight gain is a risk factor for adverse maternal and infant outcomes including macrosomia, gestational diabetes mellitus, and caesarean delivery (Goldstein *et al.*, 2017; Jin *et al.*, 2019; Baran *et al.*, 2020). This increased risk of excessive gestational weight gain may have counteracted beneficial intervention effects on weight, glucose, and lipids (Baumfeld *et al.*, 2015; Kim *et al.*, 2023), accounting for lack of intervention differences in pregnancy and neonatal outcomes, except for a subtle difference in birth weight. It may therefore be useful to expand preconception interventions into antenatal care, provide advice on optimal lifestyle and weight gain in pregnancy (Shieh *et al.*, 2018), and report on excessive gestational weight gain in future research. These are important considerations for all women undertaking preconception lifestyle interventions given gestational weight gain was similar for women with modest or substantial preconception weight loss (Price *et al.*, 2021).

### Strengths and limitations

Our study has several strengths. We used a comprehensive search strategy for study identification and included studies across all languages. Additionally, we assessed intervention characteristics and BCTs using established frameworks, and reviewers undertook training on BCTs, enhancing the rigour of our exploration of sources of heterogeneity. Our findings should be considered in light of several limitations. Firstly, subgroup analyses should be interpreted with caution due to multiple testing and because they were not based on randomized comparisons with unequal subgroup sizes in some instances, with very few interventions being delivered in a group format or reporting certain BCTs. Potential underreporting of BCTs, identified in prior research (De Bruin *et al.*, 2021), may have affected our results. Additionally, although live birth was a primary outcome for our review and part of the core outcome set for infertility research (Duffy *et al.*, 2020), the number of studies included in meta-analysis for live birth was insufficient for meaningful subgroup analysis (Chandler *et al.*, 2019). Similarly, the number of studies limited our ability to determine the association of certain population characteristics (e.g. types of infertility) or intervention types (e.g. different dietary or physical activity strategies) with the effects of the interventions. We additionally note that the short-term lifestyle interventions in our review were unable to determine the effects of long-term lifestyle behaviours; there is evidence that childhood diet and environmental exposures have lasting impacts on fertility and health (Harville *et al.*, 2021), but these were not measured in the studies included in our review. These limitations highlight the need for more high-quality RCTs on preconception lifestyle interventions, and for those aiming to improve fertility to report outcomes in accordance with the core outcome set for infertility research (Duffy *et al.*, 2020). Finally, findings based on subgroup analyses should be interpreted with caution and warrant further investigation due to the exploratory nature of the analysis, limited number of studies included, and potential aggregation bias of study-level subgroup effects. The individual participant data meta-analysis on this topic may overcome some limitations due to the use of aggregate data and shed light on the subgroup effects (Evans-Hoeker *et al.*, 2022).

## Conclusion

Lifestyle interventions for preconceptional women result in favourable anthropometric and metabolic changes without significantly affecting pregnancy rates, live birth rates, offspring neurodevelopment, or the risk of pregnancy complications or adverse neonatal outcomes. Favourable anthropometric changes were particularly pronounced in face-to-face interventions with a stated aim of weight loss. Greater effectiveness on fertility was observed with studies using a structured and more intensive approach to lifestyle intervention. Further research is needed to elucidate the effects of different intervention characteristics and dietary and physical activity strategies on the health and fertility of preconceptional women.

## Supplementary Material

dmaf021_Supplementary_Data

## Data Availability

The data underlying this article will be shared on reasonable request to the corresponding author.
