# Supplementary material for: Preconception lifestyle interventions for women—a systematic review and meta-analysis of intervention characteristics and behaviour change techniques
Source: Hum Reprod Update. 2025 Aug 22;32(1):105–27. doi: 10.1093/humupd/dmaf021 (PMC12766448; doi:10.1093/humupd/dmaf021)
Supplement: dmaf021_Supplementary_Data [file dmaf021_Supplementary_Data.zip › HRU-24-0041.R3 Supplementary Data Final.pdf]

Preconception lifestyle interventions for women – a systematic review and meta-analysis of intervention characteristics and behaviour change techniques

Authors: Sophia Torkel[1], Evangeline Mantzioris[2], Anthony Villani[3], Nicole J Kellow[4], Dhruv Bhatnagar[1], Elaine K Osei-Safo[1], Margaret McGowan[1], Nur K. Abdul Jafar[1], Nadia Bogatzke[5], Simon Alesi[1], Tuba Astarcioglu[1], Ben W Mol[6], Robert J Norman[7], Stephanie Cowan[1], Rui Wang[8]†, Lisa Moran[1]†*

[1]Monash Centre for Health Research and Implementation, Monash University, Clayton, Australia
[2]Clinical and Health Sciences and Alliance for Research in Exercise, Nutrition and Activity (ARENA), University of South Australia, Adelaide, Australia
[3]School of Health, University of the Sunshine Coast, Sippy Downs, Australia
[4]Department of Nutrition, Dietetics & Food, Notting Hill, Australia
[5]Monash Health, Clayton, Australia
[6]Department of Obstetrics and Gynaecology, Monash University, Clayton, Australia
[7]Robinson Research Institute, University of Adelaide, Adelaide, Australia
[8]NHMRC Clinical Trials Centre, University of Sydney, Sydney, Australia.

†Joint senior authors
*Correspondence address: Monash Centre for Health Research and Implementation, 43-51 Kanooka Grove, Clayton, VIC 3168, Australia. Tel: +61 3 8572 2854; Fax: +61 3 9594 7554. Email: lisa.moran@monash.edu. https://orcid.org/0000-0001-5772-6484

Supplementary Data Table of Contents

Supplementary Table S1. Search strategy in Ovid Medline 2
Supplementary Table S2. Search strategy in Ovid PsycINFO 4
Supplementary Table S3. Search strategy in Embase and Emcare 6
Supplementary Table S4. Search strategy in Scopus 8
Supplementary Table S5. Search strategy in Cochrane Central Register of Controlled Trials 9
Supplementary Table S6. Search strategy in CINAHL 11
Supplementary Table S7. Eligibility criteria 13
Supplementary Table S8. Studies excluded and awaiting classification 15
Supplementary Table S9. Intervention characteristics according to the TIDieR checklist. 17
Supplementary Table S10. Behaviour change techniques utilised in studies 33
Supplementary Table S11. Results from pooled meta-analysis. 36
Supplementary Table S12. Summary of findings table. 44
Supplementary Table S13. Results for sensitivity analysis restricted to women with obesity. 46
Supplementary Figure S1. Contour-enhanced funnel plot for clinical pregnancy 47
Supplementary Figure S2. Contour-enhanced funnel plot for weight 48
References 49

Supplementary Table S1. Search strategy in Ovid Medline

1.	(preconcep$ or pre concep$ or pre-concep$).tw,kf.
2.	(prepregnan$ or pre pregnan$ or pre-pregnan$).tw,kf.
3.	(pregestation$ or pre gestation$ or pre-gestation$).tw,kf.
4.	(periconception$ or peri conception$ or peri-conception$).tw,kf.
5.	(interconcep$ or inter concep$ or inter-concep$).tw,kf.
6.	(interpregnan$ or inter pregnan$ or inter-pregnan$).tw,kf.
7.	(intergestation$ or inter gestation$ or inter-gestation$).tw,kf.
8.	(internatal or inter natal or inter-natal).tw,kf.
9.	before pregnan$.tw,kf.
10.	Preconception Care/
11.	Family Planning Services/
12.	(icsi or intracytoplasmic sperm injection).tw,kf.
13.	(ivf or in vitro fertili*).tw,kf.
14.	(iui or intrauterine insemination).tw,kf.
15.	assisted reproduct*.tw,kf.
16.	artificial insemination.tw,kf.
17.	ovarian hyperstimulation.tw,kf.
18.	(infertil* or subfertil* or fertility).tw,kf.
19.	reproducti* techniques.tw,kf.
20.	infertility/ or infertility, female/
21.	exp Reproductive Techniques, Assisted/
22.	1 or 2 or 3 or 4 or 5 or 6 or 7 or 8 or 9 or 10 or 11 or 12 or 13 or 14 or 15 or 16 or 17 or 18 or 19 or 20 or 21
23.	exp Diet/
24.	nutrition therapy/ or exp diet therapy/
25.	exp Feeding Behavior/
26.	diet$.tw,kf.
27.	nutrition$.tw,kf.
28.	(energy adj3 restrict$).tw,kf.
29.	(energy adj3 reduc$).tw,kf.
30.	hypocaloric.tw,kf.
31.	feeding behavio$.tw,kf.
32.	eating behavio$.tw,kf.
33.	exp Exercise/
34.	exp Exercise Therapy/
35.	Yoga/
36.	exercis$.tw,kf.
37.	physical activit$.tw,kf.
38.	physical performance.tw,kf.
39.	physical train$.tw,kf.
40.	(strength adj2 train$).tw,kf.
41.	(resistance adj2 train$).tw,kf.
42.	(aerobic adj2 train$).tw,kf.
43.	kinesiotherap$.tw,kf.
44.	sport$.tw,kf.
45.	vigorous activit$.tw,kf.
46.	moderate activit$.tw,kf.
47.	resistance program$.tw,kf.
48.	resistance regime$.tw,kf.
49.	aerobic capacity.tw,kf.
50.	Weight Loss/

51.	((weight or BMI or body mass index) adj3 (preser$ or maintain$ or maintenance or reduc$ or los$ or decreas$ or control or manage$)).tw,kf.
52.	exp Life Style/
53.	(lifestyle or life style or life-style).tw,kf.
54.	23 or 24 or 25 or 26 or 27 or 28 or 29 or 30 or 31 or 32 or 33 or 34 or 35 or 36 or 37 or 38 or 39 or 40 or 41 or 42 or 43 or 44 or 45 or 46 or 47 or 48 or 49 or 50 or 51 or 52 or 53
55.	randomized controlled trial.pt.
56.	controlled clinical trial.pt.
57.	randomized.ab.
58.	placebo.ab.
59.	randomly.ab.
60.	trial.ab.
61.	groups.ab.
62.	55 or 56 or 57 or 58 or 59 or 60 or 61
63.	exp animals/ not humans.sh.
64.	62 not 63
65.	22 and 54 and 64

Supplementary Table S2. Search strategy in Ovid PsycINFO

1.	(preconcep$ or pre concep$ or pre-concep$).tw.
2.	(prepregnan$ or pre pregnan$ or pre-pregnan$).tw.
3.	(pregestation$ or pre gestation$ or pre-gestation$).tw.
4.	(periconception$ or peri conception$ or peri-conception$).tw.
5.	(interconcep$ or inter concep$ or inter-concep$).tw.
6.	(interpregnan$ or inter pregnan$ or inter-pregnan$).tw.
7.	(intergestation$ or inter gestation$ or inter-gestation$).tw.
8.	(internatal or inter natal or inter-natal).tw.
9.	before pregnan$.tw.
10.	family planning/
11.	(icsi or intracytoplasmic sperm injection).tw.
12.	(ivf or in vitro fertili*).tw.
13.	(iui or intrauterine insemination).tw.
14.	assisted reproduct*.tw.
15.	artificial insemination.tw.
16.	ovarian hyperstimulation.tw.
17.	(infertil* or subfertil* or fertility).tw.
18.	reproducti* techniques.tw.
19.	exp infertility/
20.	exp fertility/
21.	reproductive technology/
22.	or/1-21
23.	exp diets/
24.	exp nutrition/
25.	exp eating behavior/
26.	diet$.tw.
27.	nutrition$.tw.
28.	(energy adj3 restrict$).tw.
29.	(energy adj3 reduc$).tw.
30.	hypocaloric.tw.
31.	feeding behavio$.tw.
32.	eating behavio$.tw.
33.	exp physical activity/
34.	exp physical endurance/
35.	physical fitness/
36.	exercis$.tw.
37.	physical activit$.tw.
38.	physical performance.tw.
39.	physical train$.tw.
40.	(strength adj2 train$).tw.
41.	(resistance adj2 train$).tw.
42.	(aerobic adj2 train$).tw.
43.	kinesiotherap$.tw.
44.	sport$.tw.
45.	vigorous activit$.tw.
46.	moderate activit$.tw.
47.	resistance program$.tw.
48.	resistance regime$.tw.
49.	aerobic capacity.tw.
50.	weight loss/

51.	((weight or BMI or body mass index) adj3 (preser$ or maintain$ or maintenance or reduc$ or los$ or decreas$ or control or manage$)).tw.
52.	exp lifestyle/
53.	(lifestyle or life style or life-style).tw.
54.	or/23-53
55.	("double-blind" or "random* assigned" or control).tw.
56.	22 and 54 and 55

Supplementary Table S3. Search strategy in Embase and Emcare

1.	(preconcep$ or pre concep$ or pre-concep$).tw,kf.
2.	(prepregnan$ or pre pregnan$ or pre-pregnan$).tw,kf.
3.	(pregestation$ or pre gestation$ or pre-gestation$).tw,kf.
4.	(periconception$ or peri conception$ or peri-conception$).tw,kf.
5.	(interconcep$ or inter concep$ or inter-concep$).tw,kf.
6.	(interpregnan$ or inter pregnan$ or inter-pregnan$).tw,kf.
7.	(intergestation$ or inter gestation$ or inter-gestation$).tw,kf.
8.	(internatal or inter natal or inter-natal).tw,kf.
9.	before pregnan$.tw,kf.
10.	prepregnancy care/
11.	family planning/
12.	(icsi or intracytoplasmic sperm injection).tw,kf.
13.	(ivf or in vitro fertili*).tw,kf.
14.	(iui or intrauterine insemination).tw,kf.
15.	assisted reproduct*.tw,kf.
16.	artificial insemination.tw,kf.
17.	ovarian hyperstimulation.tw,kf.
18.	(infertil* or subfertil* or fertility).tw,kf.
19.	reproducti* techniques.tw,kf.
20.	exp infertility/
21.	exp infertility therapy/
22.	or/1-21
23.	exp nutrition/
24.	exp diet therapy/
25.	(energy adj3 restrict$).tw,kf.
26.	(energy adj3 reduc$).tw,kf.
27.	hypocaloric.tw,kf.
28.	feeding behavio$.tw,kf.
29.	eating behavio$.tw,kf.
30.	"physical activity, capacity and performance"/ or endurance/ or exp exercise/ or metabolic equivalent/ or exp physical activity/ or exp physical capacity/ or exp physical performance/
31.	exp kinesiotherapy/
32.	exercis$.tw,kf.
33.	physical activit$.tw,kf.
34.	physical performance.tw,kf.
35.	physical train$.tw,kf.
36.	(strength adj2 train$).tw,kf.
37.	(resistance adj2 train$).tw,kf.
38.	(aerobic adj2 train$).tw,kf.
39.	kinesiotherap$.tw,kf.
40.	sport$.tw,kf.
41.	vigorous activit$.tw,kf.
42.	moderate activit$.tw,kf.
43.	resistance program$.tw,kf.
44.	resistance regime$.tw,kf.
45.	aerobic capacity.tw,kf.
46.	exp body weight management/
47.	body weight loss/
48.	((weight or BMI or body mass index) adj3 (preser$ or maintain$ or maintenance or reduc$ or los$ or decreas$ or control or manage$)).tw,kf.
49.	exp lifestyle/ or lifestyle modification/

50.	(lifestyle or life style or life-style).tw,kf.
51.	or/23-50
52.	clinical trial/
53.	randomized controlled trial/
54.	randomization/
55.	single blind procedure/
56.	double blind procedure/
57.	crossover procedure/
58.	placebo/
59.	randomi?ed controlled trial$.tw.
60.	rct.tw.
61.	random allocation.tw.
62.	randomly allocated.tw.
63.	allocated randomly.tw.
64.	(allocated adj2 random).tw.
65.	single blind$.tw.
66.	double blind$.tw.
67.	((treble or triple) adj blind$).tw.
68.	Placebo$.tw.
69.	Prospective study/
70.	or/52-69
71.	case study/
72.	case report.tw.
73.	abstract report/ or letter/
74.	or/71-73
75.	70 not 74
76.	22 and 51 and 75

Supplementary Table S4. Search strategy in Scopus

1.	(TITLE-ABS-KEY (preconcep* OR "pre concep*" OR pre-concep* OR prepregnan* OR "pre pregnan*" OR pre-pregnan* OR pregestation* OR "pre gestation*" OR pre-gestation* OR periconception* OR "peri conception*" OR peri-conception* OR interconcep* OR "inter concep*" OR inter-concep* OR interpregnan* OR "inter pregnan*" OR inter-pregnan* OR intergestation* OR "inter gestation*" OR inter-gestation* OR internatal OR "inter natal" OR inter-natal OR "before pregnan*" OR icsi OR "intracytoplasmic sperm injection" OR ivf OR "in vitro fertili*" iui OR "intrauterine insemination" OR "assisted reproduct*" OR "artificial insemination" OR "ovarian hyperstimulation" OR infertil* OR subfertil* OR fertility OR "reproducti* techniques") AND TITLE-ABS-KEY (diet* OR nutrition* OR (energy W/2 restrict*) OR (energy W/2 reduc*) OR hypocaloric OR "feeding behavio*" OR "eating behavio*" OR "exercis*" OR "physical activit*" OR "physical performance" OR "physical train*" OR (strength W/1 train*) OR (resistance W/1 train*) OR (aerobic W/1 train*) OR kinesiotherap* OR sport* OR "vigorous activit*" OR "moderate activit*" OR "resistance program*" OR "resistance regime*" OR "aerobic capacity" OR ((weight OR bmi OR "body mass index") W/2 (preser* OR maintain* OR maintenance OR reduc* OR los* OR decreas* OR control OR manage*)) OR lifestyle OR "life style" OR life-style)) AND (INDEXTERMS ("clinical trials" OR "clinical trials as a topic" OR "randomized controlled trial" OR "Randomized Controlled Trials as Topic" OR "controlled clinical trial" OR "Controlled Clinical Trials" OR "random allocation" OR "Double-Blind Method" OR "Single-Blind Method" OR "Cross-Over Studies" OR "Placebos" OR "multicenter study" OR "double blind procedure" OR "single blind procedure" OR "crossover procedure" OR "clinical trial" OR "controlled study" OR "randomization" OR "placebo")) OR (TITLE-ABS-KEY ("clinical trials" OR "clinical trials as a topic" OR "randomized controlled trial" OR "Randomized Controlled Trials as Topic" OR "controlled clinical trial" OR "Controlled Clinical Trials as Topic" OR "random allocation" OR "randomly allocated" OR "allocated randomly" OR "Double-Blind Method" OR "Single-Blind Method" OR "Cross-Over Studies" OR "Placebos" OR "cross-over trial" OR "single blind" OR "double blind" OR "factorial design" OR "factorial trial")) OR (TITLE-ABS (clinical AND trial* OR trial* OR rct* OR random* OR blind*))

Supplementary Table S5. Search strategy in Cochrane Central Register of Controlled Trials

1.	(preconcep$ or pre concep$ or pre-concep$).tw,kf.
2.	(prepregnan$ or pre pregnan$ or pre-pregnan$).tw,kf.
3.	(pregestation$ or pre gestation$ or pre-gestation$).tw,kf.
4.	(periconception$ or peri conception$ or peri-conception$).tw,kf.
5.	(interconcep$ or inter concep$ or inter-concep$).tw,kf.
6.	(interpregnan$ or inter pregnan$ or inter-pregnan$).tw,kf.
7.	(intergestation$ or inter gestation$ or inter-gestation$).tw,kf.
8.	(internatal or inter natal or inter-natal).tw,kf.
9.	before pregnan$.tw,kf.
10.	Preconception Care/
11.	Family Planning Services/
12.	(icsi or intracytoplasmic sperm injection).tw,kf.
13.	(ivf or in vitro fertili*).tw,kf.
14.	(iui or intrauterine insemination).tw,kf.
15.	assisted reproduct*.tw,kf.
16.	artificial insemination.tw,kf.
17.	ovarian hyperstimulation.tw,kf.
18.	(infertil* or subfertil* or fertility).tw,kf.
19.	reproducti* techniques.tw,kf.
20.	infertility/ or infertility, female/
21.	exp Reproductive Techniques, Assisted/
22.	1 or 2 or 3 or 4 or 5 or 6 or 7 or 8 or 9 or 10 or 11 or 12 or 13 or 14 or 15 or 16 or 17 or 18 or 19 or 20 or 21
23.	exp Diet/
24.	nutrition therapy/ or exp diet therapy/
25.	exp Feeding Behavior/
26.	diet$.tw,kf.
27.	nutrition$.tw,kf.
28.	(energy adj3 restrict$).tw,kf.
29.	(energy adj3 reduc$).tw,kf.
30.	hypocaloric.tw,kf.
31.	feeding behavio$.tw,kf.
32.	eating behavio$.tw,kf.
33.	exp Exercise/
34.	exp Exercise Therapy/
35.	Yoga/
36.	exercis$.tw,kf.
37.	physical activit$.tw,kf.
38.	physical performance.tw,kf.
39.	physical train$.tw,kf.
40.	(strength adj2 train$).tw,kf.
41.	(resistance adj2 train$).tw,kf.
42.	(aerobic adj2 train$).tw,kf.
43.	kinesiotherap$.tw,kf.
44.	sport$.tw,kf.
45.	vigorous activit$.tw,kf.
46.	moderate activit$.tw,kf.
47.	resistance program$.tw,kf.
48.	resistance regime$.tw,kf.
49.	aerobic capacity.tw,kf.
50.	Weight Loss/

51.	((weight or BMI or body mass index) adj3 (preser$ or maintain$ or maintenance or reduc$ or los$ or decreas$ or control or manage$)).tw,kf.
52.	exp Life Style/
53.	(lifestyle or life style or life-style).tw,kf.
54.	23 or 24 or 25 or 26 or 27 or 28 or 29 or 30 or 31 or 32 or 33 or 34 or 35 or 36 or 37 or 38 or 39 or 40 or 41 or 42 or 43 or 44 or 45 or 46 or 47 or 48 or 49 or 50 or 51 or 52 or 53
55.	22 and 54

Supplementary Table S6. Search strategy in CINAHL

S1	(MH "Prepregnancy Care")
S2	(MH "Family Planning")
S3	preconcep* OR pre-concep* OR "pre concep*"
S4	TI (preconcep* or pre-concep* or "pre concep*") OR AB (preconcep* or pre-concep* or "pre concep*")
S5	TI (prepregnan* or pre-pregnan* or "pre pregnan*") OR AB (prepregnan* or pre-pregnan* or "pre pregnan*")
S6	TI (pregestation$ or pre-gestation$ or "pre gestation$") OR AB (pregestation$ or pre-gestation$ or "pre gestation$")
S7	TI (periconception* or peri-conception* or "peri conception*") OR AB (periconception* or peri-conception* or "peri conception*")
S8	TI (interconcep* or inter-concep* or "inter concep*") OR AB (interconcep* or inter-concep* or "inter concep*")
S9	TI (interpregnan* or inter-pregnan* or "inter pregnan*") OR AB (interpregnan* or inter-pregnan* or "inter pregnan*")
S10	TI (intergestation* or inter-gestation* or "inter gestation*") OR AB (intergestation* or inter-gestation* or "inter gestation*")
S11	TI (internatal or inter-natal or "inter natal") OR AB (internatal or inter-natal or "inter natal")
S12	TI "before pregnan*" OR AB "before pregnan*"
S13	(MH "Prepregnancy Care")
S14	(MH "Family Planning")
S15	TI (icsi or "intracytoplasmic sperm injection") OR AB (icsi or "intracytoplasmic sperm injection")
S16	TI (ivf or "in vitro fertili*") OR AB (ivf or "in vitro fertili*")
S17	TI "assisted reproduct*" OR AB "assisted reproduct*"
S18	TI "artificial insemination" OR AB "artificial insemination"
S19	TI "ovarian hyperstimulation" OR AB "artificial insemination"
S20	TI (infertil* or subfertil* or fertility) OR AB (infertil* or subfertil* or fertility)
S21	TI "reproducti* techniques" OR AB "reproducti* techniques"
S22	(MH "Infertility")
S23	(MH "Reproduction Techniques+")
S24	S4 OR S5 OR S6 OR S7 OR S8 OR S9 OR S10 OR S11 OR S12 OR S13 OR S14 OR S15 OR S16 OR S17 OR S18 OR S19 OR S20 OR S21 OR S22 OR S23
S25	(MH "Diet+")
S26	(MH "Diet Therapy+")
S27	(MH "Eating Behavior+")
S28	TI diet* OR AB diet*
S29	TI nutrition* OR AB nutrition*
S30	TI energy N2 restrict* OR AB energy N2 restrict*
S31	TI energy N2 reduc* OR AB energy N2 reduc*
S32	TI hypocaloric OR AB hypocaloric
S33	TI "feeding behavio*" OR AB "feeding behavio*"
S34	(MH "Exercise+") OR (MH "Sports+") OR (MH "Physical Activity") OR (MH "Physical Fitness+") OR (MH "Physical Performance")
S35	(MH "Therapeutic Exercise+")
S36	(MH "Yoga+") OR (MH "Tai Chi") OR (MH "Dance Therapy")
S37	TI exercis* OR AB exercis*
S38	TI "physical activit*" OR AB "physical activit*"
S39	TI "physical performance*" OR AB "physical performance*"
S40	TI "physical train*" OR AB "physical train*"
S41	TI strength N1 train* OR AB strength N1 train*

S42	TI resistance N1 train* OR AB resistance N1 train*
S43	TI aerobic N1 train* OR AB aerobic N1 train*
S44	TI kinesiotherap* OR AB kinesiotherap*
S45	TI sport* OR AB sport*
S46	TI "vigorous activit*" OR AB "vigorous activit*"
S47	TI "moderate activit*" OR AB "moderate activit*"
S48	TI "resistance program*" OR AB "resistance program*"
S49	TI "resistance regime*" OR AB "resistance regime*"
S50	TI "aerobic capacity" OR AB "aerobic capacity"
S51	(MH "Weight Loss")
S52	TI ((weight or BMI or "body mass index") N2 (preserv* or maintain* or maintenance or reduc* or los* or decreas* or control or manage*)) OR AB ((weight or BMI or "body mass index") N2 (preserv* or maintain* or maintenance or reduc* or los* or decreas* or control or manage*))
S53	(MH "Life Style+")
S54	TI (lifestyle or life-style or "life style") OR AB (lifestyle or life-style or "life style")
S55	S25 OR S26 OR S27 OR S28 OR S29 OR S30 OR S31 OR S32 OR S33 OR S34 OR S35 OR S36 OR S37 OR S38 OR S39 OR S40 OR S41 OR S42 OR S43 OR S44 OR S45 OR S46 OR S47 OR S48 OR S49 OR S50 OR S51 OR S52 OR S53 OR S54
S56	MH randomized controlled trials
S57	MH double-blind studies
S58	MH single-blind studies
S59	MH random assignment
S60	MH pretest-posttest design
S61	MH cluster sample
S62	TI (randomised OR randomized)
S63	AB (random*)
S64	TI (trial)
S65	MH (sample size) AND AB (assigned OR allocated OR control)
S66	MH (placebos)
S67	PT (randomized controlled trial)
S68	AB (control W5 group)
S69	MH (crossover design) OR MH (comparative studies)
S70	AB (cluster W3 RCT)
S71	MH animals+
S72	MH (animal studies)
S73	TI (animal model*)
S74	S71 OR S72 OR S73
S75	MH (human)
S76	S74 NOT S75
S77	S56 OR S57 OR S58 OR S59 OR S60 OR S61 OR S62 OR S63 OR S64 OR S65 OR S66 OR S67 OR S68 OR S69 OR S70
S78	S77 NOT S76
S79	S24 AND S55 AND S78

Supplementary Table S7. Eligibility criteria

	Inclusion	Exclusion
Population	Non-pregnant women (including women with infertility) of childbearing age (as defined by study authors; no exclusion based on age) with an intention to conceive. Women with concurrent medication use were eligible as long as medication use was appropriately documented and consistent between groups.	BMI <18 (to avoid studies pertaining to malnutrition and energy supplementation), AND/OR hereditary disorder in one or both of parents (specifically those that could affect fertility and gestation and foetal outcomes. (e.g cystic fibrosis, sickle cell, thalassemia, haemophilia, fragile X, Turner syndrome), AND/OR animal trials.
Intervention	Lifestyle modification aiming to optimize nutritional and/or physical activity status, including weight loss or weight maintenance or prevention of weight gain, Dietary/nutritional intervention, Exercise/physical activity, Psychosocial support or behavioural interventions	Trials focusing solely on micronutrient supplementation, alcohol, smoking cessation/reduction or diabetes control, AND/OR pharmacotherapies used in intervention group but not control group, or vice versa
Comparator	No intervention or standard minimal care (defined as no more than one session allocated to dietary and/or physical activity treatment)	Pharmacotherapies used in intervention group but not control group, or vice versa
Outcomes	Main outcomes:<br>- Live birth<br>- Clinical pregnancy<br>- Maternal anthropometric outcomes (e.g. weight, body mass index, waist circumference, waist to hip ratio)<br>- Maternal metabolic outcomes (e.g. blood pressure, glucose, insulin, lipids)<br><br>Fertility outcomes:<br>- Conception (natural or after ART)<br>- Time to conception<br>- Menstrual regularity<br>- Ovulation<br>- Pregnancy loss (ectopic pregnancy, miscarriage, stillbirth and termination of pregnancy)<br><br>Obstetric outcomes:<br>- Pre-eclampsia<br>- Gestational diabetes<br>- Gestational age at delivery (or pre - term labour)<br>- Delivery complications (e.g. prolonged labour, dystocia, breech presentation, cord prolapse/compression and amniotic fluid embolism)<br>- Caesarean section	N/A

	- Maternal post-partum complications (e.g. post-partum haemorrhage)<br>- ART adverse outcomes (e.g. single and multiple pregnancies/ovarian hyperstimulation syndrome)<br><br>Foetal outcomes:<br>- Intrauterine complications (e.g. intrauterine growth retardation and placental abnormality)<br>- Admission to neonatal intensive care<br>- Birth weight (or low-birth weight, macrosomia, small/large for gestational age)<br>- Major congenital anomaly or developmental complications (e.g. birth defects, low Apgar score)<br>- Neonatal mortality<br>Infant/Child Outcomes:<br>- Growth and development (both physical and mental development)<br><br>Other outcomes:<br>- Maternal quality of life<br>- Maternal hormonal profile (e.g. testosterone, SHBG, FSH, LH and prolactin)<br>- Maternal mortality	
Study design	Randomised controlled trials (either cluster or individually randomised)	Non-randomised studies, observational studies. Conference abstracts, clinical trial registrations and protocols were excluded unless a full-text article reporting on the results was also available.

ART, assisted reproductive technology; FSH, follicle stimulation hormone; LH, luteinising hormone; N/A, not applicable; SHBG, sex hormone binding globulin

Supplementary Table S8. Studies excluded and awaiting classification

Exclusion Reason	Citations
Ineligible population	(제남주 and 최소영 2016; Upadhyay et al. 2024; Potdar et al. 2014; Patten et al. 2022; Parsons et al. 2022; Oberg 2023; Mostajabi et al. 2022; Meenakshi et al. 2024; McBreairty, Chilibeck, et al. 2017; McBreairty et al. 2015; Mani et al. 2018; Liu et al. 2022; Liu and Yan 2022; Kiel et al. 2020; Heckert et al. 2019; Haakstad et al. 2021; Gangachin et al. 2024; Dashti et al. 2022; Benham et al. 2021; Barquiel et al. 2023; Aziz et al. 2021; Arentz et al. 2019; Abdollahi et al. 2019)
Ineligible intervention	(Young et al. 2023; Tavousi et al. 2024; Taneja et al. 2022; Szigeti et al. 2024; Sahariah et al. 2022; Sadeghi et al. 2021; Nga et al. 2020; Nery et al. 2019; Montanaro et al. 2023; Maunder et al. 2024; Maas et al. 2022; Krebs et al. 2021; Kiyak and Kocoglu-Tanyer 2021; Killeen et al. 2022; James et al. 2019; Hambidge et al. 2016, 2014; Dokras et al. 2016; Borengasser et al. 2018; Aziz et al. 2023)
Ineligible comparator	(Turner-McGrievy et al. 2014; Sordia-Hernandez et al. 2016; Sant'Anna et al. 2020; Rothberg et al. 2016; Price et al. 2021; Price et al. 2020; Price et al. 2018; Nagelberg et al. 2016; Muirhead et al. 2021; Kermack et al. 2021; Kermack et al. 2020; Brammall et al. 2022; Brammall et al. 2024; Alibeigi et al. 2020; Alibeigi et al. 2020)
Ineligible outcomes	(van Elten, Karsten, et al. 2018; van Dijk et al. 2020; Shabani et al. 2016; Pedro et al. 2019; Nourizadeh et al. 2020; Kirca and Pasinlioglu 2019; Hojeij, Schoenmakers, Willemsen, van Rossem, Dinnyes, Rousian, and Steegers-Theunissen 2023; Ghasemi Yngyknd et al. 2018; Dietz de Loos et al. 2023; Cena et al. 2008; Carmichael et al. 2019; Bastani et al. 2010)
Ineligible study design	(Wang, Groen, et al. 2021, 2021; van Oers et al. 2018; Van Elten, Van Poppel, et al. 2018; van Elten et al. 2019; van der Windt et al. 2021; Svensson et al. 2022; Salama et al. 2015; Raab et al. 2022; Palomba et al. 2008; Niederberger 2018, 2017; Nasrekani and Fathi 2016; Mogensen et al. 2023; Meneghini et al. 2023; Matsuzaki et al. 2017; Li 2023; Kaya et al. 2016; Karsten et al. 2019; Jamebozorg et al. 2023; Huvinen et al. 2020; Hollmann et al. 1996; Hojeij, Schoenmakers, Willemsen, van Rossem, Dinnyes, Rousian, Steegers-Theunissen, et al. 2023; Heerman et al. 2020; Hammiche et al. 2011; Halpern et al. 2013; Gorczyca et al. 2022; Gootjes et al. 2019; Doss 2017; Clark et al. 1998; Borthakur et al. 2023; Beena and Thomas 2016)
Ineligible publication type (conference abstract, protocol or clinical trial registration)	(Williams et al. 2016; Wang, Groen, Va Zomeren, et al. 2021; Van Oers et al. 2014; Tolahunase, Sagar, et al. 2018; Tolahunase, Kumar, et al. 2018; Timmermans et al. 2019; Thibodeau et al. 2024; Tate et al. 2018; Taneja et al. 2020; Talluto 2002; Syndrome 2020; Sujan et al. 2024, 2023; Sujan et al. 2023; Steegers-Theunissen 2018; Soepnel et al. 2022; Shapiro et al. 2024; Sauder et al. 2023; Sant'Anna et al. 2017; Rouissi, Levesque, et al. 2020; Rouissi, Jean-Denis, et al. 2020; Rochester et al. 2024; RBR-10ftp9pm 2024; RBR-7by76r 2016; Radin et al. 2016; Price et al. 2019; Pfuller et al. 2004; Paratmanitya et al. 2023; PACTR202206710963782 2022, 2017; Overby et al. 2021; Oostingh et al. 2018; Oostingh et al. 2019; NTR2450 2010; Norris et al. 2022; NL-OMON39189 2008; NL-OMON21869 2021; Ng et al. 2019; NCT06591637 2024; NCT06402825 2024; NCT06175520 2023; NCT06100523 2023; NCT06049186 2023; NCT06044545 2023; NCT05798494 2023; NCT05708937 2023; NCT05674799 2022; NCT05651568 2022; NCT05629858 2022; NCT05621109 2022; NCT05578690 2022; NCT05299450 2022; NCT05084274 2021; NCT04998591 2021; NCT04976881 2021; NCT04942457 2021, 2021; NCT04589793 2020; NCT04585581 2020; NCT04419740 2019; NCT04416620 2020; NCT04335227 2020; NCT04275869 2020; NCT04273048 2019; NCT04242069 2020; NCT03908099 2019; NCT03898037 2019; NCT03790449 2018; NCT03703115 2018; NCT03395067 2017; NCT03343405 2017; NCT03146156 2017; NCT03085212 2017;

	NCT03023137 2017; NCT03012412 2016; NCT02945488 2016; NCT02763150 2016; NCT02648555 2016; NCT02630485 2015; NCT02617693 2015; NCT02049554 2014; NCT01952795 2013; NCT01933633 2013; NCT01894074 2013; NCT01483612 2011; NCT01028989 2009; NCT00679679 2008; Nayar et al. 2023; Nayar et al. 2018; Nayar et al. 2017; Mutsaerts et al. 2016; Moore et al. 2016; Mol et al. 2015; Mead et al. 2024; McBreairty, Kazemi, et al. 2017; McBreairty et al. 2014; Malhotra et al. 2023; Malekpour et al. 2023; Maillet et al. 2021; Maas et al. 2020; Ly et al. 2017; Legro et al. 2014; LeBlanc, Boisvert, et al. 2021; Lawande et al. 2018; Kumaran et al. 2021; Kumaran et al. 2023; Krebs et al. 2023; Keytash 2015; Kermack et al. 2017; Kermack et al. 2014; Kermack et al. 2020; KCT0008950 2023; KCT0008931 2023; KCT0008022 2022; JPRN-UMIN000027424 2017; Jamebozorg et al. 2018; Jackson et al. 2022; ISRCTN97130017 2023; ISRCTN44294662 2021; ISRCTN13308752 2019; ISRCTN11081163 2016; ISRCTN01915371 2011; IRCT20240623062228N1 2024, 2024, 2024, 2023, 2023; IRCT20230206057338N2 2024; IRCT20220704055367N4 2022; IRCT20220629055321N1 2022, 2023, 2021, 2024, 2020, 2022; IRCT20180218038783N8 2024; IRCT20160619028528N7 2023; IRCT20160619028528N4 2020; IRCT20160608028352N13 2023; IRCT20150119020719N6 2018; IRCT20120215009014N509 2024; IRCT20120215009014N477 2023; IRCT2017013032245N2 2017; IRCT2016081629388N1 2017, 2016, 2016, 2017, 2016; IRCT2015082013405N14 2023; IRCT2015011920719N1 2017; Hmedeh et al. 2017; Hmedeh et al. 2021; Harrison et al. 2022; Haresnape-Tyson et al. 2016; Hardy et al. 2021; Halpern et al. 2019; Gordon et al. 2020; Gelinas et al. 2023; Forget-Renaud et al. 2023; Forget-Renaud et al. 2021; Flynn et al. 2021; Filippone et al. 2024; Fawcett et al. 2021; EUCTR2020-001069-35-ES 2021; Erickson et al. 2020; Duval, Langlois, et al. 2015; Duval, Belan, Jean-Denis, Carranza-Mamane, et al. 2015; Duval, Belan, Jean-Denis, and Baillargeon 2015; Dupont et al. 2020; DRKS00017554 2019; Draper et al. 2023; Dietz De Loos et al. 2019; Dennis et al. 2023; Dennis et al. 2021; De Loos et al. 2019; CTRI/2024/10/075211 2024; CTRI/2024/03/064582 2024; CTRI/2024/01/061753 2024; CTRI/2023/04/051620 2023; CTRI/2022/03/041365 2022; CTRI/2021/12/038831 2021; CTRI/2020/10/028770 2020; CTRI/2019/08/020997 2019; CTRI/2017/06/008908 2017; Clark et al. 2000; Chizen et al. 2014; ChiCTR2300078072 2023, 2023, 2022, 2022; Callahan et al. 2024; Brule et al. 2023; Borthakur et al. 2020; Boedt et al. 2020; Boedt, Dancet, Spiessens, et al. 2023; Bivia-Roig et al. 2020; Belan et al. 2022; Belan et al. 2021; Belan, Carranza-Mamane, AinMelk, Pesant, Duval, Jean-Denis, Langlois, Lavoie, et al. 2019; Belan, Carranza-Mamane, et al. 2019, 2019; Beerendonk, Scheepers, et al. 1996; Baillargeon et al. 2022; Ansari et al. 2021; ACTRN12622001470785 2022, 2021, 2020, 2020, 2020, 2014, 2014)
Awaiting classification due to integrity concerns	(Azami et al. 2020; IRCT2017052333834N4 2017; Manteghi et al. 2021; Zhang et al. 2017; Sun and Niu 2020; NCT01892111 2013; Palomba, Falbo, et al. 2010; Palomba, Giallauria, et al. 2010; Osman et al. 2024)

Supplementary Table S9. Intervention characteristics according to the TIDieR checklist.

Authors	Why (theoretical framework)	What (lifestyle components, materials, procedures and comparator intervention)	Who (intervention provider)	How (use of technology, format)	Where (location)	When and how much (intervention duration/timing, session duration/scheduling)	Tailoring	How well (fidelity strategies, fidelity data)
Becker 2015	NR	Weight loss via diet only<br><br>Written menus, olive oil and dried fruit provided to participants<br><br>Dietary advice to consume a hypocaloric (20 kcal/kg/day) low GI and low GL diet<br><br>No lifestyle intervention given to comparator group (maintained their usual diet)	Dietitian	Face-to-face only<br><br>Individual format only	Hospital	12-week intervention<br><br>2 sessions (weeks 0 and 6; session duration NR)	NR	Reinforcement of dietary advice at 6 weeks<br><br>Food diary showed participants met targets for macronutrient composition and GL; energy intake and fibre lower than targets, but fibre intake was higher than baseline intake
Beerendonk 1996	NR	Diet only (weight loss not stated as an aim)<br><br>Materials not reported	Dietitian	Face-to-face only<br><br>Individual format only	NR	~5 to 7-week intervention commencing 10 days before the start of ovarian stimulation	NR	Fidelity strategies NR<br><br>Urinary sodium to creatinine ratio was significantly lower in intervention

		Dietary instructions to consume a low-sodium diet (10-20 mg/day)<br><br>No lifestyle intervention given to comparator group (ad-libitum dietary intake)				Session number, scheduling and duration NR		group compared to control group
Beerendonk 1999	NR	Diet only (weight loss not stated as an aim)<br><br>Materials not reported<br><br>Dietary instructions to consume a low-sodium diet (10-20 mmol/day)<br><br>No lifestyle intervention given to comparator group (ad-libitum dietary intake)	Dietitian	Face-to-face only<br><br>Individual format only	NR	~5 to 7-week intervention commencing 10 days before the start of ovarian stimulation<br><br>Session number, scheduling and duration NR	NR	Fidelity strategies NR<br><br>Urinary sodium to creatinine ratio showed 3/57 assigned to intervention group and 3/62 assigned to control group were non-compliant. 3/57 assigned to intervention group and 1/62 assigned to control group had unknown compliance
Boedt 2023	Self-determination theory	Diet and physical activity (weight loss not stated as an aim)<br><br>Mobile phone application	Health professional trained in motivational interviewing	Technology only (mobile phone application and telephone calls)	N/A	52-week intervention commencing 2-10 weeks before IVF<br><br>Up to 4 telephone	Tailored advice on diet and physical activity	Fidelity strategies NR<br><br>App-based tracking showed 91.5% complied with the program, 75% used the food module and

		Advice on diet, food literacy and physical activity<br><br>No lifestyle intervention given to comparator group (mobile application with medical treatment information only)		Couple format only		sessions (duration NR) every 3 months before pregnancy		71% of women used the physical activity module
Einarsson 2017	NR	Weight loss via diet only<br><br>Low-calorie liquid formula diet<br><br>Dietary counselling and health professional contact to consume a hypocaloric diet (880 kcal/day) followed by weight stabilisation<br><br>No lifestyle intervention given to comparator group	Dietitian	Face-to-face only<br><br>Individual format only	NR	16-week intervention (12 weeks of low-calorie diet followed by 4 weeks of weight stabilisation)<br><br>5 sessions (weeks 0, 2, 5, 8 and 12) with health professional and unspecified number of sessions with a dietitian for a period of between 2 and 5 weeks; session duration NR	Individualised weight loss counselling provided to those unable to complete the low-calorie diet treatment	Fidelity strategies NR<br><br>Fidelity data NR

Espinos 2017	NR	Weight loss via diet and physical activity<br><br>Treadmill or stationary bicycle used<br><br>Dietary advice to consume a diet with a 500-800 kcal reduction in daily energy intake and supervised aerobic exercise sessions<br><br>No lifestyle intervention given to comparator group	Dietitians for dietary supervision and trained staff member for exercise supervision	Face-to-face only<br><br>Individual format only	NR	12-week intervention commencing 12 weeks before the start of IVF<br><br>5 sessions for diet (every 15 days for 12 weeks; session duration NR) and 36 × 60 min sessions for physical activity (3 times a week for 12 weeks)	Macronutrient and energy targets tailored according to weight; exercise tailored to a moderate level according to individual condition	Re-evaluation of diet if no weight loss was observed<br><br>Mean weight loss in intervention group was 5.39 kg (6.97% of body weight)
Hanafiah 2022	NR	Diet and physical activity (weight loss not stated as an aim)<br><br>Mobile phone application for participants and training handbook for intervention providers<br><br>Counselling sessions and lifestyle challenges via application	Nurses who attended training on behaviour change communication and national nutrition and physical activity guidelines	Face-to-face and technology (mobile phone application, telephone)<br><br>Individual and group formats	Participants could choose the site	33-week intervention<br><br>3 face-to-face sessions (months 1, 2 and 6) and 3 telephone sessions (months 3, 4 and 8); session duration NR	Goal setting based on individual risk assessment	Fidelity strategies NR<br><br>Fidelity data NR

		No lifestyle intervention given to comparator group						
Hillemeier 2008	Social cognitive theory	Diet and physical activity (weight loss not stated as an aim)<br><br>Printed risk assessment reports for participants<br><br>Guided physical activity and healthy eating demonstrations<br><br>No lifestyle intervention given to comparator group	Group leaders who attended study-specific training on study procedures, intervention content and group facilitation techniques	Face-to-face and technology (telephone)<br><br>Individual and group formats	Community settings (e.g. church buildings and community centres)	12-week intervention<br><br>6 × 120-minute group face-to-face sessions (fortnightly) and 6 individual telephone sessions (fortnightly between group sessions; session duration NR)	NR	Gift cards for attending sessions; session materials and make-up sessions offered to those who miss sessions<br><br>Videotaping and coding of sessions showed 77% of content was delivered as planned
Jiskoot 2020	NR	Weight loss via diet and physical activity<br><br>Textbook for participants and intervention manual for intervention providers<br><br>CBT sessions, with additional SMS support for one intervention arm, with advice to align	Mental health professional, dietitian and physical therapist	Face-to-face only for the intervention arm without SMS; face-to-face and technology (SMS) for the intervention arm with SMS	NR	52-week intervention<br><br>20 × 150-minute sessions (weekly for first 3 months, fortnightly for months 3 to 6 and monthly for months 6 to 9)	Individualised feedback for SMS group	Therapist manual used<br><br>Fidelity data NR

		diet with the Dutch Food Guide<br><br>Brief lifestyle intervention given to comparator group (risk communication and advice to lose weight via publicly available services)		Individual and group formats				
Kiel 2018	NR	Physical activity only (weight loss not stated as an aim)<br><br>Heart rate monitors<br><br>HIIT training sessions with supervision until they were familiar with the training protocols<br><br>Brief lifestyle intervention given to comparator group (advice about physical activity)	NR	Face-to-face only<br><br>Individual format only	Hospital and local gym	13 to 15-week intervention commencing 10 weeks before the start of fertility treatment<br><br>30 × 35 to 40-minute sessions (3 times per week)	Participants who experienced difficulties walking on a treadmill used a stationary bicycle instead; exercise intensity tailored to individual heart rate	Fidelity strategies NR<br><br>Fidelity data NR
Kiel 2022	NR	Physical activity only (weight loss not stated as an aim)<br><br>Heart rate monitors, online training diary and cardiovascular	NR	Face-to-face only<br><br>Individual format only	Study centre and participants' home	52-week intervention<br><br>156 × 32 to 38-minute sessions (3	Participants who experienced difficulties walking on a treadmill used a stationary	Fidelity strategies NR<br><br>Mean session completion was 2 per week

		exercise machines (treadmill, stationary bicycle or elliptical trainer) for participants<br><br>HIIT training sessions with supervision for the first 16 weeks<br><br>Brief lifestyle intervention given to comparator group (advised on current recommendations for physical activity)				times per week)	bicycle or elliptical trainer instead; exercise intensity tailored to individual heart rate	
Koduri 2024	NR	Weight loss via diet and physical activity<br><br>Consensus guidelines for South Asian region<br><br>Dietary modification to reduce calories, refined sugars, fats and carbohydrates while increasing fibre-rich foods; advice to partake in >30 mins daily physical activity	Dietitian for dietary advice and physiotherapist for physical activity advice	Face-to-face<br><br>Individual format only	Hospital outpatient department	One-off consultation each for diet and physical activity<br><br>2 sessions (baseline, session duration NR)	Tailored based on baseline diet and physical activity	Self-reported diet (24-hour recall) and physical activity (global physical activity questionnaire) were not significantly different from baseline

		Comparator group received one-time referral to a dietitian						
LeBlanc 2021	FRAMES model and social cognitive theory	Weight loss via diet and physical activity<br><br>Pedometers provided to participants<br><br>Introductory visit and telephone counselling with advice to follow a hypocaloric DASH diet aiming for 0.2-0.4 kg/week weight loss and graded physical activity targets<br><br>Brief lifestyle intervention given to comparator group (verbal and written information on how to have a healthy pregnancy)	Health coach	Face-to-face and technology (telephone and website)<br><br>Individual format only	Telephone and web-based	104-week intervention; continued into pregnancy with same frequency and scheduling<br><br>1 × 30 to 40-min face-to-face session (week 0) and 44 × 20 to 30-min telephone sessions (weekly for first 6 months and then monthly for 18 months)	Individualised goal setting and problem-solving strategies, with energy targets personalised for individual weight loss goals	Investigators observed health coaching sessions and met with coaches to provide feedback and discuss any compliance issues; discrepancies from recommendations discussed; birthday and holiday cards given to participants<br><br>24-hour dietary recall (collected from 62% of participants) showed the intervention group significantly increased fruit intake at mid-pregnancy compared to the control group; physical activity questionnaire (completed by 66% of participants) showed the intervention group significantly

								increased vigorous activity and sports while decreasing sedentary time at mid-pregnancy compared to the control group
Lumley 2006	NR	Diet and physical activity (weight loss not stated as an aim)<br><br>Trial logbook and information card for participants<br><br>Advice on addressing individual lifestyle risk factors<br><br>No lifestyle intervention given to comparator group	Midwife	Face-to-face and technology (telephone)<br><br>Individual format only	Participants' home	One-off visit<br><br>One-off face-to-face session (session duration NR) with the provision of a telephone number to call for any questions	Individual risk factors assessed and sessions personalised based on the concerns, questions and experiences of each woman	Fidelity strategies NR<br><br>Fidelity data NR
Mohseni 2021	NR	Physical activity only (weight loss not stated as an aim)<br><br>Training package for participants<br><br>Supervised and unsupervised yoga sessions	Y7oga instructor	Face-to-face and technology (telephone)<br><br>Individual and group formats	Hospital gym and participants' home	6-week intervention commencing at the same time as fertility treatment<br><br>12 × 90-minute sessions in hospital (twice	NR	Participants received follow-up phone calls from researcher<br><br>Session completion data showed one participant did not perform yoga exercises regularly

		No lifestyle intervention given to comparator group				per week) and 30 × 90-minute sessions at home (5 times per week)		
Moran 2011	NR	Weight loss via diet and physical activity<br><br>Liquid meal replacement provided to participants<br><br>Advice to follow a hypocaloric diet (1283 kcal/day), full-body resistance training program and walking program<br><br>Brief lifestyle intervention given to comparator group (1 session with advice on diet and lifestyle factors influencing fertility)	Dietitian	Face-to-face and technology (telephone)<br><br>Individual format only	Clinic (dietary advice) and participants' home (resistance training)	7.5-week intervention (mean). Ranged from 5 to 9 weeks and commenced at the start of the period prior to the IVF cycle<br><br>2 face-to-face and 1 telephone session (scheduled fortnightly; session duration NR)	NR	Fidelity strategies NR<br><br>Fidelity data NR
Mutsaerts 2016	NR	Weight loss via diet and physical activity<br><br>Web-based food diary, physical activity diary and pedometer for	Nurses or dietitians who received study-specific training	Face-to-face and technology (telephone, email and web-based food diary)	Research sites	26-week intervention commencing 26 weeks before IVF	Individualised goal setting	Structured software program used to guide counselling, supervision of sessions by one nurse and yearly training for

		participants; software program to guide counselling for intervention providers<br><br>Advice to follow a hypocaloric diet (600 kcal/day reduction) and structured exercise program<br><br>No lifestyle intervention given to comparator group		Individual format only		6 × 30 to 60-minute face-to-face sessions (weeks 1, 3, 7, 12, 18 and 24) and 3 × 15-minute telephone sessions (weeks 5, 9 and 21)		intervention providers<br><br>Food diary showed women in the intervention arm reduced their mean energy intake by 472 kcal/day at 6 months compared to baseline; pedometer showed women increased their mean number of steps by 3231 at 6 months compared to baseline
Ng 2021	Fogg's behaviour model, social cognitive theory and transtheoretical model	Diet only (weight loss advised only for those above a healthy weight)<br><br>Smartphone application for participants<br><br>Tailored lifestyle advice based on risk factors identified in baseline questionnaire (weight, inadequate fruit intake, inadequate vegetable intake)	Mobile phone application	Technology only (mobile phone application)<br><br>Individual format only	N/A	24-week intervention<br><br>No synchronous sessions; 4 emails sent (weeks 6, 12, 18 and 24)	Emails adjusted to address individual risk factors	Fidelity strategies NR<br><br>Questionnaire completion was 87.4% at 12 weeks and 72.1% at 24 weeks

		Brief lifestyle intervention given to comparator group (advised to access a website with information on preconception health)						
Oostingh 2010	Fogg's behaviour model, social cognitive theory and transtheoretical model	Diet only (weight loss not stated as an aim)<br><br>Smartphone application for participants<br><br>Tailored lifestyle advice based on risk factors identified in questionnaire (inadequate fruit intake, inadequate vegetable intake)<br><br>Brief lifestyle intervention given to comparator group (online resources and seasonal recipes)	Mobile phone application	Technology only (mobile phone application)<br><br>Individual format only	N/A	24-week intervention; continued into pregnancy with the same frequency and scheduling<br><br>No synchronous sessions; up to 3 SMS and email messages per week sent	SMS and email messages adjusted to address individual risk factors	Fidelity strategies NR<br><br>Lifestyle risk score was decreased by a significantly greater extent in the intervention group compared to the control group; serum folate levels were significantly higher in the intervention group compared to the control group
Phelan 2023	Social cognitive theory	Weight loss via diet and physical activity	NR	Face-to-face and technology (telephone	NR	50-week intervention (mean); continued until	Additional sessions for participants who regained	Weekly supervision meeting with the intervention team; intervention team

		Pedometers for participants<br><br>Instructions to restrict energy intake (aiming to produce 10% weight loss over 16 weeks) and increase physical activity<br><br>Brief lifestyle intervention given to comparator group (general information about preconception health)		and video conferencin g)<br><br>Individual sessions only		participants became pregnant<br><br>Variable number of 30-minute sessions (weekly for first 16 weeks then fortnightly until conception, with frequency increased if weight is regained)	weight; individualised energy targets	discussed participant cases and audiotaped sessions were reviewed to promote adherence<br><br>Fidelity data NR
Rono 2018	NR	Diet and physical activity (weight loss advised only for those above a healthy weight)<br><br>Pedometers and diet diaries<br><br>Counselling based on national Finnish guidelines with an energy target of 1600-1800 kcal/day	Nurse and nutritionist	Face-to-face only<br><br>Individual and group formats	Hospital	65-week intervention (mean); continued into pregnancy<br><br>1 session every 3 months before pregnancy, plus one session per trimester during pregnancy	Adjusted to individual risk factors; modified to individual limitations (e.g. focused counselling on diet for participants with contraindicatio ns to physical activity)	Additional sessions provided to participants who were not meeting targets<br><br>3-day food diaries showed participants in the intervention group increased their mean Healthy Food Intake Index score by 1.2 points; physical activity logbook showed

		Brief lifestyle intervention given to comparator group (leaflets on healthy diet and exercise)				(session duration NR)		participants in the intervention group increased their mean leisure-time physical activity by 24 minutes
Sim 2014	NR	Weight loss via diet and physical activity<br><br>Pedometer, meal replacement products and self-monitoring diary (diet and physical activity)<br><br>Advice to follow a VLED consisting of 609 kcal/day followed by a mildly hypocaloric diet with a 598 kcal/day deficit, and graded step targets<br><br>Brief lifestyle intervention given to comparator group (advised to see GP for weight loss advice; referred to public weight loss services if BMI ≥ 35 kg m^{-2})	Fertility fellow, midwife, fertility counsellor or dietitian	Face-to-face only<br><br>Group format only	NR	12-week intervention commencing 12 weeks before the start of fertility treatment<br><br>12 sessions (weekly; session duration NR)	Individual dietary plans based on participants' initial dietary intake	Feedback on diet and physical activity diary to promote adherence<br><br>Fidelity data NR

Van Uytsel 2022	Transtheoretical model	Diet and physical activity (weight loss advised only for those above a healthy weight)<br><br>Body weight scale and mobile phone application for participants<br><br>Education on nutrition, physical activity, weight and mental wellbeing<br><br>No lifestyle intervention given to comparator group	Health coaches who received training from a nutritionist and psychologist	Face-to-face and technology (mobile phone application)<br><br>Individual format only	Hospital, private clinic, child and family organisation or participants' home, depending to participant preference	26-week interpregnancy intervention with resumption of intervention during pregnancy<br><br>4 interpregnancy sessions (weeks 6, 8, 12 and 26 postpartum) and 3 sessions during pregnancy (week <15, 20 and 35; session duration NR)	Individualised goal setting and customised advice	Coaching sessions coincide with routine visits and participants are given flexibility with study location to improve session attendance<br><br>Fidelity data NR
Wang 2023	NR	Weight loss via diet and physical activity<br><br>Mobile phone application for monitoring diet, physical activity and weight<br><br>Energy restricted diet (reduce energy intake by 500kcal/day with	Dietitian	Face-to-face and technology (mobile phone application)<br><br>Individual format only	Hospital	12-week intervention with commencing 12 weeks before IVF<br><br>One face-to-face session and regular follow-up via mobile phone application	Individualised diet based on baseline diet	Fidelity strategies NR<br><br>Daily energy intake and physical activity monitoring by dietitian showed good compliance in 94% of participants

		minimum intake of 1200kcal/day) with moderate-intensity physical activity and 10,000 steps per day, aiming to produce 5-10% weight loss<br><br>No lifestyle intervention given to comparator group				(session duration NR)		

CBT, cognitive behavioural therapy; DASH, Dietary Approaches to Stop Hypertension; FRAMES, Feedback, Responsibility, Advice, Menu of options, Empathy, and Self-Efficacy; GI, glycaemic index; GL, glycaemic load; IVF, in vitro fertilisation; N/A, not applicable; NR, not reported; SMS, short message service; VLED, very low energy diet.

Supplementary Table S10. Behaviour change techniques utilised in studies

Behaviour change techniques	Studies	Examples of how behaviour change techniques were delivered
1.1 Goal setting (behaviour)	(Hanafiah et al. 2022; Leblanc, Smith, et al. 2021; Phelan et al. 2023; Rono et al. 2018; Van Uytsel et al. 2022; Boedt, Dancet, De Neubourg, et al. 2023; Koduri et al. 2024; Wang et al. 2023)	Women set goals for the number of steps to achieve each day (Van Uytsel et al. 2022; Phelan et al. 2023)
1.2 Problem solving	(Hanafiah et al. 2022; Hillemeier et al. 2008; Jiskoot et al. 2020; Leblanc, Smith, et al. 2021; Phelan et al. 2023; Van Uytsel et al. 2022)	Health coach worked collaboratively with the participant to develop personalised problem-solving strategies (Leblanc, Smith, et al. 2021)
1.3 Goal setting (outcome)	(Hanafiah et al. 2022; Hillemeier et al. 2008; Jiskoot et al. 2020; Leblanc, Smith, et al. 2021; Mutsaerts et al. 2016; Phelan et al. 2023; Van Uytsel et al. 2022; Wang et al. 2023)	Women were advised to aim to lose 1–2 lb per week for the first 16 weeks (Phelan et al. 2023)
1.4 Action planning	(Van Uytsel et al. 2022)	Women set SMART (Specific, Measurable, Achievable, Relevant and Time specific) goals and action plans (Van Uytsel et al. 2022)
1.5 Review behaviour goal(s)	(Leblanc, Smith, et al. 2021; Mutsaerts et al. 2016; Rono et al. 2018; Boedt, Dancet, De Neubourg, et al. 2023)	Health coaches assessed women's goal progress and set new goals (Leblanc, Smith, et al. 2021)
1.7 Review outcome goal(s)	(Hanafiah et al. 2022)	Goals were revised as relevant (Hanafiah et al. 2022)
1.8 Behavioural contract	(Mutsaerts et al. 2016)	Individualised goals were embedded into a patient contract (Mutsaerts et al. 2016)
2.1 Monitoring of behaviour by others without feedback	(Hanafiah et al. 2022)	Intervention provider had access to an online tool which records women's lifestyle behaviours (Hanafiah et al. 2022)
2.2 Feedback on behaviour	(Hanafiah et al. 2022; Hillemeier et al. 2008; Jiskoot et al. 2020; Leblanc, Smith, et al. 2021; Mutsaerts et al. 2016; Ng et al. 2021; Oostingh et al. 2020; Sim et al. 2014; Van Uytsel et al. 2022; Boedt, Dancet, De Neubourg, et al. 2023; Wang et al. 2023)	Women were sent emails containing feedback on progress (Ng et al. 2021)
2.3 Self-monitoring of behaviour	(Hanafiah et al. 2022; Hillemeier et al. 2008; Jiskoot et al. 2020; Leblanc, Smith, et al. 2021; Mutsaerts et al. 2016; Phelan et al. 2023; Sim et al. 2014; Van Uytsel et al. 2022; Wang et al. 2023; Boedt, Dancet, De Neubourg, et al. 2023) [a]	Women were advised to track all dietary intake and physical activity (Leblanc, Smith, et al. 2021)

2.4 Self-monitoring of outcome(s) of behaviour	(Leblanc, Smith, et al. 2021; Phelan et al. 2023; Van Uytsel et al. 2022; Wang et al. 2023)	Women were advised to weigh themselves at least once per week (Leblanc, Smith, et al. 2021)
2.7 Feedback on outcome(s) of behaviour	(Sim et al. 2014; Wang et al. 2023)	Women were provided with feedback on anthropometric changes (Sim et al. 2014)
3.1 Social support (unspecified)	(Hillemeier et al. 2008; Jiskoot et al. 2020; Leblanc, Smith, et al. 2021; Lumley and Donohue 2006; Mutsaerts et al. 2016; Sim et al. 2014; Van Uytsel et al. 2022)	Women were encouraged to find an exercise buddy (Hillemeier et al. 2008)
3.3 Social support (emotional)	(Hanafiah et al. 2022; Boedt, Dancet, De Neubourg, et al. 2023)	Intervention provider used motivational interviewing (Hanafiah et al. 2022)
4.1 Instruction on how to perform the behaviour	(Becker et al. 2015; Einarsson et al. 2017; Espinós et al. 2017; Hanafiah et al. 2022; Hillemeier et al. 2008; Jiskoot et al. 2020; Lumley and Donohue 2006; Mohseni et al. 2021; Mutsaerts et al. 2016; Van Uytsel et al. 2022; Boedt, Dancet, De Neubourg, et al. 2023; Wang et al. 2023)	Women were provided with a list of foods which were compliant with the dietary intervention (Becker et al. 2015)
5.1 Information about health consequences	(Hillemeier et al. 2008; Mutsaerts et al. 2016)	Women were provided with information on the relationship between lifestyle behaviours and pregnancy complications (Mutsaerts et al. 2016)
5.3 Information about social and environmental consequences	(Van Uytsel et al. 2022)	Women were informed about the benefits of a healthy lifestyle (Van Uytsel et al. 2022)
6.1 Demonstration of the behaviour	(Hillemeier et al. 2008; Kiel et al. 2022; Kiel et al. 2018; Mohseni et al. 2021)	Sessions included guided physical activity and healthy eating demonstrations (Hillemeier et al. 2008)
7.1 Prompts/cues	(Mohseni et al. 2021)	Women received phone call reminders to do yoga exercises at home (Mohseni et al. 2021)
8.1 Behavioural practice/rehearsal	(Hillemeier et al. 2008; Kiel et al. 2022; Kiel et al. 2018; Mohseni et al. 2021)	Women attended yoga classes (Mohseni et al. 2021)
8.2 Behaviour substitution	(Hanafiah et al. 2022; Jiskoot et al. 2020; Leblanc, Smith, et al. 2021)	Women were advised to replace high-calorie foods with lower-calorie foods (Leblanc, Smith, et al. 2021)
8.6 Generalisation of target behaviour	(Kiel et al. 2022; Kiel et al. 2018; Mohseni et al. 2021)	After gaining familiar with the exercise protocols during supervised sessions, participants performed the exercises at home or at a local gym (Kiel et al. 2018)
8.7 Graded tasks	(Leblanc, Smith, et al. 2021; Moran et al. 2011; Phelan et al. 2023; Sim et al. 2014; Boedt, Dancet, De Neubourg, et al. 2023)	Participants were advised to gradually increase the number of steps and the frequency, duration and intensity of physical activity (Leblanc, Smith, et al. 2021)

9.1 Credible source	(Becker et al. 2015; Einarsson et al. 2017; Espinós et al. 2017; Hanafiah et al. 2022; Mutsaerts et al. 2016; Sim et al. 2014; Boedt, Dancet, De Neubourg, et al. 2023; Wang et al. 2023)	Dietary advice was given by dietitians (Becker et al. 2015)
10.8 Incentive (outcome)	(Mutsaerts et al. 2016; Wang et al. 2023)	Women were informed they could proceed to fertility treatment before the intervention was finished if they met weight loss targets before 6 months (Mutsaerts et al. 2016)
10.10 Reward (outcome)	(Mutsaerts et al. 2016; Wang et al. 2023)	Women who met weight loss targets before 6 months could proceed to fertility treatment before the intervention was finished (Mutsaerts et al. 2016)
11.2 Reduce negative emotions	(Hillemeier et al. 2008)	Intervention included stress management strategies (Hillemeier et al. 2008)
12.5 Adding objects to the environment	(Becker et al. 2015; Espinós et al. 2017; Hillemeier et al. 2008; Leblanc, Smith, et al. 2021; Moran et al. 2011; Phelan et al. 2023; Sim et al. 2014)	Meal replacement products were provided to women (Einarsson et al. 2017; Moran et al. 2011; Sim et al. 2014)
12.6 Body changes	(Hillemeier et al. 2008)	Intervention included relaxation training (Hillemeier et al. 2008)
13.2 Framing/reframin g	(Jiskoot et al. 2020; Phelan et al. 2023)	Interventions included cognitive restructuring (Jiskoot et al. 2020; Phelan et al. 2023)

[a] Behaviour change technique present in only one of the two intervention arms (lifestyle intervention with short message service) for Jiskoot 2020.

Supplementary Table S11. Results from pooled meta-analysis.

**Outcomes**	**Type of data**	**Number of studies included in meta-analysis**	**Number of participants included in meta-analysis**	**Odds ratio [95% confidence interval] for categorical, or mean difference [95% confidence interval] for continuous**	**I^2 (%)**
Primary outcomes					
Live birth	Categorical	9 (Becker et al. 2015; Einarsson et al. 2017; Espinós et al. 2017; Jiskoot et al. 2020; Moran et al. 2011; Mutsaerts et al. 2016; Sim et al. 2014; Wang et al. 2023; Boedt, Dancet, De Neubourg, et al. 2023)	1539	1.17 [0.82, 1.67]	48.73
Clinical pregnancy	Categorical	15 (Becker et al. 2015; Beerendonk et al. 1999; Beerendonk, Derkx, et al. 1996; Einarsson et al. 2017; Espinós et al. 2017; Jiskoot et al. 2020; Kiel et al. 2018; Moran et al. 2011; Mutsaerts et al. 2016; Ng et al. 2021; Oostingh et al. 2020; Sim et al. 2014; Boedt, Dancet, De Neubourg, et al. 2023; Koduri et al. 2024; Wang et al. 2023)	2500	1.06 [0.84, 1.35]	24.22
Excessive gestational weight gain	Categorical	2 (Leblanc, Smith, et al. 2021; Phelan et al. 2023)	525	1.67 [1.07, 2.59]	0.00
Metabolic syndrome	Categorical	1 (Mutsaerts et al. 2016)	577	0.52 [0.37, 0.74]	N/A

**Outcomes**	**Type of data**	**Number of studies included in meta-analysis**	**Number of participants included in meta-analysis**	**Odds ratio [95% confidence interval] for categorical, or mean difference [95% confidence interval] for continuous**	**I^2 (%)**
Weight (kg)	Continuous	12 (Becker et al. 2015; Beerendonk, Derkx, et al. 1996; Einarsson et al. 2017; Hanafiah et al. 2022; Kiel et al. 2018; Leblanc, Smith, et al. 2021; Moran et al. 2011; Mutsaerts et al. 2016; Phelan et al. 2023; Sim et al. 2014; Van Uytsel et al. 2022; Koduri et al. 2024)	2611	-3.78 [-5.65, -1.92]	95.03
BMI (kg m^{-2})	Continuous	9 (Becker et al. 2015; Einarsson et al. 2017; Hanafiah et al. 2022; Kiel et al. 2018; Leblanc, Smith, et al. 2021; Mohseni et al. 2021; Moran et al. 2011; Mutsaerts et al. 2016; Sim et al. 2014)	1506	-1.45 [-2.23, -0.67]	94.17
Waist circumference (cm)	Continuous	9 (Hanafiah et al. 2022; Kiel et al. 2018; Mohseni et al. 2021; Moran et al. 2011; Mutsaerts et al. 2016; Sim et al. 2014; Van Uytsel et al. 2022; Koduri et al. 2024; Wang et al. 2023)	2224	-2.37 [-4.25, -0.49]	85.54
Waist to hip ratio	Continuous	4 (Becker et al. 2015; Kiel et al.	630	-0.01 [-0.05, 0.02]	57.71

**Outcomes**	**Type of data**	**Number of studies included in meta-analysis**	**Number of participants included in meta-analysis**	**Odds ratio [95% confidence interval] for categorical, or mean difference [95% confidence interval] for continuous**	**I^2 (%)**
		2018; Mutsaerts et al. 2016; Koduri et al. 2024)			
Hip circumference (cm)	Continuous	5 (Becker et al. 2015; Mohseni et al. 2021; Mutsaerts et al. 2016; Koduri et al. 2024; Wang et al. 2023)	745	-2.58 [-4.54, -0.62]	66.93
Body fat percentage	Continuous	3 (Becker et al. 2015; Kiel et al. 2018; Van Uytsel et al. 2022)	1116	-1.91 [-4.36, 0.55]	77.06
Gestational weight gain (kg)	Continuous	1 (Phelan et al. 2023)	49	3.00 [-1.29, 7.29]	N/A
Fat-free mass (kg)	Continuous	1 (Kiel et al. 2018)	18	0.50 [-4.04, 5.04]	N/A
Visceral fat (cm^2)	Continuous	1 (Kiel et al. 2018)	18	-8.60 [-22.09, 4.89]	N/A
Fasting glucose (mmol/L)	Continuous	5 (Becker et al. 2015; Kiel et al. 2018; Leblanc, Smith, et al. 2021; Mutsaerts et al. 2016; Phelan et al. 2023)	712	-0.15 [-0.25, -0.04]	0.00
Fasting insulin (pmol/L)	Continuous	4 (Becker et al. 2015; Kiel et al. 2018; Leblanc, Smith, et al. 2021; Mutsaerts et al. 2016)	678	-6.00, [-16.53, 4.52]	61.96
Total cholesterol (mmol/L)	Continuous	3 (Becker et al. 2015; Kiel et al. 2018; Mutsaerts et al. 2016)	615	-0.14 [-0.27, -0.01]	0.00
LDL cholesterol (mmol/L)	Continuous	3 (Becker et al. 2015; Kiel et al. 2018; Mutsaerts et al. 2016)	615	-0.05 [-0.17, 0.07]	0.00
HDL cholesterol (mmol/L)	Continuous	3 (Becker et al. 2015; Kiel et al. 2018; Mutsaerts et al. 2016)	615	-0.02 [-0.07, 0.03]	0.01

**Outcomes**	**Type of data**	**Number of studies included in meta-analysis**	**Number of participants included in meta-analysis**	**Odds ratio [95% confidence interval] for categorical, or mean difference [95% confidence interval] for continuous**	**I^2 (%)**
Triglycerides (mmol/L)	Continuous	4 (Becker et al. 2015; Kiel et al. 2018; Leblanc, Smith, et al. 2021; Mutsaerts et al. 2016)	678	-0.22 [-0.35, -0.10]	0.00
Heart rate (beats per minute)	Continuous	2 (Kiel et al. 2018; Sim et al. 2014)	57	-0.96 [-6.13, 4.21]	0.00
CRP (mg/L)	Continuous	2 (Leblanc, Smith, et al. 2021; Mutsaerts et al. 2016)	637	-1.91 [-4.55, 0.73]	48.86
HOMA-IR	Continuous	3 (Becker et al. 2015; Leblanc, Smith, et al. 2021; Mutsaerts et al. 2016)	660	-0.32 [-0.65, 0.01]	0.00
HOMA-2IR	Continuous	2 (Becker et al. 2015; Kiel et al. 2018)	41	0.05 [-0.30, 0.39]	0.00
HbA1c (%)	Continuous	1 (Kiel et al. 2018)	18	-0.10 [-0.39, 0.19]	N/A
Systolic blood pressure (mm Hg)	Continuous	6 (Beerendonk, Derkx, et al. 1996; Kiel et al. 2018; Mohseni et al. 2021; Mutsaerts et al. 2016; Phelan et al. 2023; Sim et al. 2014)	748	-2.27 [-4.77, 0.24]	18.99
Diastolic blood pressure (mm Hg)	Continuous	6 (Beerendonk, Derkx, et al. 1996; Kiel et al. 2018; Mohseni et al. 2021; Mutsaerts et al. 2016; Phelan et al. 2023; Sim et al. 2014)	751	-1.00 [-2.91, 0.91]	54.05
Secondary outcomes					
Natural conception	Categorical	9 (Becker et al. 2015; Einarsson et al. 2017; Jiskoot et al.	1530	1.71 [0.99, 2.95]	36.50

**Outcomes**	**Type of data**	**Number of studies included in meta-analysis**	**Number of participants included in meta-analysis**	**Odds ratio [95% confidence interval] for categorical, or mean difference [95% confidence interval] for continuous**	**I^2 (%)**
		2020; Kiel et al. 2018; Mutsaerts et al. 2016; Sim et al. 2014; Wang et al. 2023; Koduri et al. 2024; Boedt, Dancet, De Neubourg, et al. 2023)			
Conception via ART	Categorical	5 (Jiskoot et al. 2020; Kiel et al. 2018; Mutsaerts et al. 2016; Sim et al. 2014; Boedt, Dancet, De Neubourg, et al. 2023)	1038	0.95 [0.64, 1.40]	12.58
Multiple pregnancy	Categorical	4 (Einarsson et al. 2017; Espinós et al. 2017; Mutsaerts et al. 2016; Boedt, Dancet, De Neubourg, et al. 2023)	1146	0.80 [0.33, 1.95]	0.00
Ovarian hyperstimulation syndrome	Categorical	3 (Espinós et al. 2017; Mutsaerts et al. 2016; Wang et al. 2023)	698	0.91 [0.09, 8.75]	36.33
Pre-term birth	Categorical	7 (Einarsson et al. 2017; Jiskoot et al. 2020; Leblanc, Smith, et al. 2021; Lumley and Donohue 2006; Mutsaerts et al. 2016; Phelan et al. 2023; Sim et al. 2014)	3230	1.10 [0.72, 1.68]	6.98
Caesarean section	Categorical	5 (Einarsson et al. 2017; Jiskoot et al. 2020; Leblanc, Smith, et al. 2021; Mutsaerts et al. 2016; Phelan et al. 2023)	1602	1.12 [0.84, 1.50]	0.00

Outcomes	Type of data	Number of studies included in meta-analysis	Number of participants included in meta-analysis	Odds ratio [95% confidence interval] for categorical, or mean difference [95% confidence interval] for continuous	I^2 (%)
Gestational hypertension	Categorical	6 (Einarsson et al. 2017; Jiskoot et al. 2020; Leblanc, Smith, et al. 2021; Mutsaerts et al. 2016; Phelan et al. 2023; Rono et al. 2018)	1828	1.05 [0.70, 1.57]	0.00
Preeclampsia	Categorical	5 (Einarsson et al. 2017; Mutsaerts et al. 2016; Phelan et al. 2023; Rono et al. 2018; Sim et al. 2014)	1370	1.01 [0.52, 1.95]	0.00
Low birth weight	Categorical	2 (Einarsson et al. 2017; Lumley and Donohue 2006)	1896	1.88 [0.99, 3.56]	0.00
Macrosomia	Categorical	2 (Einarsson et al. 2017; Phelan et al. 2023)	516	1.51 [0.23, 10.12]	27.10
Small for gestational age	Categorical	4 (Einarsson et al. 2017; Jiskoot et al. 2020; Mutsaerts et al. 2016; Phelan et al. 2023)	1276	0.90 [0.46, 1.76]	0.00
Large for gestational age	Categorical	4 (Einarsson et al. 2017; Jiskoot et al. 2020; Mutsaerts et al. 2016; Phelan et al. 2023)	1276	0.70 [0.40, 1.23]	0.00
Pregnancy loss	Categorical	8 (Einarsson et al. 2017; Espinós et al. 2017; Leblanc, Smith, et al. 2021; Mutsaerts et al. 2016; Phelan et al. 2023; Sim et al. 2014; Wang et al. 2023; Boedt, Dancet, De Neubourg, et al. 2023)	1800	1.12 [0.71, 1.78]	37.46

**Outcomes**	**Type of data**	**Number of studies included in meta-analysis**	**Number of participants included in meta-analysis**	**Odds ratio [95% confidence interval] for categorical, or mean difference [95% confidence interval] for continuous**	**I^2 (%)**
Shoulder dystocia	Categorical	1 (Mutsaerts et al. 2016)	577	0.74 [0.16, 3.33]	N/A
Total perineal rupture	Categorical	1 (Mutsaerts et al. 2016)	577	0.20 [0.02, 1.68]	N/A
Low Apgar score	Categorical	2 (Einarsson et al. 2017; Mutsaerts et al. 2016)	894	0.53 [0.14, 1.99]	0.00
Congenital anomaly	Categorical	4 (Einarsson et al. 2017; Mutsaerts et al. 2016; Rono et al. 2018; Boedt, Dancet, De Neubourg, et al. 2023)	1333	0.98 [0.06, 15.62]	0.00
Neonatal mortality	Categorical	3 (Einarsson et al. 2017; Mutsaerts et al. 2016; Boedt, Dancet, De Neubourg, et al. 2023)	1105	0.47 [0.06, 3.68]	0.00
Postpartum haemorrhage	Categorical	2 (Einarsson et al. 2017; Mutsaerts et al. 2016)	894	0.66 [0.29, 1.50]	0.00
Admission to neonatal intensive care unit	Categorical	3 (Jiskoot et al. 2020; Mutsaerts et al. 2016; Phelan et al. 2023)	959	1.04 [0.56, 1.93]	0.00
Gestational diabetes mellitus	Categorical	7 (Einarsson et al. 2017; Jiskoot et al. 2020; Leblanc, Smith, et al. 2021; Mutsaerts et al. 2016; Phelan et al. 2023; Rono et al. 2018; Sim et al. 2014)	1879	0.88 [0.58, 1.33]	36.43
Birth weight (g)	Continuous	7 (Einarsson et al. 2017; Jiskoot et al. 2020; Leblanc, Smith, et al. 2021; Lumley and Donohue 2006; Mutsaerts et al. 2016; Rono et al. 2018; Boedt,	1607	-59.07 [-105.34, -12.81]	19.12

**Outcomes**	**Type of data**	**Number of studies included in meta-analysis**	**Number of participants included in meta-analysis**	**Odds ratio [95% confidence interval] for categorical, or mean difference [95% confidence interval] for continuous**	**I^2 (%)**
		Dancet, De Neubourg, et al. 2023)			
Testosterone (nmol/L)	Continuous	2 (Becker et al. 2015; Kiel et al. 2018)	41	0.00 [-0.02, 0.02]	0.00
Prolactin (μg/L)	Continuous	2 (Becker et al. 2015; Kiel et al. 2018)	41	0.06 [-2.07, 2.18]	0.00
Sex hormone binding globulin (nmol/L)	Continuous	2 (Becker et al. 2015; Kiel et al. 2018)	41	3.31 [-8.13, 14.76]	33.99
Free androgen index (%)	Continuous	1 (Kiel et al. 2018)	18	0.10 [-1.50, 1.70]	N/A
Leptin (ng/L)	Continuous	1 (Becker et al. 2015)	23	-21.13 [-40.38, -1.88]	N/A
Ghrelin (pg/mL)	Continuous	1 (Becker et al. 2015)	23	10.96 [-14.18, 36.10]	N/A
Gestational age at delivery (weeks)	Continuous	5 (Einarsson et al. 2017; Jiskoot et al. 2020; Lumley and Donohue 2006; Mutsaerts et al. 2016; Boedt, Dancet, De Neubourg, et al. 2023)	1301	-0.01 [-0.45, 0.42]	68.60

BMI, body mass index; HbA1c, haemoglobin A1c; HDL, high density lipoprotein; HOMA-IR, Homeostatic Model Assessment for Insulin Resistance; LDL, low density lipoprotein.

Supplementary Table S12. Summary of findings table.

Certainty assessment							n participants		Effect		Certainty	Importance
n studies	Study design	Risk of bias	Inconsistency	Indirectness	Imprecision	Other considerations	lifestyle interventions	standard care or no intervention	Relative (95% CI)	Absolute (95% CI)		
Live birth												
9	randomised trials	not serious	not serious	not serious	serious [a]	none	307/804 (38.2%)	285/735 (38.8%)	OR 1.17 (0.82 to 1.67)	38 more per 1,000 (from 46 fewer to 126 more)	⨁⨁⨁◯ Moderate [a]	CRITICAL
Clinical pregnancy												
15	randomised trials	not serious	not serious	not serious	serious [a]	none	746/1698 (43.9%)	721/1602 (45.0%)	OR 1.06 (0.84 to 1.35)	14 more per 1,000 (from 43 fewer to 75 more)	⨁⨁⨁◯ Moderate [a]	CRITICAL
Weight												
12	randomised trials	not serious	serious [b]	not serious	not serious	publication bias strongly suspected [c]	1325	1286	-	MD 3.78 kg lower (5.65 lower to 1.92 lower)	⨁⨁◯◯ Low [b,c]	IMPORTANT
Waist circumference												
9	randomised trials	not serious	serious [b]	not serious	not serious	none	1118	1106	-	MD 2.37 cm lower (4.25	⨁⨁⨁◯ Moderate [b]	IMPORTANT

										lower to 0.49 lower)		
Fasting blood glucose												
5	randomised trials	not serious	not serious	not serious	serious [a]	none	365	347	-	MD 0.15 mM lower (0.25 lower to 0.04 lower)	⨁⨁⨁◯ Moderate	IMPORTANT

CI, confidence interval; MD, mean difference; OR, odds ratio. [a] downgraded 1 level due to serious concerns about imprecision. [b] downgraded 1 level due to serious concerns about inconsistency. [c] downgraded 1 level due to concerns about publication bias.

Supplementary Table S13. Results for sensitivity analysis restricted to women with obesity.

**Outcomes**	**Type of data**	**Number of studies included in sensitivity analysis**	**Number of participants included in sensitivity analysis**	**Odds ratio [95% confidence interval] for categorical, or mean difference [95% confidence interval] for continuous**	**I^2 (%)**
Primary outcomes [a]					
Live birth	Categorical	3 (Einarsson et al. 2017; Espinós et al. 2017; Sim et al. 2014)	397	1.98 [0.77, 5.08]	59.30
Clinical pregnancy	Categorical	3 (Einarsson et al. 2017; Espinós et al. 2017; Sim et al. 2014)	397	2.12 [0.82, 5.45]	60.70
Weight (kg)	Continuous	2 (Einarsson et al. 2017; Sim et al. 2014)	348	-7.78 [-12.96, -2.60]	92.58
BMI (kg m^{-2})	Continuous	2 (Einarsson et al. 2017; Sim et al. 2014)	348	-1.68 [-2.54, -0.82]	83.31
Waist circumference (cm)	Continuous	1 (Sim et al. 2014)	43	-1.35 [-2.02, -0.69]	N/A
Heart rate (beats per minute)	Continuous	1 (Sim et al. 2014)	39	-0.13 [-0.75, 0.49]	N/A
Systolic blood pressure (mm Hg)	Continuous	1 (Sim et al. 2014)	39	-0.23 [-0.86, 0.39]	N/A
Diastolic blood pressure (mm Hg)	Continuous	1 (Sim et al. 2014)	39	-0.43 [-0.19, 1.06]	N/A

[a] No observations for excessive gestational weight gain, metabolic syndrome, waist to hip ratio, hip circumference, body fat percentage, gestational weight gain, fat-free mass, visceral fat, fasting glucose, fasting insulin, total cholesterol, low density lipoprotein cholesterol, high density lipoprotein cholesterol, triglycerides, C-reactive protein, haemoglobin A1c, homeostatic model of insulin resistance or homeostatic model of insulin resistance 2.

Supplementary Figure S1. Contour-enhanced funnel plot for clinical pregnancy

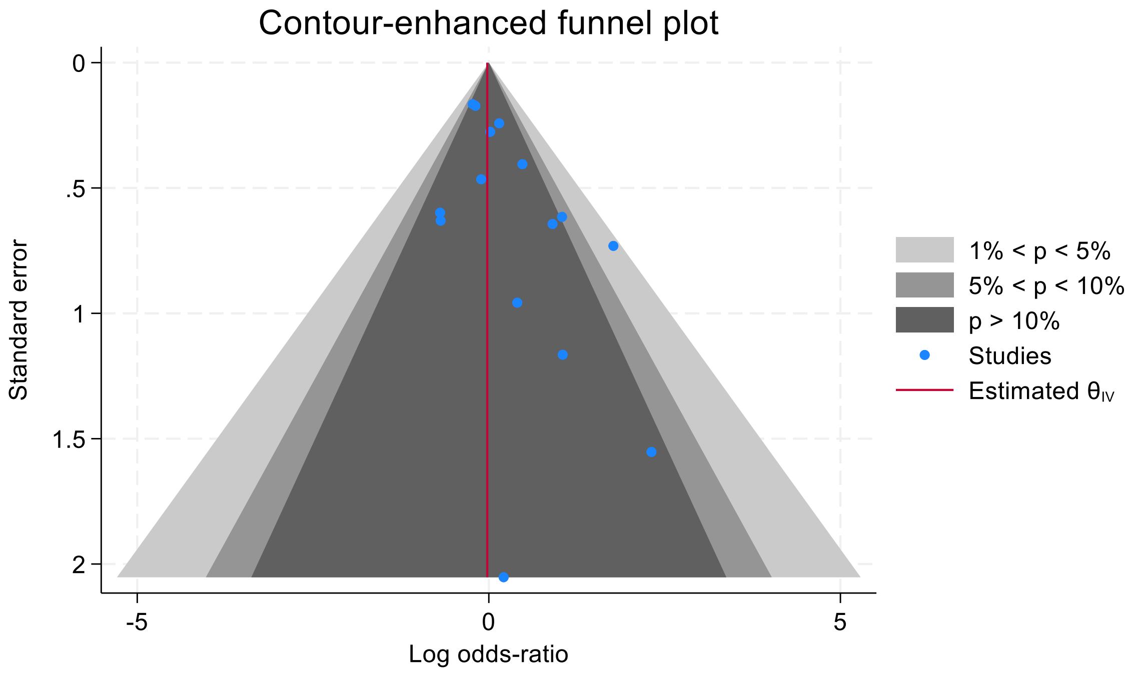

Supplementary Figure S2. Contour-enhanced funnel plot for weight

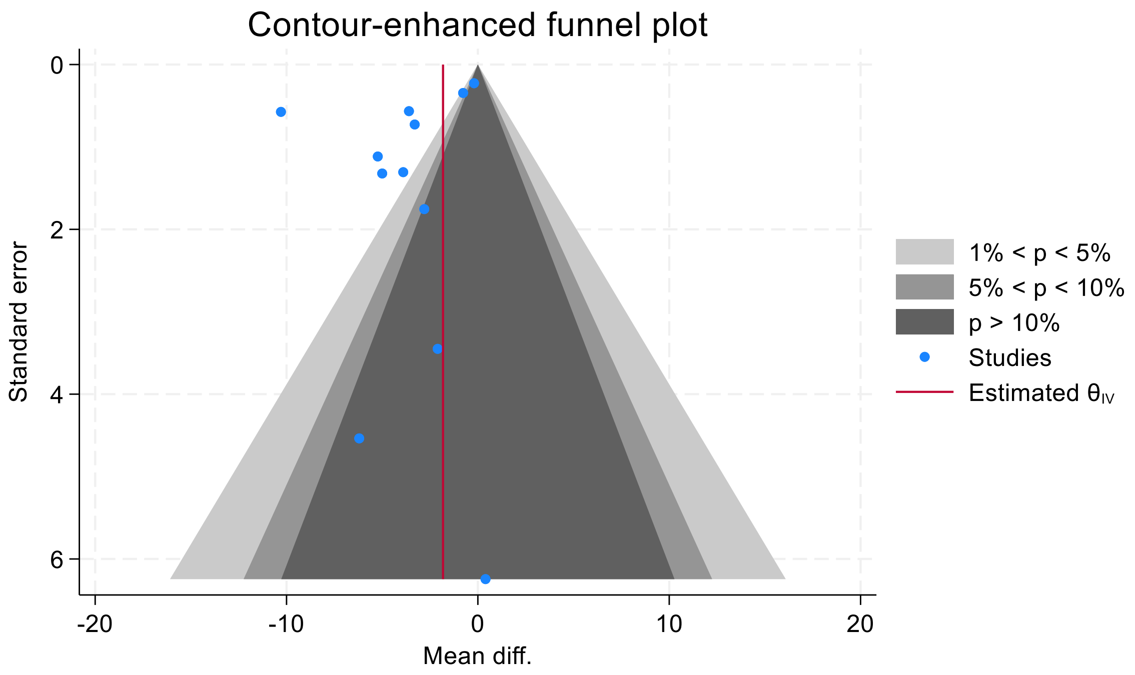

References

Abdollahi L, Mirghafourvand M, Babapour JK, and Mohammadi M. Effectiveness of cognitive-behavioral therapy (CBT) in improving the quality of life and psychological fatigue in women with polycystic ovarian syndrome: a randomized controlled clinical trial. *Journal of Psychosomatic Obstetrics and Gynecology* 2019: **40**; 283-293.

ACTRN12614000517673. Effect of progressive resistance training in women with polycystic ovary syndrome. A feasibility study. *A randomised control trial comparing the benefits of progressive resistance training and/or usual care on health status in women with polycystic ovary syndrome* 2014.

ACTRN12614001160628. Pregnancy Outcomes after Pre-pregnancy weight loss in obese women (POP Study). *Does substantial pre-conception weight loss, achieved using a 12-week program of a Very Low Energy Diet, improve pregnancy outcomes compared to modest weight loss achieved using a diet and exercise program in obese women?* 2014.

ACTRN12620001238965. Can a pre-conception weight loss program improve maternal and infant outcomes for women with overweight or obesity? A pragmatic randomised controlled trial (RCT). 2020.

ACTRN12620001053910. Optimising Health in Women across Pre-Pregnancy, Pregnancy & Post-Birth. *A randomised, healthy lifestyle intervention to optimise weight and weight related behaviours across preconception, pregnancy and postpartum: The OptimalMe Program* 2020.

ACTRN12620000597998. PRE-BABE: Pre-conception weight loss for women above a healthy weight: a pilot randomized controlled trial. *Feasibility and acceptability of an online pre-conception weight loss program for women with overweight and obesity? A pilot randomised controlled trial* 2020.

ACTRN12621000128897. The effect of pre-pregnancy dietary advice and regular exercise to promote weight loss in overweight or obese women on pregnancy outcomes: the BEGIN BETTER randomised trial. 2021.

ACTRN12622001470785. The effect of pre-pregnancy dietary advice and regular exercise to promote health in women with BMI between 18.5 and 24.9kg/m2, on pregnancy outcomes: the Begin Better 2.0 randomised trial. 2022.

Alibeigi Z, Jafari-Dehkordi E, Kheiri S, Nemati M, Mohammadi-Farsani G, and Tansaz M. Auswirkungen einer auf traditioneller Medizin basierenden Ernahrung und Lebensfuhrung auf die Infertilitatsbehandlung bei Frauen, die sich Masnahmen zur assistierten Reproduktion unterziehen: eine randomisierte kontrollierte Studie, The Impact of Traditional Medicine-Based Lifestyle and Diet on Infertility Treatment in Women Undergoing Assisted Reproduction: A Randomized Controlled Trial. *Complementary medicine research* 2020: **27**; 230-241.

Alibeigi Z, Jafari-Dehkordi E, Kheiri S, Nemati M, Mohammadi-Farsani G, and Tansaz M. The Impact of Traditional Medicine-Based Lifestyle and Diet on Infertility Treatment in Women Undergoing Assisted Reproduction: A Randomized Controlled Trial. *Auswirkungen einer auf traditioneller Medizin basierenden Ernahrung und Lebensfuhrung auf die Infertilitatsbehandlung bei Frauen, die sich Masnahmen zur assistierten Reproduktion unterziehen: eine randomisierte kontrollierte Studie.* 2020: **27**; 230-241.

Ansari F, Hamzehgardeshi Z, Elyasi F, Moosazadeh M, and Ahmadi I. The effect of online motivational interviewing on stress management in infertile women with PCOS: A randomized clinical trial. *European Psychiatry* 2021: **64**; S761.

Arentz S, Smith C, Abbott J, and Bensoussan A. Herbal medicine plus lifestyle for overweight women with polycystic ovary syndrome: A randomised control trial. *Australian Journal of Herbal and Naturopathic Medicine* 2019: **31**; 38.

Azami S, Nourizadeh R, Mehrabi E, Poursharifi H, and Farshbaf-Khalili A. Effect of motivational interviewing on dietary intake and weight changes among preconception women with overweight and obesity: A randomized controlled trial. *Crescent Journal of Medical and Biological Sciences* 2020: **7**; 260-266.

Aziz DAAE, Oqeel FM, Labib MM, and Yousef AM. Effect of aerobic exercise on inflammation and sex hormones in obese polycystic ovarian syndrome women: A randomized controlled study. *Fizjoterapia Polska* 2021: **21**; 204-209.

Aziz MV, Vakilian K, Khorsandi M, and Ranjbaran M. Preconception Care: Intention to Action-An Intervention Based on the Planned Behavior Theory for Maternal and Neonatal Health: A Randomized Clinical Trial. *Current Women's Health Reviews* 2023: **19**; e300322202811.

Baillargeon JP, Belan M, Jean-Denis F, Langlois MF, Morisset AS, and St-Laurent A. An interdisciplinary intervention improves lifestyle behaviours in women living with obesity and infertility: a randomized controlled trial. *Journal of the Endocrine Society* 2022: **6**; A674.

Barquiel B, Calvo M, Moreno-Dominguez O, Martinez-Sanchez N, Muner M, Bedate MF, Delgado M, Lopez S, Hillman N, Gonzalez N*, et al.* The PREDG study: a randomised controlled trial testing whether an educational intervention can prevent gestational weight gain in women with obesity. *Clinical nutrition ESPEN* 2023: **57**; 266.

Bastani F, Hashemi S, Bastani N, and Haghani H. Impact of preconception health education on health locus of control and self-efficacy in women. *Eastern Mediterranean health journal = La revue de sante de la Mediterranee orientale = al-Majallah al-sihhiyah li-sharq al-mutawassit* 2010: **16**; 396-401.

Becker GF, Passos EP, and Moulin CC. Short-term effects of a hypocaloric diet with low glycemic index and low glycemic load on body adiposity, metabolic variables, ghrelin, leptin, and pregnancy rate in overweight and obese infertile women: a randomized controlled trial. *Am J Clin Nutr* 2015: **102**; 1365-1372.

Beena MR and Thomas K. Outcome of Interventional Programme on Quality of Life of Infertile Women with Polycystic Ovarian Syndrome. *International Journal of Nursing Education* 2016: **8**; 27-33.

Beerendonk C, Hendriks J, Scheepers H, Braat D, Merkus J, Oostdam B, and Van Dop P. The influence of dietary sodium restriction on anxiety levels during an in vitro fertilization procedure. *Journal of Psychosomatic Obstetrics & Gynecology* 1999: **20**; 97-103.

Beerendonk CCM, Derkx FHM, Schellekens APM, Hop WC, and Van Dop PA. The influence of dietary sodium restriction on renal and ovarian renin and prorenin production during ovarian stimulation. *Human Reproduction* 1996: **11**; 956-961.

Beerendonk CMM, Scheepers HCJ, Ooststdam EMM, and Van Dop PA. Anxiety during an IVF procedure the influence of dietary sodium restrictions, several infertility characteristics and early pregnancy. *Human reproduction (Oxford, England)* 1996; 162.

Belan M, Carranza-Mamane B, AinMelk Y, Pesant MH, Duval K, Jean-Denis F, Langlois MF, and Baillargeon JP. Lifestyle modifications in male partners of subfertile couples in which the spouse is obese improves the chances of the couple to conceive. *Fertility and sterility* 2019: **112**; e213.

Belan M, Carranza-Mamane B, Ainmelk Y, Pesant MH, Duval K, Jean-Denis F, Langlois MF, and Baillargeon JP. A Lifestyle Program Targeting Women with Obesity and Infertility Improves Their Fertility: A Randomized Controlled Trial. *Journal of the Endocrine Society* 2019: **3**.

Belan M, Carranza-Mamane B, AinMelk Y, Pesant MH, Duval K, Jean-Denis F, Langlois MF, Lavoie H, Waddell G, and Baillargeon JP. A lifestyle intervention targeting women with obesity and infertility improves their fertility outcomes, especially in women with PCOS: a randomized controlled trial. *Fertility and sterility* 2019: **112**; e40.

Belan M, Carranza-Mamane B, Melk YA, Pesant MH, Jean-Denis F, Langlois MF, Poder TG, and Baillargeon JP. Cost-Effectiveness Analysis of an Interdisciplinary Lifestyle Intervention Targeting Women With Obesity and Infertility in Comparison to Usual Care. *Journal of the Endocrine Society* 2021: **5**; A730.

Belan M, Gelinas M, Carranza-Mamane B, Langlois M-F, Morisset A-S, Ruchat S-M, Lavoie K, Adamo K, Poder T, Gallagher F*, et al.* Protocol of the Fit-For-Fertility study: a multicentre randomised controlled trial assessing a lifestyle programme targeting women with obesity and infertility. *BMJ Open* 2022: **12**; e061554.

Benham JL, Booth JE, Corenblum B, Doucette S, Friedenreich CM, Rabi DM, and Sigal RJ. Exercise training and reproductive outcomes in women with polycystic ovary syndrome: A pilot randomized controlled trial. *Clinical endocrinology* 2021: **95**; 332-343.

Bivia-Roig G, Blasco-Sanz R, Boldo-Roda A, Vara MD, Escriva-Martinez T, Herrero R, La Rosa VL, Banos RM, and Lison JF. Efficacy of an Internet-Based Intervention to Promote a Healthy Lifestyle on the Reproductive Parameters of Overweight and Obese Women: Study Protocol for a Randomised Controlled Trial. *International journal of environmental research and public health* 2020: **17**.

Boedt T, Dancet E, De Neubourg D, Vereeck S, Jan S, Van Der Gucht K, Van Calster B, Spiessens C, Lie Fong S, and Matthys C. A blended preconception lifestyle programme for couples undergoing IVF: lessons learned from a multicentre randomized controlled trial. *Human Reproduction Open* 2023: **2023**.

Boedt T, Dancet E, Spiessens C, Lie Fong S, and Matthys C. Effect Of A Mobile Preconception Lifestyle Programme In Couples Undergoing In Vitro Fertilisation: a Multicentre Randomised Controlled Trial. *Clinical nutrition ESPEN* 2023: **54**; 571.

Boedt T, Voorend R, Derboven J, Dancet E, Spiessens C, and Matthys C. Development of a food literacy intervention for couples trying to conceive. *Proceedings of the Nutrition Society* 2020: **79**.

Borengasser SJ, Baker PR, 2nd, Kerns ME, Miller LV, Palacios AP, Kemp JF, Westcott JE, Morrison SD, Hernandez TL, Garces A*, et al.* Preconception Micronutrient Supplementation Reduced Circulating Branched Chain Amino Acids at 12 Weeks Gestation in an Open Trial of Guatemalan Women Who Are Overweight or Obese. *Nutrients* 2018: **10**.

Borthakur D, Kumar R, and Dada R. Yoga: A Natural Solution to Decrease Disease Burden in Children of MTHFR Deficient Parents. *La Clinica terapeutica* 2023: **174**; 28-32.

Borthakur D, Rana D, Chaurasia P, and Dada R. Yoga based lifestyle intervention and its impact on depression, quality of life and cellular aging in infertile couples. *Andrology* 2020: **8**; 38-39.

Brammall BR, Garad RM, Teede HJ, Baker SE, and Harrison CL. OptimalMe Program: a Mixed Method Investigation into the Engagement and Acceptability of a Preconception Digital Health Lifestyle Intervention with Individual Coaching for Women’s Health and Behaviour Change. *Nutrients* 2024: **16**.

Brammall BR, Harrison C, Garad R, and Teede H. Optimising health in preconception, pregnancy and postpartum (HiPPP): the OptimalMe program. Baseline preconception health and lifestyle behaviours; And postintervention behaviour change and engagement outcomes. A mixed-method study. *Obesity reviews* 2022: **23**.

Brule K, Thibodeau A, Maillet D, Belan M, Jean-Denis F, Pesant MH, Carranza-Mamane B, and Baillargeon JP. A 6-month Lifestyle Intervention Program Improves Quality Of Life And Motivation In Women With Obesity And Infertility. *Journal of the Endocrine Society* 2023: **7**; A861.

Callahan NK, Domar AD, Bleess JL, and Ainsworth AJ. MIND-BODY INTERVENTIONS PROGRAM IMPROVES DEPRESSION, ANXIETY AND STRESS SYMPTOMS IN PATIENTS UNDERGOING FERTILITY TREATMENT. *Fertility and sterility* 2024: **122**; e21

EP-e22.

Carmichael SL, Mehta K, Srikantiah S, Mahapatra T, Chaudhuri I, Balakrishnan R, Chaturvedi S, Raheel H, Borkum E, Trehan S*, et al.* Use of mobile technology by frontline health workers to promote reproductive, maternal, newborn and child health and nutrition: a cluster randomized controlled Trial in Bihar, India. *Journal of global health* 2019: **9**; 1-15.

Cena ER, Joy AB, Heneman K, Espinosa-Hall G, Garcia L, Schneider C, Wooten Swanson PC, Hudes M, and Zidenberg-Cherr S. Learner-centered nutrition education improves folate intake and food-related behaviors in nonpregnant, low-income women of childbearing age. *Journal of the American Dietetic Association* 2008: **108**; 1627-1635.

ChiCTR2200064621. Influence of professional weight management or drug therapy on live birth rate of IVF/ICSI in overweight and obese patients with polycystic ovary syndrome: a multicenter RCT clinical study. *Effects of professional weight management on IVF/ICSI live birth rates in overweight and obese patients with polycystic ovary syndrome* 2022.

ChiCTR2200057114. Study on long-term management model of multi-disciplinary intervention in polycystic ovary syndrome. 2022.

ChiCTR2300078072. Application of a shared outpatient service led by specialized nurses in obese PCOS infertility patients. 2023.
ChiCTR2300075704. Nutritional intervention for overweight and obese women with infertility. 2023.
Chizen DR, Serrao S, Rooke J, McBreairty L, Pierson RA, Chilibeck P, and Zello G. The "Pulse" diet & PCOS. *Fertility and sterility* 2014: **102**; e267.
Clark AM, Roberts B, Galletly C, Tomlinson L, and Norman RJ. Maximizing weight loss in the overweight infertile patient - a prospective randomized controlled trial. *Human reproduction (Oxford, England)* 2000: **15**; 65.
Clark AM, Thornley B, Tomlinson L, Galletley C, and Norman RJ. Weight loss in obese infertile women results in improvement in reproductive outcome for all forms of fertility treatment. *Human Reproduction* 1998: **13**; 1502-1505.
CTRI/2017/06/008908. A study on effect of Integrated Nutrition, WASH, Care and Support Interventions during the Pre-pregnancy, Pregnancy and Early Childhood on linear growth of children. *Improving Linear Growth of Children in Low Resource Settings through Integrated Nutrition, WASH, Care and Support Interventions during the Pre- and Peri-conceptional Period, Pregnancy and Early Childhood - A Randomized Controlled Trial* 2017.
CTRI/2019/08/020997. Effect of lifestyle modification and education on information needs and satisfaction level of infertile couples. *Impact of lifestyle modification and educational interventions on the information needs and satisfaction level of infertile couples reporting at infertility clinics.* 2019.
CTRI/2020/10/028770. Effect of delivery of an Integrated intervention package during Pre-pregnancy, Pregnancy and Early Childhood on infant growth markers in the first 6 months of life. *Impact of an integrated intervention package during preconception, pregnancy, and early childhood on biomarkers of infant growth in the first 6 months of life: A Sub-study in WINGS* 2020.
CTRI/2021/12/038831. Feasibility of diet and exercise as a treatment option for women with Polycystic ovarian syndrome who desire fertility: a preliminary study. *Feasibility of lifestyle intervention in PCOS women who desire fertility: a pilot randomized controlled trial. - Nil* 2021.
CTRI/2022/03/041365. Study of PCOS and to know the effect of Shatapushpaa Churna and Yoga therapy. *Clinico-etiopathological and molecular genetic association study of Polycystic Ovarian Syndrome & its management by the Shatapushpaa Churna and Yoga therapy* 2022.
CTRI/2023/04/051620. BENEFITS OF PERSONALISED LIFESTYLE IN PCOS WOMEN WITH OBESITY WHO ARE KEEN ON PREGNANCY. *EFFECTIVENESS OF AN INDIVIDUALIZED LIFESTYLE INTERVENTION IN PCOS WOMEN WHO WISH TO CONCEIVE: a MULTI - CENTERED RANDOMIZED CONTROLLED TRIAL. - IPOS Trial* 2023.
CTRI/2024/01/061753. Efficacy of mind-body therapy on couples going through Frozen Embryo Transfer. *Efficacy of mind body augmentation therapy on FET couples - NIL* 2024.
CTRI/2024/03/064582. Effect of community-based pre-conception care package on maternal and child health outcomes. *Evaluating the effect of community-based pre-conception care package through trained nurses on pre-conception health, maternal and child health outcomes: a stepped-wedge cluster randomized implementation trial. - EPIC* 2024.
CTRI/2024/10/075211. Effect of Counselling on depression in infertility couples. *The Effect of psychosocial counselling on Depression and Fertility Quality of life in couples embarking on infertility treatment A Randomized Controlled trial - NIL* 2024.
Dashti S, Abdul Hamid H, Mohamad Saini S, Tusimin M, Ismail M, Jafarzadeh Esfehani A, Ching SM, Lee KW, Ismail N, Wong JL*, et al.* A randomised controlled trial on the effects of a structural education module among women with polycystic ovarian syndrome on nutrition and physical activity changes. *BMC women's health* 2022: **22**.
De Loos AD, Timman R, Jiskoot G, Beerthuizen A, Busschbach J, and Laven J. Favorable changes in phenotype expression and androgens in women with pcos due to weight loss in a three-component lifestyle intervention program. *Reproductive Sciences* 2019: **26**; 192A.
Dennis C-L, Marini F, Dick JA, Atkinson S, Barrett J, Bell R, Berard A, Berger H, Brown HK, Constantin E*, et al.* Protocol for a randomised trial evaluating a preconception-early

childhood telephone-based intervention with tailored e-health resources for women and their partners to optimise growth and development among children in Canada: a Healthy Life Trajectory Initiative (HeLTI Canada). *BMJ Open* 2021: **11**; e046311.
Dennis C-L, Marini F, Prioreschi A, Dol J, Birken C, and Bell RC. The Canadian Healthy Life Trajectories Initiative (HeLTI) Trial: a study protocol for monitoring fidelity of a preconception-lifestyle behaviour intervention. *Trials* 2023: **24**; 262.
Dietz de Loos A, Jiskoot G, van den Berg-Emons R, Louwers Y, Beerthuizen A, van Busschbach J, and Laven J. The Effect of Tailored Short Message Service (SMS) on Physical Activity: Results from a Three-Component Randomized Controlled Lifestyle Intervention in Women with PCOS. *Journal of clinical medicine* 2023: **12**.
Dietz De Loos A, Timman R, Jiskoot G, Beerthuizen A, Van Busschbach J, and Laven J. Favorable changes in characteristics, phenotype and androgens as result of weight loss in a randomised controlled three-component lifestyle intervention in women with PCOS. *Human Reproduction* 2019: **34**.
Dokras A, Sarwer DB, Allison KC, Milman L, Kris-Etherton PM, Kunselman AR, Stetter CM, Williams NI, Gnatuk CL, Estes SJ*, et al.* Weight Loss and Lowering Androgens Predict Improvements in Health-Related Quality of Life in Women With PCOS. *The Journal of clinical endocrinology and metabolism* 2016: **101**; 2966-2974.
Doss J. Reducing pregnancy risk by motivating overweight and obese women to make preconception changes in diet and physical activity behavior: a pilot study. *Dissertation/ thesis* 2017; 1.
Draper CE, Thwala N, Slemming W, Lye SJ, and Norris SA. Development, Implementation, and Process Evaluation of Bukhali: An Intervention from Preconception to Early Childhood. *Global implementation research and applications* 2023: **3**; 31-43.
DRKS00017554. The effect of counselling on preconceptional care knowledge, attitude and lifestyle behaviors in Turkish women: a randomized controlled trial. *The effect of counselling on preconceptional care knowledge, attitude and lifestyle behaviors in Turkish women: a randomized controlled trial - pcee* 2019.
Dupont C, Aegerter P, Foucaut A-M, Reyre A, Lhuissier FJ, Bourgain M, Chabbert-Buffet N, Cedrin-Durnerin I, Selleret L, Cosson E*, et al.* Effectiveness of a therapeutic multiple-lifestyle intervention taking into account the periconceptional environment in the management of infertile couples: study design of a randomized controlled trial - the PEPCI study. *BMC pregnancy and childbirth* 2020: **20**; 322.
Duval K, Belan M, Jean-Denis F, and Baillargeon J. An interdisciplinary lifestyle intervention improves clinically relevant fertility outcomes in obese infertile women-preliminary results. *Fertility and sterility* 2015: **104**; e97.
Duval K, Belan M, Jean-Denis F, Carranza-Mamane B, Pesant M-H, Langlois M-F, Hivert M-F, Lavoie HB, and Baillargeon J-P. An Interdisciplinary Lifestyle Intervention Improves Clinically Relevant Fertility Outcomes in Obese Infertile Women – Preliminary Results of a Randomized Controlled Trial. *Canadian journal of diabetes* 2015: **39**; 532-533.
Duval K, Langlois M-F, Carranza-Mamane B, Pesant M-H, Hivert M-F, Poder TG, Lavoie HB, Ainmelk Y, St-Cyr Tribble D, Laredo S*, et al.* The Obesity-Fertility Protocol: a randomized controlled trial assessing clinical outcomes and costs of a transferable interdisciplinary lifestyle intervention, before and during pregnancy, in obese infertile women. *BMC obesity* 2015: **2**; 47.
Einarsson S, Bergh C, Friberg B, Pinborg A, Klajnbard A, Karlström P-O, Kluge L, Larsson I, Loft A, Mikkelsen-Englund A-L*, et al.* Weight reduction intervention for obese infertile women prior to IVF: a randomized controlled trial. *Human Reproduction* 2017: **32**; 1621-1630.
Erickson ML, Mey JT, Axelrod CL, Paul D, Gordesky L, Russell K, Barkoukis H, O'Tierney-Ginn P, Fielding RA, Kirwan JP*, et al.* Rationale and study design for lifestyle intervention in preparation for pregnancy (LIPP): A randomized controlled trial. *Contemporary clinical trials* 2020: **94**; 106024.
Espinós JJ, Polo A, Sánchez-Hernández J, Bordas R, Pares P, Martínez O, and Calaf J. Weight decrease improves live birth rates in obese women undergoing IVF: a pilot study. *Reproductive BioMedicine Online* 2017: **35**; 417-424.

EUCTR2020-001069-35-ES. Impact of Exercise and Mediterranean diet vs Aspirin on live-birth rate and cardiovascular programming in In Vitro Fertilization (MEDITATE-IVF). *Impact of Exercise and Mediterranean diet vs Aspirin on live-birth rate and cardiovascular programming in In Vitro Fertilization (MEDITATE-IVF): a Randomized Study. - MEDITATE-IVF* 2021.

Fawcett K, Martinez A, Crimmins M, Sims C, Borsheim E, and Andres A. Effect of a dietary and exercise intervention in women with overweight and obesity undergoing fertility treatments: protocol for a randomized controlled trial. *BMC nutrition* 2021: **7**; 51.

Filippone M, Lafleche CRD, Belan M, Jean-Denis F, La Voie H, Pesant MH, Mamane BC, and Baillargeon JP. Subgroup Analyses of a Randomized Controlled Trial Evaluating the Effects of a Lifestyle Intervention on Fertility Outcomes in Women With Obesity and Infertility. *Canadian journal of diabetes* 2024: **48**; S38.

Flynn AC, Pryke E, Wadhera M, Poston L, and White SL. A preconception intervention targeted at women with modifiable risk factors before pregnancy to improve outcomes; protocol for the Get Ready! feasibility trial. *Pilot and feasibility studies* 2021: **7**; 86.

Forget-Renaud A, Belan M, Jean-Denis F, and Baillargeon JP. An Interdisciplinary Program Promoting the Adoption of a Healthy Lifestyle Increases Insulin Sensitivity in Women With Obesity and Infertility. *Canadian journal of diabetes* 2021: **45**; S17-S18.

Forget-Renaud A, Belan M, Jean-Denis F, Pesant MH, and Baillargeon JP. An Interdisciplinary Program Promoting The Adoption Of A Healthy Lifestyle Increases Insulin Sensitivity In Women With Obesity And Infertility. *Journal of the Endocrine Society* 2023: **7**; A860.

Gangachin SE, Bayrami R, Rahimi B, and Masudi S. Smartphone-based educational and counseling interventions for women with high body mass index at Urmia's health centers. *BMC women's health* 2024: **24**; 8.

Gelinas M, Belan M, Jean-Denis F, Pesant MH, Langlois MF, Carranza-Mamane B, and Baillargeon JP. Identifying Determinants of Change In Physical Activity In Women With Obesity and Infertility Following The Obesity-Fertility Program. *Journal of the Endocrine Society* 2023: **7**; A51.

Ghasemi Yngyknd S, Mohammad-Alizadeh-Charandabi S, Babapour J, and Mirghafourvand M. The effect of counselling on preconception lifestyle and awareness in Iranian women contemplating pregnancy: a randomized control trial. *The journal of maternal-fetal & neonatal medicine : the official journal of the European Association of Perinatal Medicine, the Federation of Asia and Oceania Perinatal Societies, the International Society of Perinatal Obstetricians* 2018: **31**; 2538-2544.

Gootjes DV, van Dijk MR, Koster MP, Willemsen SP, Steegers EA, and Steegers-Theunissen RP. Neighborhood Deprivation and the Effectiveness of Mobile Health Coaching to Improve Periconceptional Nutrition and Lifestyle in Women: Survey in a Large Urban Municipality in the Netherlands. *JMIR mHealth and uHealth* 2019: **7**; e11664.

Gorczyca AM, Steger FL, Ptomey LT, Montgomery RN, Mickelsen R, Smith P, Donnelly JE, and Marsh CA. The impact of a group based, remotely delivered weight loss intervention in women with polycystic ovary syndrome on ovulation, quality of life and body composition. *Frontiers in reproductive health* 2022: **4**; 940945.

Gordon A, Muirhead R, Lal R, Kizirian N, Black K, Prys-Davies A, Baur L, Hyett J, Nassar N, Sainsbury A*, et al.* Pre-babe: Impact of pre-conception weight loss for women above a healthy weight. *Journal of paediatrics and child health* 2020: **56**; 17.

Haakstad LAH, Kissel I, and Bo K. Long-term effects of participation in a prenatal exercise intervention on body weight, body mass index, and physical activity level: a 6-year follow-up study of a randomized controlled trial. *The journal of maternal-fetal & neonatal medicine : the official journal of the European Association of Perinatal Medicine, the Federation of Asia and Oceania Perinatal Societies, the International Society of Perinatal Obstetricians* 2021: **34**; 1347-1355.

Halpern G, Arantes Sardinha FA, Setti A, Iaconelli Jr A, and Borges Jr E. Nutritional counseling impact on assisted reproduction treatment outcomes. *Jornal Brasileiro de Reproducao Assistida* 2013: **17**; 16-22.

Halpern G, Setti AS, de Almeida Ferreira Braga DP, Iaconelli A, and Borges E. Beetroot, watermelon and ginger juice supplementation may increase the clinical outcomes of intracytoplasmic sperm injection cycles. *Fertility and sterility* 2019: **112**; e3.

Hambidge KM, Krebs NF, Westcott JE, Garces A, Goudar SS, Kodkany BS, Pasha O, Tshefu A, Bose CL, Figueroa L*, et al.* Preconception maternal nutrition: A multi-site randomized controlled trial. *BMC pregnancy and childbirth* 2014: **14**; 111.

Hambidge KM, Krebs NF, Westcott JE, Garces A, Goudar SS, Kodkany BS, Pasha O, Tshefu A, Bose CL, Figueroa L*, et al.* Preconception maternal nutrition: A multi-site randomized controlled trial. *World review of nutrition and dietetics* 2016: **114**; 136-137.

Hammiche F, Laven JSE, van Mil N, de Cock M, de Vries JH, Lindemans J, Steegers EAP, and Steegers-Theunissen RPM. Tailored preconceptional dietary and lifestyle counselling in a tertiary outpatient clinic in The Netherlands. *Human reproduction (Oxford, England)* 2011: **26**; 2432-2441.

Hanafiah AN, Aagaard-Hansen J, Ch Cheah J, Norris SA, Karim ZB, Skau JK, Ali ZM, Biesma R, Matzen P, Sulaiman LH*, et al.* Effectiveness of a complex, pre-conception intervention to reduce the risk of diabetes by reducing adiposity in young adults in Malaysia: The Jom Mama project - A randomised controlled trial. *Journal of global health* 2022: **12**; 04053.

Hardy I, Lloyd A, Morisset A-S, Camirand Lemyre F, Baillargeon J-P, and Fraser WD. Healthy for My Baby Research Protocol- a Randomized Controlled Trial Assessing a Preconception Intervention to Improve the Lifestyle of Overweight Women and Their Partners. *Frontiers in public health* 2021: **9**; 670304.

Haresnape-Tyson C, Johnston A, Tucker AT, and Rivas C. The use of qualitative research, as part of a mixed methods design, to investigate the design and delivery of a placebo controlled, double blind, randomised clinical trial of a complex homeopathic intervention for women undergoing infertility treatment at Jersey General Hospital. *European Journal of Integrative Medicine* 2016: **8**; 588.

Harrison CL, Brammall BR, Garad R, and Teede H. OptimalMe Intervention for Healthy Preconception, Pregnancy, and Postpartum Lifestyles: Protocol for a Randomized Controlled Implementation Effectiveness Feasibility Trial. *JMIR research protocols* 2022: **11**; e33625.

Heckert J, Olney DK, and Ruel MT. Is women's empowerment a pathway to improving child nutrition outcomes in a nutrition-sensitive agriculture program?: Evidence from a randomized controlled trial in Burkina Faso. *Social Science and Medicine* 2019: **233**; 93-102.

Heerman WJ, Samuels LR, Barr L, Burgess LE, Hartmann KE, and Barkin SL. The Effect of a General Healthy Lifestyle Intervention Delivered Around Pregnancy on Gestational Weight Gain and Infant Growth. *Maternal and child health journal* 2020: **24**; 1404-1411.

Hillemeier MM, Downs DS, Feinberg ME, Weisman CS, Chuang CH, Parrott R, Velott D, Francis LA, Baker SA, Dyer A-M*, et al.* Improving Women's Preconceptional Health. *Women's Health Issues* 2008: **18**; S87-S96.

Hmedeh C, El Iskandarni S, and Tawfik I. The effect of 6-month nutritional intervention on the anthropometric, biochemical, and reproductive profile of Lebanese women with Polycystic ovarian syndrome. *Human Reproduction* 2021: **36**.

Hmedeh C, Ghazeeri G, Tinworth L, and Tewfik I. The effect of 6 months weight -loss/maintenance on anthropometric, biochemical and psychological profile in Lebanese PCOS women: A prospective randomised control study. *Human Reproduction* 2017: **32**.

Hojeij B, Schoenmakers S, Willemsen S, van Rossem L, Dinnyes A, Rousian M, and Steegers-Theunissen RP. The Effect of an eHealth Coaching Program (Smarter Pregnancy) on Attitudes and Practices Toward Periconception Lifestyle Behaviors in Women Attempting Pregnancy: Prospective Study. *Journal of medical Internet research* 2023: **25**; e39321.

Hojeij B, Schoenmakers S, Willemsen S, van Rossem L, Dinnyes A, Rousian M, Steegers-Theunissen RPM, AjzenI AAADAACABBBABMRBBATKBBHSTTABDA, et al., and et al. The effect of an ehealth coaching program (smarter pregnancy) on attitudes and practices toward periconception lifestyle behaviors in women attempting pregnancy: Prospective study. *Journal of medical Internet research* 2023: **25**.

Hollmann M, Runnebaum B, and Gerhard I. Effects of weight loss on the hormonal profile in obese, infertile women. *Human Reproduction* 1996: **11**; 1884-1891.

Huvinen E, Engberg E, Meinil, auml, Tammelin T, Kulmala J, Heinonen K, Bergman P, Stach-Lempinen B, and Koivusalo S. Lifestyle and glycemic health 5 years postpartum in obese and non-obese high diabetes risk women. *Acta diabetologica* 2020: **57**; 1453.
IRCT2016022826811N1. Comparison of effectiveness positive Iranian – Islamic therapy with Acceptance and Commitment Therapy on Depression, Anxiety, Stress and Psychological Well-being. *Comparison of effectiveness positive Iranian – Islamic therapy with Acceptance and Commitment Therapy on Depression, Anxiety, Stress and Psychological Well-being of Infertile Women in Isfahan City* 2016.
IRCT2016030226879N1. Effect of Preconception Counseling on Health Promoting Behaviors. *Effect of preconception counseling on health promoting behaviors of reproductive age women* 2016.
IRCT2015122325668N1. Effect of theory-based training on weight and knowledge of women about overweight before pregnancy. *The effect of education based on extended parallel process model on weight and knowledge of women about overweight before pregnancy* 2016.
IRCT2015011920719N1. The effect of counseling on feeding behavior change among overweight and obese women in pre-pregnancy period. *The effect of pre-pregnancy counseling on the feeding behavior of overweight and obese women admitted in health service clinics of Khansar?1395* 2017.
IRCT2016020223072N1. the Effect of Self Determination Theory (SDT) based physical activity motivational intervention on happiness and quality of life: A randomized controlled trial. 2017.
IRCT2016081629388N1. Impact of Diet Program based on Traditional Medicine on Ovulation and Fertility Rate in Woman with Induction of Ovulation. *Evaluation of Impact of Diet Program based on Traditional Medicine on Ovulation and Fertility Rate in Woman with Induction of Ovulation* 2017.
IRCT20200531047615N1. Effectiveness of positive intervention and Integrative-behavioral couple therapy on infertile couples. *Comparing the effectiveness of (PERMA) model and Integrative-behavioral couple therapy (IBCT) on fertility problems of couples under infertility treatment* 2020.
IRCT20210824052278N1. The effect of education based on health belief model on knowledge ,belief and performance of weight management of women with high body mass index in the before pregnancy period. *Evaluation of the effect of education intervention based on health belief model on knowledge ,belief and performance of weight management of women with high body mass index in the before pregnancy period in patients referred to comprehensive health center* 2021.
IRCT20220629055321N1. The effect of educational intervention on performing health behaviors before pregnancy of single-child couples: a clinical trial. *The effect of an educational intervention based on the integration of the theory of planned behavior and the theory of social cognition on performing health behaviors before pregnancy of single-child couples: a clinical trial* 2022.
IRCT20190529043754N1. The effect of health promoting lifestyle educational program on infertile couple who candidate for Assisted reproductive technology. *Determining the effectiveness of a health-promoting lifestyle training program in infertile couples under Assisted Reproductive Technologies (ART)* 2022.
IRCT20230307057652N1. Effect of self-management program on Quality of life and Self efficacy in women with Polycystic ovary syndrome. *Effectiveness of Self-management program on Quality of life and Self efficacy in women with Polycystic ovary syndrome* 2023.
IRCT20231004059612N1. The effect of sleep hygiene education and diet based on Persian traditional medicine texts on the results of in vitro fertilization (IVF/ICSI) of infertile women. 2023.
IRCT20220625055268N1. The efficacy of mindfulness-based stress reduction on psychological symptoms and quality of life in students with premenstrual syndrome in 1401. 2023.
IRCT20240207060925N1. The effect of lifestyle on Polycystic Ovary Syndrome. *The effect of lifestyle modification program on sexual function of infertile women with Polycystic Ovary Syndrome (PCOS)* 2024.
IRCT20240610062075N1. The effect of modification of lifestyle and temperament on the results of in vitro fertilization. *Investigating the effect of modification of lifestyle and temperament based*

on Iranian traditional medicine compared to routine treatment on the results of in vitro fertilization in overweight and obese infertile women 2024.
IRCT20240623062228N1. Investigating the effectiveness of mindfulness based cognitive therapy (MBCT) on stress and life expectancy in infertile women undergoing acupuncture treatment. 2024.
IRCT20210207050279N1. Modification of Complications Caused by Polycystic Ovary Syndrome by Nano Curcumin Drug and HIIT Exercise in Women Undergoing ICSI Treatment. *Effects of High Intensity Interval Training(HIIT) with Nano Curcumin Supplementation on MDA,SOD,GPX,TAC, LH,FSH,TT and Oocytes Maturation , Embryos Quality in Patients with PCOs Undergoing ICSI treatment. a Randomized Placebo-Controlled Clinical Trial* 2024.
IRCT2017013032245N2. The impact of diet based on Iranian Traditional Medicine on IVF/ICSI outcomes of infertile women. *Clinical trial comparing the effects of diet based on traditional medicine and usual diet on the results of in vitro fertilization for infertile women* 2017.
IRCT20230206057338N2. Investigating the effect of visceral manual therapy in improving uterine blood supply and pregnancy outcomes in infertile women with the problem of increased resistance of uterine vessels. *Investigating the effects of adding visceral manual therapy to routine pelvic physiotherapy compared to pharmacotherapy in improving uterine blood supply, and pregnancy outcomes in women with recurrent failure of implantation (RIF) with increased uterine* 2024.
IRCT2017052333834N4. The Effect of Motivational Interviewing on Dietary intake and Physical Activity Change of Overweight and Obese Preconceptional Women: A Randomized Controlled Clinical Trial. *https://trialsearch.who.int/Trial2.aspx?TrialID=IRCT2017052333834N4* 2017.
IRCT20160619028528N4. Effect of motivational interview on self-care and quality of life among infertile women with polycystic ovary syndrome. *Effect of Motivational Interview on Self-care(health promotional behaviors) and Quality of Life among infertile women with Polycystic Ovary Syndrome* 2020.
IRCT20220704055367N4. Designing an educational package for increasing childbearing. *The effect of the lifestyle modification educational package on the attitude to childbearing* 2022.
IRCT20150119020719N6. The effect of counseling on stress and female behaviors of infertile women. *Effect of group counseling on stress and gender role attitude of infertile women* 2018.
IRCT20160619028528N7. The effect of online brief mindfulness-based counseling on infertility related distress in infertile women under treatment. *The effect of online brief mindfulness-based counseling on infertility related distress in infertile women under treatment: a randomized controlled clinical trial* 2023.
IRCT20180218038783N8. Effectiveness of telephone counseling based on mindfulness on sexual anxiety of infertile women. *Effectiveness of telephone counseling based on mindfulness on sexual anxiety of infertile women of Babol city* 2024.
IRCT20160608028352N13. The effects of motivational interviewing focused on some principles of Hefz – Alsehe in Persian medicine on stress and quality of life of infertile women. 2023.
IRCT2015082013405N14. The effect mindfulness-based counseling on anxiety in infertile women undergoing IVF. *The effect of mindfulness-based counseling by the virtual method on anxiety in infertile women undergoing In Vitro Fertilization(IVF)* 2023.
IRCT20120215009014N477. Effect of lifestyle counseling versus control group on premature ovarian insufficiency in infertile women. *Effect of lifestyle counseling versus control group on premature ovarian insufficiency in infertile women: a randomized clinical trial* 2023.
IRCT20120215009014N509. Effect of counseling versus control group on fertility knowledge and childbearing attitude among infertile couples. *Effect of counseling versus control group on fertility knowledge and childbearing attitude among infertile couples: a randomized clinical trial* 2024.

ISRCTN01915371. Weight loss in obese women with Polycystic Ovary Syndrome (PCOS). *Weight loss in obese women with Polycystic Ovary Syndrome (PCOS): a randomised controlled trial* 2011.

ISRCTN11081163. A Swedish population-based reproductive life plan intervention. *Reproductive life plan intervention - a Swedish randomized controlled trial* 2016.

ISRCTN13308752. Preconception-early childhood telephone-based intervention to optimize growth and development among children in Canada: A Healthy Life Trajectory Initiative (HeLTI-Canada). *A preconception-early childhood telephone-based intervention with tailored e-health resources for women and their partners to optimise growth and development among children in Canada: A Healthy Life Trajectory Initiative (HeLTI-Canada)* 2019.

ISRCTN44294662. Effectiveness of a diet information website targeting young adults before parenthood. *Effectiveness of a web-based dietary intervention program targeting young adults prior to parenthood: a randomized controlled trial (PREPARED)* 2021.

ISRCTN97130017. Preconception care and gestational diabetes prevention in Bangladesh. *Preconception care and its effect on the prevention of gestational diabetes in Bangladesh* 2023.

Jackson B, Kishan R, Mullins C, Mathew M, Kim S, Huang JC, and Phy JL. NUTRITIONAL EDUCATION (FACE-TO-FACE AND VIDEO INSTRUCTION) FOR POLYCYSTIC OVARY SYNDROME RESULTS IN GREATER REDUCTION IN BMI AND HEMOGLOBIN A1C THAN CALORIC RESTRICTION, EXERCISE AND METFORMIN. *Fertility and sterility* 2022: **118**; e95.

Jamebozorg N, Ghafari F, Alijaniha F, Karimi Y, Beigi RM, Haghani H, Naseri M, and Samani LN. The Effect of Metabolic Persian Diet on Ovulation Induction in Infertile Women. *Evidence-based complementary and alternative medicine* 2023: **2023**.

Jamebozorg N, Samani LN, Naseri M, Beigi RM, and Haghani H. The effect of iranian traditional medicine based dietary intervention on incidence of pregnancy in women undergoing ovulation stimulation. *Journal of Reproduction and Infertility* 2018: **19**; 160-161.

James PT, Jawla O, Mohammed NI, Ceesay K, Akemokwe FM, Sonko B, Sise EA, Prentice AM, and Silver MJ. A novel nutritional supplement to reduce plasma homocysteine in nonpregnant women: A randomised controlled trial in The Gambia. *PLoS medicine* 2019: **16**; e1002870.

Jiskoot G, Timman R, Beerthuizen A, Dietz de Loos A, Busschbach J, and Laven J. Weight Reduction Through a Cognitive Behavioral Therapy Lifestyle Intervention in PCOS: The Primary Outcome of a Randomized Controlled Trial. *Obesity* 2020: **28**; 2134-2141.

JPRN-UMIN000027424. Impact of guidance and consultation management program on women undergoing non-ART infertility treatment. 2017.

Karsten MDA, van Oers AM, Groen H, Mutsaerts MAQ, van Poppel MNM, Geelen A, van de Beek C, Painter RC, Mol BWJ, Roseboom TJ*, et al.* Determinants of successful lifestyle change during a 6-month preconception lifestyle intervention in women with obesity and infertility. *European journal of nutrition* 2019: **58**; 2463-2475.

Kaya Y, Kizilkaya Beji N, Aydin Y, and Hassa H. The effect of health-promoting lifestyle education on the treatment of unexplained female infertility. *European journal of obstetrics, gynecology, and reproductive biology* 2016: **207**; 109-114.

KCT0008022. The effect of preconception care program for women with inflammatory bowel disease: a mixed method study. 2022.

KCT0008931. Effects of web-based premature birth prevention self-management education program. *Effects of web-based premature birth prevention self-management education program in women of childbearing age* 2023.

KCT0008950. Effect of the Korea Integrated Support Program for Infertile Women (KISPIW): a Pilot and Feasibility Randomized Controlled Trial. 2023.

Kermack A, Lowen P, Wellstead S, Fisk H, Montag M, Cheong Y, Osmond C, Houghton F, Calder P, and MacKlon N. The effect of a 6-week dietary supplement of omega- 3 fatty acids and vitamin D on in vitro human embryo development: The 'PREPARE*' double-blind randomised controlled trial. *BJOG: An International Journal of Obstetrics and Gynaecology* 2020: **127**; e57-e58.

Kermack AJ, Calder PC, Houghton FD, Godfrey KM, and Macklon NS. A randomised controlled trial of a preconceptional dietary intervention in women undergoing IVF treatment (PREPARE trial). *BMC women's health* 2014: **14**; 130.

Kermack AJ, Lowen P, Wellstead SJ, Fisk HL, Montag M, Cheong Y, Osmond C, Houghton FD, Calder PC, and Macklon NS. Effect of a 6-week "Mediterranean" dietary intervention on in vitro human embryo development: the Preconception Dietary Supplements in Assisted Reproduction double-blinded randomized controlled trial. *Fertility and sterility* 2020: **113**; 260-269.

Kermack AJ, Lowen PK, Wellstead SJ, Fisk HL, Montag M, Houghton FD, Calder PC, and Macklon NS. PREPARE trial: A randomised double blinded controlled trial of a preconception Omega 3 and vitamin D rich dietary supplement in couples undergoing assisted reproduction treatment. *Human Reproduction* 2017: **32**.

Kermack AJ, Wellstead SJ, Fisk HL, Cheong Y, Houghton FD, Macklon NS, and Calder PC. The Fatty Acid Composition of Human Follicular Fluid Is Altered by a 6-Week Dietary Intervention That Includes Marine Omega-3 Fatty Acids. *Lipids* 2021: **56**; 201-209.

Keytash A. Impact of preconception care on overweight/obese women: Long-term effects of a behavioural change intervention. *Journal of paediatrics and child health* 2015: **51**; 111.

Kiel IA, Lionett S, Parr EB, Jones H, Roset MAH, Salvesen O, Hawley JA, Vanky E, and Moholdt T. High-Intensity Interval Training in Polycystic Ovary Syndrome: A Two-Center, Three-Armed Randomized Controlled Trial. *Medicine and science in sports and exercise* 2022: **54**; 717-727.

Kiel IA, Lionett S, Parr EB, Jones H, Roset MAH, Salvesen O, Vanky E, and Moholdt T. Improving reproductive function in women with polycystic ovary syndrome with high-intensity interval training (IMPROV-IT): study protocol for a two-centre, three-armed randomised controlled trial. *BMJ Open* 2020: **10**; e034733.

Kiel IA, Lundgren KM, Mørkved S, Kjøtrød SB, Salvesen Ø, Romundstad LB, and Moholdt T. Women undergoing assisted fertilisation and high-intensity interval training: a pilot randomised controlled trial. *BMJ Open Sport & Exercise Medicine* 2018: **4**; e000387.

Killeen SL, Byrne DF, Geraghty AA, Yelverton CA, van Sinderen D, Cotter PD, Murphy EF, O'Reilly SL, and McAuliffe FM. Recruiting and Engaging Women of Reproductive Age with Obesity: Insights from A Mixed-Methods Study within A Trial. *International journal of environmental research and public health* 2022: **19**.

Kirca N and Pasinlioglu T. The effect of yoga on stress level in infertile women. *Perspectives in psychiatric care* 2019: **55**; 319-327.

Kiyak S and Kocoglu-Tanyer D. Effectiveness of progressive muscle relaxation and laughter therapy on mental health and treatment outcomes in women undergoing in vitro fertilization: A randomized controlled trial. *Research in nursing & health* 2021: **44**; 945-956.

Koduri P, Parimala C, Kunjummen AT, Yadav BK, Kapoor N, Sandhiya P, Vimala, and Kamath MS. Individualised Lifestyle Intervention in Polycystic Ovarian Syndrome Women Who Desire Fertility: A Feasibility Study. *J Hum Reprod Sci* 2024: **17**; 207-215.

Krebs NF, Figueroa L, Ali SA, Metgud D, Lokangaka AL, Westcott J, Das A, Chowdhury D, and Hambidge KM. Neurodevelopment scores are associated with multiple components of nurturing care in the 24 month old offspring of participants in the multi-country "Women First" preconception maternal nutrition trial. *Annals of Nutrition and Metabolism* 2023: **79**; 327

EP-328.

Krebs NF, Hambidge KM, Westcott JL, Garces AL, Figueroa L, Tsefu AK, Lokangaka AL, Goudar SS, Dhaded SM, Saleem S*, et al.* Growth from Birth Through Six Months for Infants of Mothers in the "Women First" Preconception Maternal Nutrition Trial. *The Journal of pediatrics* 2021: **229**; 199-206.e194.

Kumaran K, Birken C, Baillargeon J-P, Dennis C-L, Fraser WD, Huang H, Fan J, Lye S, Matthews SG, and Norris SA. An intergenerational life-course approach to address early childhood obesity and adiposity: the Healthy Life Trajectories Initiative (HeLTI). *The Lancet. Global health* 2023: **11 Suppl 1**; S15.

Kumaran K, Krishnaveni GV, Suryanarayana KG, Prasad MP, Belavendra A, Atkinson S, Balasubramaniam R, Bandsma RHJ, Bhutta ZA, Chandak GR*, et al.* Protocol for a cluster randomised trial evaluating a multifaceted intervention starting preconceptionally-Early Interventions to Support Trajectories for Healthy Life in India (EINSTEIN): a Healthy Life Trajectories Initiative (HeLTI) Study. *BMJ Open* 2021: **11**; e045862.

Lawande A, Gravio CD, Potdar RD, Sahariah SA, Gandhi M, Chopra H, Sane H, Kehoe SH, Marley-Zagar E, and Fall CHD. Effect of a micronutrient-rich snack taken pre-conceptionally and throughout pregnancy on ultrasound measures of fetal growth: The Mumbai Maternal Nutrition Project (MMNP). *Maternal and Child Nutrition* 2018: **14**.

LeBlanc E, Boisvert C, Catlin C, Lee M, Smith N, Vesco K, Savage J, Mitchell D, Gruss I, and Stevens V. The prepare pre-pregnancy weight loss intervention: Efficacy on diet and exercise and acceptability. *Obesity* 2021: **29**; 176.

Leblanc ES, Smith NX, Vesco KK, Paul IM, and Stevens VJ. Weight loss prior to pregnancy and subsequent gestational weight gain: Prepare, a randomized clinical trial. *American Journal of Obstetrics and Gynecology* 2021: **224**; 99.e91-99.e14.

Legro RS, Dodson WC, Kunselman AR, Kris-Etherton PM, Allison KC, Sarwer DB, Dokras A, and Coutifaris C. Effects of preconception intervention on the PCOS phenotype, ovulation, and live birth rates: A multicenter, multi-phase RCT. *Fertility and sterility* 2014: **102**; e2.

Li Y. Preconception lifestyle intervention reduces gestational diabetes recurrence: a randomized controlled trial. *:-* 2023: **26**; 365.

Liu Q and Yan C. The application of information-motivation-behavior skill model in patients with moderate to severe ovarian hyperstimulation syndrome. *Signa Vitae* 2022: **18**; 110-115.

Liu R, Li M, Wang P, Yu M, Wang Z, and Zhang G-Z. Preventive online and offline health management intervention in polycystic ovary syndrome. *World journal of clinical cases* 2022: **10**; 3060-3068.

Lumley J and Donohue L. Aiming to increase birth weight: a randomised trial of pre-pregnancy information, advice and counselling in inner-urban Melbourne. *BMC Public Health* 2006: **6**.

Ly M, Price S, and Ekinci EI. Impact of substantial weight loss on thyroid function in obese women planning pregnancy. *Clinical endocrinology* 2017: **86**; 52-53.

Maas VYF, Koster MPH, Ista E, Vanden Auweele KLH, de Bie RWA, de Smit DJ, Visser BC, van Vliet-Lachotzki EH, Franx A, and Poels M. Study design of a stepped wedge cluster randomized controlled trial to evaluate the effect of a locally tailored approach for preconception care - the APROPOS-II study. *BMC public health* 2020: **20**; 235.

Maas VYF, Poels M, Ista E, Menge LF, Vanden Auweele KLHE, de Bie RWA, de Smit DJ, van Vliet-Lachotzki EH, Franx A, and Koster MPH. The effect of a locally tailored intervention on the uptake of preconception care in the Netherlands: a stepped-wedge cluster randomized trial (APROPOS-II study). *BMC public health* 2022: **22**; 1997.

Maillet D, Alj A, Belan M, Jean-Denis F, Rouissi M, and Baillargeon JP. Evaluating the Impact of a 6-month Lifestyle Intervention Program on Quality of Life and Motivation in Women With Obesity and Infertility. *Canadian journal of diabetes* 2021: **45**; S38.

Malekpour P, hasanzadeh R, Javedani Masroor M, Chaman R, and Motaghi Z. Effectiveness of a mixed lifestyle program in couples undergoing assisted reproductive technology: a study protocol. *Reproductive health* 2023: **20**; 112.

Malhotra N, Arora T, Suri V, Jena SK, Verma A, Gowri M, Kapoor N, Chalga MS, Kulkarni B, and Kamath MS. Individualized lifestyle intervention in PCOS women (IPOS): a study protocol for a multicentric randomized controlled trial for evaluating the effectiveness of an individualized lifestyle intervention in PCOS women who wish to conceive. *Trials* 2023: **24**; 457.

Mani H, Chudasama Y, Hadjiconstantinou M, Bodicoat DH, Edwardson C, Levy MJ, Gray LJ, Barnett J, Daly H, Howlett TA*, et al.* Structured education programme for women with polycystic ovary syndrome: a randomised controlled trial. *Endocrine connections* 2018: **7**; 26-35.

Manteghi G, Shahraki Z, Moghadam MN, and Ghanbarpour MH. Pregnancy outcome in PCOS patients: the effects of letrozol combined with exercise. *Pregnancy outcome in PCOS patients: the effects of letrozol combined with exercise* 2021: **14**; 128.

Matsuzaki T, Douchi T, Oki T, Ishihara O, Okagaki R, Kajihara T, Tamura M, Kotsuji F, Tajima K, Kawano M*, et al.* Weight reduction by using a formula diet recovers menstruation in obese patients with an ovulatory disorder. *Reproductive medicine and biology* 2017.

Maunder A, Arentz S, Armour M, Costello MF, and Ee C. Effectiveness of naturopathy for pregnancy in women with diminished ovarian reserve: feasibility randomized controlled trial. *Reproductive biomedicine online* 2024: **48**; 103844.

McBreairty L, Zello G, Rooke J, Serrao S, Pierson R, Chizen D, and Chilibeck P. A pulse-based diet and exercise training in women with polycystic ovarian syndrome: effects on body composition, blood lipids and reproductive measures. *FASEB Journal* 2014: **28**.

McBreairty L, Zello G, Rooke J, Serrao S, Pierson R, Chizen D, and Chilibeck P. Long-term effect of a pulse-based diet and exercise training intervention on body composition and dietary intake in women with polycystic ovarian syndrome. *FASEB Journal* 2015: **29**.

McBreairty LE, Chilibeck PD, Chizen DR, Pierson RA, Tumback L, Sherar LB, and Zello GA. The role of a pulse-based diet on infertility measures and metabolic syndrome risk: protocol of a randomized clinical trial in women with polycystic ovary syndrome. *BMC nutrition* 2017: **3**; 23.

McBreairty LE, Kazemi M, Gordon JJ, Pierson RA, Chizen DR, Chilibeck PD, and Zello GA. A randomized clinical trial in women with polycystic ovary syndrome: Effects of a pulse-based diet and exercise intervention on blood lipids, body composition and reproductive measures. *FASEB Journal* 2017: **31**.

Mead LC, Mitchell M, Deussen AR, and Dodd JM. Motivational interviewing in the preconception period for women with overweight or obesity. *Obesity research & clinical practice* 2024: **18**; S46.

Meenakshi M, Anitha A, Ramana K, and Kamalakannan M. Efficacy of Swiss Ball Exercise and Resistance Training in Polycystic Ovarian Syndrome. *Indian Journal of Physiotherapy & Occupational Therapy* 2024: **18**; 302-309.

Meneghini C, Bianco C, Galanti F, Tamburelli V, Dal Lago A, Licata E, Gallo M, Fabiani C, Corno R, Miriello D*, et al.* The Impact of Nutritional Therapy in the Management of Overweight/Obese PCOS Patient Candidates for IVF. *Nutrients* 2023: **15**.

Mogensen CS, Zingenberg H, Svare J, Astrup A, Magkos F, and Geiker NRW. Gestational weight gain in women with pre-pregnancy overweight or obesity and anthropometry of infants at birth. *Frontiers in pediatrics* 2023: **11**; 1142920.

Mohseni M, Eghbali M, Bahrami H, Dastaran F, and Amini L. Yoga Effects on Anthropometric Indices and Polycystic Ovary Syndrome Symptoms in Women Undergoing Infertility Treatment: A Randomized Controlled Clinical Trial. *Evidence-based complementary and alternative medicine : eCAM* 2021: **2021**; 5564824.

Mol BWJ, Van Oers AM, Mutsaerts MAQ, Land JA, Groen H, and Hoek A. Effects of preconceptional weight loss on maternal and fetal outcome in obese subfertile women. *Journal of paediatrics and child health* 2015: **51**; 36.

Montanaro C, Robson L, Binnington L, Winters N, and Brown HK. Validating PreCHAT: A Digital Preconception Health Risk Assessment Tool to Improve Reproductive, Maternal and Child Health. *Canadian Journal of Nursing Research* 2023: **55**; 206-215.

Moore AK, Rasmussen R, Sandberg J, Holt-Lunstad J, Carrell DT, Straseski J, Peterson CM, and Johnstone EB. Intensive lifestyle intervention including emotionally-focused couples therapy leads to more pregnancies and weight loss in obese infertile couples. *Fertility and sterility* 2016: **106**; e101.

Moran L, Tsagareli V, Norman R, and Noakes M. Diet and IVF pilot study: Short-term weight loss improves pregnancy rates in overweight/obese women undertaking IVF. *Australian and New Zealand Journal of Obstetrics and Gynaecology* 2011: **51**; 455-459.

Mostajabi SZ, Shoorab NJ, Kordi M, and Esmaily H. The Effect of Training Based on Extended Parallel Process Model on Weight of Women with High Body Mass Index: A Cluster Randomized Trial. *Journal of midwifery & reproductive health* 2022: **10**; 3462-3471.

Muirhead R, Kizirian N, Lal R, Black K, Prys-Davies A, Nassar N, Baur L, Sainsbury A, Sweeting A, Markovic T*, et al.* A Pilot Randomized Controlled Trial of a Partial Meal Replacement

Preconception Weight Loss Program for Women with Overweight and Obesity. *Nutrients* 2021: **13**.
Mutsaerts MAQ, Van Oers AM, Groen H, Burggraaff JM, Kuchenbecker WKH, Perquin DAM, Koks CAM, Van Golde R, Kaaijk EM, Schierbeek JM*, et al.* Randomized Trial of a Lifestyle Program in Obese Infertile Women. *Obstetrical & gynecological survey* 2016: **71**; 533.
Mutsaerts MAQ, Van Oers AM, Groen H, Burggraaff JM, Kuchenbecker WKH, Perquin DAM, Koks CAM, Van Golde R, Kaaijk EM, Schierbeek JM*, et al.* Randomized Trial of a Lifestyle Program in Obese Infertile Women. *New England Journal of Medicine* 2016: **374**; 1942-1953.
Nagelberg J, Burks H, Mucowski S, and Shoupe D. The effect of home exercise on ovulation induction using clomiphene citrate in overweight underserved women with polycystic ovarian syndrome. *Contraception and reproductive medicine* 2016: **1**; 14.
Nasrekani ZA and Fathi M. Efficacy of 12 weeks aerobic training on body composition, aerobic power and some women-hormones in polycystic ovary syndrome infertile women. *Iranian Journal of Obstetrics, Gynecology and Infertility* 2016: **19**; 1-10.
Nayar P, Nayar KD, Ahuja R, Singh M, Kant G, Sharma N, and Nayar K. Can yoga affect IVF outcomes? *Fertility and sterility* 2017: **108**; e300.
Nayar P, Nayar KD, Singh M, Aggarwal N, Gupta M, Gupta RA, Kant G, Gahlot R, and Nayar K. Yoga as an adjuvant to enhance the outcome of IVF treatment. *Human Reproduction* 2018: **33**.
Nayar P, Obst D, Sethi A, Sanan S, Nayar S, Nayar KD, Kant G, and Kashyap P. IMPACT OF PSYCHOSOCIAL SUPPORT WITH MEDITATION ON IVF/ICSI CLINICAL OUTCOMES: A PROSPECTIVE COHORT STUDY. *Fertility and sterility* 2023: **120**; e60.
NCT00679679. Metformin and Lifestyle Intervention in Women With Polycystic Ovary Syndrome. *Clinical Metabolic and Endocrine Parameters in Response to Metformin and Lifestyle Intervention in Women With Polycystic Ovary Syndrome: A Phase 4 Randomized, Double-Blind and Placebo Control Trial* 2008.
NCT01028989. A Reduced Carbohydrate Diet Intervention for Polycystic Ovary Syndrome (PCOS). *A Reduced Carbohydrate Diet Intervention for PCOS* 2009.
NCT01483612. Evaluation of Clinical Outcomes and Costs of a Lifestyle Intervention in Obese Infertile Women. *"Evaluation of Clinical Outcomes and Costs of a Transferable Interdisciplinary Lifestyle Intervention Pre- and Per-pregnancy in Obese Infertile Women"* 2011.
NCT01892111. Physical Activity and ARTs. *https://clinicaltrials.gov/study/NCT01892111* 2013.
NCT01894074. The Effects of an Intensive Lifestyle Intervention on Reproductive Outcomes. *The Effects of an Intensive Lifestyle Intervention on Reproductive Outcomes in Obese, Subfertile Women* 2013.
NCT01933633. Improved Fertility After Exercise in Overweight/Obese Women. *Study of Pregnancy Rate After Exercise Training Prior to Assisted Fertilisation in Overweight/Obese Women* 2013.
NCT01952795. Effect of Hypocaloric Diet and Exercise in Obese Women Who Are Subjected to IVF Cycle. *Effect of Hypocaloric Diet and Exercise in Obese Women Who Are Subjected to IVF Cycle* 2013.
NCT02049554. Preconception Women's Health in Pediatric Practice Intervention. 2014.
NCT02617693. Development of Pre-pregnancy Intervention to Reduce the Risk of Diabetes and Prediabetes. *Jom Mama Project - Pre-pregnancy Intervention to Reduce the Risk of Diabetes and Prediabetes* 2015.
NCT02630485. Graceful Lifestyle Changes Study for PCOS and Infertility. *Graceful Lifestyle Changes Intervention Study for Women With PCOS and Infertility* 2015.
NCT02648555. A Lifestyle Intervention to Improve in Vitro Fertilization Results. *A Lifestyle Intervention to Improve in Vitro Fertilization Results* 2016.
NCT02763150. Pre-pregnancy Lifestyle Intervention to Prevent the Recurrence of Gestational Diabetes in Overweight and Obese Women. *Gestational Diabetes Prevention Program* 2016.

NCT02945488. Exercise and Nutrition in IBD & Preconception. *Exercise and Nutrition as Treatment Alternatives in Women With Inflammatory Bowel Disease During Preconception: Saskatchewan Multidisciplinary Inflammatory Bowel Diseases Clinic (MDIBDC)* 2016.

NCT03012412. Mindfulness Based Program for Infertility. 2016.

NCT03023137. Walking and Dietary Modification for Recurrent Early Miscarriages. *Walking and Dietary Modification for Women With Consecutive Early Miscarriages: a Randomized Study* 2017.

NCT03085212. Strategies for Pregnancy Achievement. *Strategies for Pregnancy Achievement (SPA): a Pilot Study* 2017.

NCT03146156. Lifestyle Intervention in Preparation for Pregnancy (LIPP). *Lifestyle Intervention in Preparation for Pregnancy (LIPP)* 2017.

NCT03343405. Online Mind/Body Program for Fertility. *Fertility & Well-Being: mind/Body Protocol* 2017.

NCT03395067. Multidisciplinary Treatment of Obesity Prior to in Vitro Fertilization: impact on Global Reproductive Outcomes (PRO-FIV Study). *Multidisciplinary Treatment of Obesity Prior to In Vitro Fertilization: Impact on Global Reproductive Outcomes* 2017.

NCT03703115. The Effect of Fasting on ICSI Outcomes in Patients With Polycystic Ovary Syndrome. *The Effect of Fasting on ICSI Outcomes in Patients With Polycystic Ovary Syndrome* 2018.

NCT03790449. Evaluation of a Mobile Preconception Lifestyle Programme in Couples Undergoing In Vitro Fertilisation. *Evaluation of a Mobile Preconception Lifestyle Programme in Couples Undergoing in Vitro Fertilisation: a Multicentre Randomized Controlled Trial (PreLiFe-RCT)* 2018.

NCT03898037. Effect of Lifestyle and/or Metformin Intervention on Pregnancy Outcome, A Pilot Randomized Controlled Trial. *Effect of Lifestyle and/or Metformin Intervention on Pregnancy Outcome in Overweight/Obese Non-pcos Infertility Women With Insulin Resistance , A Pilot Randomized Controlled Trial* 2019.

NCT03908099. Fit-for-Fertility Multicenter Randomized Controlled Trial. *Fit-for-Fertility Multicenter Randomized Controlled Trial: Improving Reproductive, Maternal and Neonatal Outcomes in Obese and Infertile* 2019.

NCT04242069. Healthy for my Baby- RCT of a Lifestyle Intervention for Overweight Women in Preconception. *Healthy for my Baby- A Randomized Controlled Trial Assessing a Preconception Clinically Integrated Technological Intervention to Improve the Lifestyle of Overweight Women and Their Partners* 2020.

NCT04273048. The MOM TO BE Study. *A Dietary and Exercise Intervention in Overweight and Obese Women Undergoing Fertility Treatment* 2019.

NCT04275869. Internet-based Intervention to Promote a Healthy Lifestyle on the Reproductive Parameters of Overweight and Obese Women. *Efficacy of an Internet-based Intervention to Promote a Healthy Lifestyle on the Reproductive Parameters of Overweight and Obese Women: a Randomised Controlled Trial* 2020.

NCT04335227. Yoga Therapy and Aerobic Exercise on Anti-Mullerian Hormone in Young Women With Polycystic Ovary Syndrome. *Yoga Therapy and Aerobic Exercise on Anti-Mullerian Hormone and Other Biochemical Markers in Young Women With Polycystic Ovary Syndrome* 2020.

NCT04416620. The Impact of a Pharmaceutical Care Model on Improving Polycystic Ovary Syndrome. *The Impact of a Pharmaceutical Care Model on Improving Polycystic Ovary Syndrome Associated Factors Amongst Females in Jordan and Syria* 2020.

NCT04419740. Impact of a Mindfulness Intervention on Infertile Women Undergoing Assisted Reproductive Technique Treatments. *What is the Impact of an E-tool Based Mindfulness Intervention on Psychological Outcomes Compared to no Intervention in Infertile Women Undergoing Assisted Reproductive Technique Treatments: a Randomized Controlled Study?* 2019.

NCT04585581. Preconception Lifestyle Interventions to Improve Future Metabolic Health (Before the Beginning). *Before the Beginning: Preconception Lifestyle Interventions to Improve Future Metabolic Health* 2020.

NCT04589793. COaching Lifestyle Intervention for FErtility. *COaching Lifestyle Intervention for FErtility* 2020.
NCT04942457. Efficacy of Fasting on Hormone Dosage in Fertility Treatment. *Effects of Fasting on Hormone Dosage in Fertility Treatment in Women With Ovulation Disorders* 2021.
NCT04942457. Feasibility and Safety of Fasting in Fertility Treatment. *Effects of Fasting in Fertility Treatment in Women* 2021.
NCT04976881. The PREPARED Trial. *Promoting Preconception Care and Diabetes Self-Management Among Reproductive-Aged Women With Diabetes: The PREPARED Trial* 2021.
NCT04998591. Effects of Fasting on Success Rates of Assisted Reproductive Techologies. *Effects of Fasting on Success Rates of In-vitro-fertilization (IVF) / Intracytoplasmatic Sperm Injection (ICSI) in Women With Fertility Disorders* 2021.
NCT05084274. Optimising Preconceptual Health in Subfertile PCOS Patients Using a Lifestyle Modification Program. *Optimising Preconceptual Health in Subfertile PCOS Patients. Implementation of a Lifestyle Modification Program Before Fertility Treatment, Impact on Health Related Quality of Life and Obstetric Outcome* 2021.
NCT05299450. Pre-conception Interventions to Reduce Blood Pressure Before Pregnancy. *Pre-conception Interventions to Reduce Blood Pressure Before Pregnancy* 2022.
NCT05578690. Healthy Lifestyle Before and During Pregnancy to Prevent Childhood Obesity - the PRE-STORK-trial. *Healthy Lifestyle Before and During Pregnancy to Prevent Childhood Obesity. A Randomized, Parallel Group, Tailored Multifactorial Lifestyle Intervention Trial Among Women With Overweight or Obesity Seeking Pregnancy - the PRE-STORK Trial* 2022.
NCT05621109. PRE-Pregnancy Weight Loss And the Reducing Effect on CHILDhood Overweight - Copenhagen. *PRE-Pregnancy Weight Loss And the Reducing Effect on CHILDhood Overweight - a Randomized Controlled Study in Copenhagen* 2022.
NCT05629858. Time Restricted Eating for the Treatment of PCOS. *Time Restricted Eating for the Treatment of Polycystic Ovarian Syndrome (PCOS)* 2022.
NCT05651568. Effect of Periodized Resistance Training and High Intensity Training on BMI and QOL in PCOS. *Comparison of Periodized Resistance Training and High Intensity Interval Training on Body Mass Index and Quality of in Polycystic Ovarian Syndrome* 2022.
NCT05674799. NDPP-NextGen: a Clinical Trial to Reduce Intergenerational Obesity and Diabetes Risks. 2022.
NCT05708937. THE EFFECT OF COGNITIVE AWARENESS SUPPORT ON THE PSYCHOSOCIAL STATUS OF WOMEN TREATED FOR INFERTILITY. *Assistant Proffessor Ph.D* 2023.
NCT05798494. PRE-Pregnancy Weight Loss And the Reducing Effect on CHILDhood Overweight - Aarhus. 2023.
NCT06044545. Effect Web Based Pregnancy Preparation Education According to Health Promotion Model in Preconceptional Period on Women. *The Effect of Web Based Pregnancy Preparation Education Structured According to the Health Promotion Model in the Preconceptional Period on Women's Knowledge Attitude and Health Behaviors* 2023.
NCT06049186. CRD vs. Met in Patients With Obese PCOS Infertility. *Comparison of Live Birth Rates Between Calorie-restricted Diets and Metformin Interventions Prior to Ovulation Induction Therapy in Patients With Overweight/Obese Polycystic Ovary Syndrome Combined With Infertility* 2023.
NCT06100523. PREVenting-ENvIronment-Reprotoxic Exposures Before In Vitro Fertilization (PREVENIR-FIV). *Multidisciplinary Platform Designed to Reduce and Prevent Environmental Reprotoxic Exposures in Subfertile Couples: assessment of the Contribution of Such Platforms on the Birth Rate After In Vitro Fertilization: randomized Open Prospective Comparative Mu* 2023.
NCT06175520. Introduction of Preconception Care Through Public Health System for Improving MNCH&FP. *Introduction of Preconception Care Through the Public Health System to Improve Maternal Health and Family Planning Service Utilizations Among Young Women in Rural Bangladesh: a Cluster Randomized Controlled Trial* 2023.

NCT06402825. Follow-up of Children Born From a Preconception Lifestyle Intervention in Women With Obesity and Infertility. *Follow-up of Children Born From a Randomized Controlled Trial Assessing a Preconception Lifestyle Intervention in Women With Obesity and Infertility* 2024.

NCT06591637. Fertility Supportive Behavior Education Based On Watson's Theory Of Human Care. *The Effects Of Fertility Supportive Behavior Education Based On Watson's Theory Of Human Care On Healthy Lifestyle Behaviors And İn Vitro Fertilization (IVF) Success İn Women With Primary Infertility* 2024.

Nery SF, Paiva SPC, Vieira ÉL, Barbosa AB, Sant'Anna EM, Casalechi M, Dela Cruz C, Teixeira AL, and Reis FM. Mindfulness-based program for stress reduction in infertile women: Randomized controlled trial. *Stress & health: journal of the international society for the investigation of stress* 2019: **35**; 49-58.

Ng KYB, Steegers-Theunissen R, Willemsen S, Wellstead S, Cheong Y, and Macklon N. Smartphone-based lifestyle coaching modifies behaviours in women with subfertility or recurrent miscarriage: a randomized controlled trial. *Reproductive BioMedicine Online* 2021: **43**; 111-119.

Ng KYB, Steegers-Theunissen R, Willemsen SP, Wellstead S, Cheong Y, and Macklon N. RCT of the impact of the online lifestyle coaching platform 'Smarter Pregnancy' on modifying peri-conceptional behaviours in women presenting with subfertility or recurrent miscarriages. *Human Reproduction* 2019: **34**.

Nga HT, Quyen PN, Chaffee BW, Diep Anh NT, Ngu T, and King JC. Effect of a nutrient-rich, food-based supplement given to rural Vietnamese mothers prior to and/or during pregnancy on birth outcomes: A randomized controlled trial. *PloS one* 2020: **15**; e0232197.

Niederberger C. Re: Moderate Aerobic Exercise Training for Improving Reproductive Function in Infertile Patients: A Randomized Controlled Trial. *The Journal of urology* 2017: **198**; 241-242.

Niederberger C. Re: High-Intensity Exercise Training for Improving Reproductive Function in Infertile Patients: A Randomized Controlled Trial. *The Journal of urology* 2018: **199**; 331.

NL-OMON21869. Optimizing periconceptional lifestyle of overweight women using a blended personalized care intervention combining eHealth and Face-to-face coUnSEling: The Randomized Controlled eFUSE Trial. *eFUSE: Optimizing periconceptional lifestyle of overweight women using a blended personalized care* 2021.

NL-OMON39189. Longterm lifestyle behaviour modification in overweighted women with polycystic ovary syndrome (PCOS). *Longterm lifestyle behaviour modification in overweighted women with polycystic ovary syndrome (PCOS). - PCOS and overweight* 2008.

Norris SA, Draper CE, Prioreschi A, Smuts CM, Ware LJ, Dennis C, Awadalla P, Bassani D, Bhutta Z, Briollais L*, et al.* Building knowledge, optimising physical and mental health and setting up healthier life trajectories in South African women (Bukhali): a preconception randomised control trial part of the Healthy Life Trajectories Initiative (HeLTI). *BMJ Open* 2022: **12**; e059914.

Nourizadeh R, Azami S, Farshbaf-Khalili A, and Mehrabi E. The Effect of Motivational Interviewing on Women with Overweight and Obesity Before Conception. *Journal of nutrition education and behavior* 2020: **52**; 859-866.

NTR2450. Lifestyle changes in women with polycystic ovary syndrome (PCOS), overweight and a wish to have children. *PCOS and overweight. -* 2010.

Oberg E. Effects of lifestyle intervention in overweight women with polycystic ovary syndrome-aspects on reproduction, metabolism, psychological well-being and sleep. *Dissertation Abstracts International: Section B: The Sciences and Engineering* 2023: **84**; No-Specified.

Oostingh E, Steegers-Theunissen R, Koster W, Van Dijk M, Willemsen S, Broekmans F, Hoek A, Goddijn M, Klijn N, Van Santbrink E*, et al.* The coaching program 'Smarter Pregnancy' is the first effective m Health intervention to adopt healthy nutrition and lifestyle behaviours in subfertile couples: a randomised controlled trial. *Human Reproduction* 2019: **34**.

Oostingh EC, Koster MPH, van Dijk MR, Willemsen SP, Broekmans FJM, Hoek A, Goddijn M, Klijn NF, van Santbrink EJP, Steegers EAP*, et al.* First effective mHealth nutrition and lifestyle

coaching program for subfertile couples undergoing in vitro fertilization treatment: a single-blinded multicenter randomized controlled trial. *Fertility and Sterility* 2020: **114(5)**; 945-954.

Oostingh EC, Koster MPH, Van Dijk MR, Willemsen SP, Steegers EAP, Laven JSE, and Steegers-Theunissen RPM. Improvement of periconception nutrition and lifestyle behaviors using the mhealth program smarter pregnancy: A randomized controlled trial. *Reproductive Sciences* 2018: **25**; 197A.

Osman MM, Mullins E, Kleprlikova H, Wilkinson IB, and Lees C. Beetroot juice, exercise, and cardiovascular function in women planning to conceive. *Journal of Hypertension* 2024: **42**; 101-108.

Overby NC, Medin AC, Valen EL, Salvesen L, Wills AK, Engeset D, Vik FN, and Hillesund ER. Effectiveness of a digital dietary intervention program targeting young adults before parenthood: protocol for the PREPARED randomised controlled trial. *BMJ Open* 2021: **11**; e055116.

PACTR201711002709233. Effect of aerobic exercise on ovulatory function and quality of life among women with anovulatory infertility. 2017.

PACTR202206710963782. Effect of structured Educational Sessions Regarding Lifestyle Modification on Health-Related Quality of Life among Women with Polycystic Ovary Syndrome: randomized controlled trial. 2022.

Palomba S, Falbo A, Giallauria F, Russo T, Rocca M, Tolino A, Zullo F, and Orio F. Six weeks of structured exercise training and hypocaloric diet increases the probability of ovulation after clomiphene citrate in overweight and obese patients with polycystic ovary syndrome: a randomized controlled trial. *Human reproduction (Oxford, England)* 2010: **25**; 2783-2791.

Palomba S, Giallauria F, Falbo A, Russo T, Grieco A, Colao A, Lombardi G, and Orio F. Structured exercise training plus hypocaloric diet improves ovarian sensitivity to clomiphene citrate in polycystic ovary syndrome (PCOS) patients. *Endocrine Abstracts* 2010: **22**; P476.

Palomba S, Giallauria F, Falbo A, Russo T, Oppedisano R, Tolino A, Colao A, Vigorito C, Zullo F, and Orio F. Structured exercise training programme versus hypocaloric hyperproteic diet in obese polycystic ovary syndrome patients with anovulatory infertility: a 24-week pilot study. *Human reproduction (Oxford, England)* 2008: **23**; 642-650.

Paratmanitya Y, Helmyati S, Nurdiati DS, and Hadi H. Effect of maternal mentoring program on improving iron and folic acid intake among Indonesian pregnant women: cluster randomized trial. *Annals of Nutrition and Metabolism* 2023: **79**; 354

EP-355.

Parsons J, Forde R, Brackenridge A, Hunt KF, Ismail K, Murrells T, Reid A, Rogers H, Rogers R, and Forbes A. The gestational diabetes future diabetes prevention study (GODDESS): A partially randomised feasibility controlled trial. *PloS one* 2022: **17**; e0273992.

Patten RK, McIlvenna LC, Levinger I, Garnham AP, Shorakae S, Parker AG, McAinch AJ, Rodgers RJ, Hiam D, Moreno-Asso A*, et al.* High-intensity training elicits greater improvements in cardio-metabolic and reproductive outcomes than moderate-intensity training in women with polycystic ovary syndrome: A randomized clinical trial. *Human Reproduction* 2022: **37**; 1018-1029.

Pedro J, Fernandes J, Barros A, Xavier P, Oliveira C, Schmidt L, Costa ME, and Martins MV. Does watching an educational video increase Fertility Awareness (FA)? Results from a randomised controlled trial with partnered people desiring to become parents. *Human Reproduction* 2019: **34**.

Pfuller B, Ibragimova E, Klaua S, Machlitt A, Rohde W, and Voigt K. Effect of metformin treatment combined with lifestyle modification on reproductive features, endocrine and metabolic profiles in obese infertile women with PCOS. *The 20th annual meeting of the european society of human reproduction and embryology* 2004; i176.

Phelan S, Jelalian E, Coustan D, Caughey AB, Castorino K, Hagobian T, Muñoz-Christian K, Schaffner A, Shields L, Heaney C*, et al.* Randomized controlled trial of prepregnancy lifestyle intervention to reduce recurrence of gestational diabetes mellitus. *American Journal of Obstetrics and Gynecology* 2023: **229**; 158.e151-158.e114.

Potdar RD, Sahariah SA, Gandhi M, Kehoe SH, Brown N, Sane H, Dayama M, Jha S, Lawande A, Coakley PJ*, et al.* Improving women's diet quality preconceptionally and during gestation: effects on birth weight and prevalence of low birth weight--a randomized controlled efficacy trial in India (Mumbai Maternal Nutrition Project). *The American journal of clinical nutrition* 2014: **100**; 1257-1268.
Price S, Nankervis A, Permezel M, Prendergast L, Sumithran P, and Proietto J. Health consequences for mother and baby of substantial pre-conception weight loss in obese women: study protocol for a randomized controlled trial. *Trials* 2018: **19**; 248.
Price S, Sumithran P, Nankervis A, Permezel M, Craig J, Prendergast L, and Proietto J. Pilot Study: The impact of substantial pre-conception weight loss in obese women on glucose control at 26–28 weeks of pregnancy. *Obesity research & clinical practice* 2019: **13**; 102-103.
Price SA, Sumithran P, Prendergast LA, Nankervis AJ, Permezel M, and Proietto J. Time to pregnancy after a prepregnancy very-low-energy diet program in women with obesity: substudy of a randomized controlled trial. *Fertility and sterility* 2020: **114**; 1256-1262.
Price SAL, Sumithran P, Nankervis AJ, Permezel M, Prendergast LA, and Proietto J. Impact of preconception weight loss on fasting glucose and pregnancy outcomes in women with obesity: A randomized trial. *Obesity (Silver Spring, Md.)* 2021: **29**; 1445-1457.
Raab R, Hoffmann J, Spies M, Geyer K, Meyer D, Gunther J, and Hauner H. Are pre- and early pregnancy lifestyle factors associated with the risk of preterm birth? A secondary cohort analysis of the cluster-randomised GeliS trial. *BMC pregnancy and childbirth* 2022: **22**; 230.
Radin R, Mumford SL, Silver RM, Lynch AM, Perkins N, Sjaarda L, and Schisterman E. Recent weight-control efforts before trying to conceive, fecundability, and ovulation among fecund women. *Fertility and sterility* 2016: **106**; e102.
RBR-7by76r. Effects of " Anti-stress Therapy" on behavior and stress hormones in infertile women and also on weight loss after diet and exercise guidance in those overweight. *Effects of " Mind Body Therapy" on behavioral and neuroendocrine markers of stress in infertile women and also on reducing the body mass index after diet and exercise guidance in those with overweight and obesity* 2016.
RBR-10ftp9pm. Effect of dietary changes for weight loss and fertility parameters in overweight and obese women. *Effect of nutritional intervention for weight loss and fertility indicators in Overweight and Obesity women* 2024.
Rochester JR, Kwiatkowski CF, Lathrop MK, Neveux I, Daza EJ, Grzymski J, and Hua J. Reducing Exposures to Endocrine Disruptors (REED) study, a personalized at-home intervention program to reduce exposure to endocrine disrupting chemicals among a child-bearing age cohort: study protocol for a randomized controlled trial. *Trials* 2024: **25**; 793.
Rono K, Stach-Lempinen B, Eriksson JG, Poyhonen-Alho M, Klemetti MM, Roine RP, Huvinen E, Andersson S, Laivuori H, Valkama A*, et al.* Prevention of gestational diabetes with a prepregnancy lifestyle intervention - findings from a randomized controlled trial. *International journal of women's health* 2018: **10**; 493-501.
Rothberg A, Lanham M, Randolph J, Fowler C, Miller N, and Smith Y. Feasibility of a brief, intensive weight loss intervention to improve reproductive outcomes in obese, subfertile women: a pilot study. *Fertility and sterility* 2016: **106**; 1212-1220.
Rouissi M, Jean-Denis F, Belan M, and Baillargeon JP. A PRECONCEPTION LIFESTYLE INTERVENTION IMPROVES SOME GESTATIONAL OUTCOMES AND NEONATAL MARKERS OF ADIPOSITY IN WOMEN WITH OBESITY AND INFERTILITY. *Fertility and sterility* 2020: **114**; e466-e467.
Rouissi M, Levesque MA, Hebert MC, Jean-Denis F, Belan M, Langlois MF, Ainmelk Y, Carranza-Mamane B, Pesant MH, and Baillargeon JP. A Preconception Lifestyle Intervention Maintained Throughout Pregnancy Improves Some Gestational and Neonatal Outcomes in Women With Obesity and Infertility. *Journal of the Endocrine Society* 2020: **4**; A1134.
Sadeghi M, Farajkhoda T, Khanabadi M, and Eftekhar M. PERMA model vs. integrative-behavioral couple therapy for fertility problems: A randomized clinical trial protocol. *International journal of reproductive biomedicine* 2021: **19**; 1105-1116.
Sahariah SA, Gandhi M, Chopra H, Kehoe SH, Johnson MJ, di Gravio C, Patkar D, Sane H, Coakley PJ, Karkera AH*, et al.* Body Composition and Cardiometabolic Risk Markers in Children of

Women who Took Part in a Randomized Controlled Trial of a Preconceptional Nutritional Intervention in Mumbai, India. *The Journal of nutrition* 2022: **152**; 1070-1081.
Salama AA, Amine EK, Salem HAE, and Abd El Fattah NK. Anti-Inflammatory Dietary Combo in Overweight and Obese Women with Polycystic Ovary Syndrome. *North American journal of medical sciences* 2015: **7**; 310-316.
Sant'Anna EM, Paiva SPC, Santos RP, Rodrigues AM, Nery SF, Maia FP, Ferreira AVM, and Reis F. Mindfulness-based intervention for lifestyle modification and weight loss in infertile women: Randomized controlled trial. *Human Reproduction* 2017: **32**.
Sant'Anna EM, Paiva SPC, Santos RP, Rodrigues AMS, Davis NA, Nery SF, Maia FP, Ferreira AVM, and Reis FM. Mindfulness-based program to support lifestyle modification and weight loss in infertile women: randomized controlled trial. *Journal of psychosomatic obstetrics and gynaecology* 2020; 1-9.
Sauder KA, Gamalski K, DeRoeck J, Vasquez FP, Dabelea D, Glueck DH, Catenacci VA, Fabbri S, and Ritchie ND. A pre-conception clinical trial to reduce intergenerational obesity and diabetes risks: the NDPP-NextGen trial protocol. *Contemporary clinical trials* 2023: **133**; 107305.
Shabani M, Omidi S, Farmanbar R, and Hamzegardeshi Z. Effect of preconception counseling on health promoting behaviors of reproductive age women in Sari city. *Journal of Nursing & Midwifery Sciences* 2016: **3**; 1-10.
Shapiro M, Kaing A, Christ JP, and Huddleston HG. PHYSICAL ACTIVITY DURING FERTILITY CARE (PACE): a RANDOMIZED CONTROLLED TRIAL OF EXERCISE DURING OVARIAN STIMULATION. *Fertility and sterility* 2024: **122**; e119.
Sim KA, Dezarnaulds GM, Denyer GS, Skilton MR, and Caterson ID. Weight loss improves reproductive outcomes in obese women undergoing fertility treatment: a randomized controlled trial. *Clinical Obesity* 2014: **4**; 61-68.
Soepnel LM, Draper CE, Mabetha K, Dennis C-L, Prioreschi A, Lye S, and Norris SA. A protocol for monitoring fidelity of a preconception-life course intervention in a middle-income setting: the Healthy Life Trajectories Initiative (HeLTI), South Africa. *Trials* 2022: **23**; 758.
Sordia-Hernandez LH, Rodriguez PA, Rodriguez DS, Guzman ST, Zenteno ESS, Gonzalez GG, and Patino RI. Effect of a low glycemic diet in patients with polycystic ovary syndrome and anovulation -A randomized controlled trial. *Clinical and Experimental Obstetrics and Gynecology* 2016: **43**; 555-559.
Steegers-Theunissen RPM. Preconceptional personalised mHealth lifestyle coaching:\rFirst results of a randomized controlled trial in couples undergoing IVF/ICSI treatment. *Human Reproduction* 2018: **33**.
Sujan MAJ, Skarstad HMS, Rosvold G, Fougner SL, Nyrnes SA, Iversen AC, Follestad T, Salvesen KA, and Moholdt T. Randomised controlled trial of preconception lifestyle intervention on maternal and offspring health in people with increased risk of gestational diabetes: Study protocol for the before the BEGINNING trial. *BMJ Open* 2023: **13**; :e073572.
Sujan MAJ, Skarstad HS, Rosvold G, Fougner SL, Nyrnes SA, Iversen AC, Follestad T, Salvesen KA, and Moholdt T. A randomised controlled trial of preconception lifestyle intervention on maternal and offspring health in people with increased risk of gestational diabetes: study protocol for the BEFORE THE BEGINNING trial. *medRxiv* 2023.
Sujan MAJ, Skarstad HS, Rosvold G, Fougner SL, Nyrnes SA, Iversen AC, Follestad T, Salvesen KA, and Moholdt T. Effect of a preconception lifestyle intervention on cardiometabolic outcomes in females at increased risk for gestational diabetes: a randomised controlled trial. *European journal of preventive cardiology* 2024: **31**; I63.
Sun L and Niu Z. A mushroom diet reduced the risk of pregnancy-induced hypertension and macrosomia: a randomized clinical trial. *Food & nutrition research* 2020: **64**.
Svensson H, Einarsson S, Olausson D, Kluge L, Bergh C, Eden S, Lonn M, and Thurin-Kjellberg A. Inflammatory and metabolic markers in relation to outcome of in vitro fertilization in a cohort of predominantly overweight and obese women. *Scientific reports* 2022: **12**; 13331.
Syndrome EodtwawCitoPO. Effect of diet therapy with and without Curcumin in treatment of Polycystic Ovary Syndrome. *Investigation of the Effects of DASH and Standard Diets With and Without Curcumin Supplementation on Gene Expression of Interleukin-1 Alpha , 5 Alpha*

– Reductase, Serum Concentration of Testosterone and Glycemic Indices in Infertile Women With Polyc 2020.

Szigeti JF, Kazinczi C, Szabo G, Sipos M, Ujma PP, and Purebl G. The clinical effectiveness of the Mind/Body Program for Infertility on wellbeing and assisted reproduction outcomes: a randomized controlled trial in search for active ingredients. *Human Reproduction* 2024: **39**; 1735

EP-1751.

Talluto C. The effects of a six-week aerobic and weight-resistance training program on infertility patients diagnosed with polycystic ovary syndrome. *Fertility and sterility* 2002: **78**; S152.

Taneja S, Chowdhury R, Dhabhai N, Mazumder S, Upadhyay RP, Sharma S, Dewan R, Mittal P, Chellani H, Bahl R*, et al.* Impact of an integrated nutrition, health, water sanitation and hygiene, psychosocial care and support intervention package delivered during the pre- and peri-conception period and/or during pregnancy and early childhood on linear growth of infants in the first two years of life, birth outcomes and nutritional status of mothers: study protocol of a factorial, individually randomized controlled trial in India. *Trials* 2020: **21**; 127.

Taneja S, Chowdhury R, Dhabhai N, Upadhyay RP, Mazumder S, Sharma S, Bhatia K, Chellani H, Dewan R, Mittal P*, et al.* Impact of a package of health, nutrition, psychosocial support, and WaSH interventions delivered during preconception, pregnancy, and early childhood periods on birth outcomes and on linear growth at 24 months of age: factorial, individually randomised controlled trial. *BMJ (Clinical research ed.)* 2022: **379**; e072046.

Tate D, Miller A, Ramser K, Harris A, Price J, Moses-Simmons L, and Gomez L. Group prenatal care model use in pre-gestational diabetes. *American journal of obstetrics and gynecology* 2018: **218**; S59.

Tavousi SA, Zanjani Z, Mohammadi N, and Omidi A. The effects of mindfulness-based stress reduction on psychological symptoms, quality of life, and marital satisfaction in infertile women undergoing IVF: A randomized clinical trial. *Nursing & Midwifery Studies* 2024: **13**; 33-39.

Thibodeau A, Lafleche CRD, Jean-Denis F, Harnois-Leblanc S, Perron P, Mathieu MARIEEVE, Dallaire F, Morisset AS, Brochu M, and Baillargeon JP. Follow-up of Children Born in a Randomized Controlled Trial Evaluating a Preconception Lifestyle Intervention in Women Living With Obesity and Infertility. *Canadian journal of diabetes* 2024: **48**; S22.

Timmermans YEG, van de Kant KDG, Reijnders D, Kleijkers LMP, Dompeling E, Kramer BW, Zimmermann LJI, Steegers-Theunissen RPM, Spaanderman MEA, and Vreugdenhil ACE. Towards Prepared mums (TOP-mums) for a healthy start, a lifestyle intervention for women with overweight and a child wish: study protocol for a randomised controlled trial in the Netherlands. *BMJ Open* 2019: **9**; e030236.

Tolahunase M, Kumar R, Sagar R, and Dada R. Impact of yoga-and meditation-based lifestyle intervention on depression and quality of life in infertile couples: A randomized controlled trial. *Human Reproduction* 2018: **33**.

Tolahunase MR, Sagar R, Chaurasia P, and Dada R. IMPACT OF YOGA- AND MEDITATION-BASED LIFESTYLE INTERVENTION ON DEPRESSION, QUALITY OF LIFE, AND CELLULAR AGING IN INFERTILE COUPLES. *Fertility and sterility* 2018: **110**; e67.

Turner-McGrievy GM, Davidson CR, Wingard EE, and Billings DL. Low glycemic index vegan or low-calorie weight loss diets for women with polycystic ovary syndrome: a randomized controlled feasibility study. *Nutrition research (New York, N.Y.)* 2014: **34**; 552-558.

Upadhyay RP, Taneja S, Chowdhury R, Dhabhai N, Sapra S, Mazumder S, Sharma S, Tomlinson M, Dua T, Chellani H*, et al.* Child Neurodevelopment After Multidomain Interventions From Preconception Through Early Childhood: the WINGS Randomized Clinical Trial. *JAMA* 2024: **331**; 28.

van der Windt M, Schoenmakers S, Willemsen S, van Rossem L, and Steegers-Theunissen R. Optimizing the Periconception Lifestyle of Women With Overweight Using a Blended Personalized Care Intervention Combining eHealth and Face-to-face Counseling (eFUSE): Protocol for a Randomized Controlled Trial. *JMIR research protocols* 2021: **10**; e28600.

van Dijk MR, Koster MPH, Oostingh EC, Willemsen SP, Steegers EAP, and Steegers-Theunissen RPM. A Mobile App Lifestyle Intervention to Improve Healthy Nutrition in Women Before and During Early Pregnancy: Single-Center Randomized Controlled Trial. *Journal of medical Internet research* 2020: **22**; e15773.

van Elten TM, Karsten MDA, Geelen A, van Oers AM, van Poppel MNM, Groen H, Gemke RJBJ, Mol BW, Mutsaerts MAQ, Roseboom TJ*, et al.* Effects of a preconception lifestyle intervention in obese infertile women on diet and physical activity; A secondary analysis of a randomized controlled trial. *PloS one* 2018: **13**; e0206888.

van Elten TM, van de Beek C, Geelen A, Gemke R, Groen H, Hoek A, Mol BW, van Poppel MNM, and Roseboom TJ. Preconception Lifestyle and Cardiovascular Health in the Offspring of Overweight and Obese Women. *Nutrients* 2019: **11**.

Van Elten TM, Van Poppel MNM, Gemke RJBJ, Groen H, Hoek A, Mol BW, and Roseboom TJ. Cardiometabolic Health in Relation to Lifestyle and Body Weight Changes 3-8 Years Earlier. *Nutrients* 2018: **10**.

van Oers AM, Mutsaerts MAQ, Burggraaff JM, Kuchenbecker WKH, Perquin DAM, Koks CAM, van Golde R, Kaaijk EM, Broekmans FJ, de Bruin JP*, et al.* Association between periconceptional weight loss and maternal and neonatal outcomes in obese infertile women. *PloS one* 2018: **13**; e0192670.

Van Oers AM, Mutsaerts MAQ, Burggraaff JM, Kuchenbecker WKH, Perquin DAM, Koks CAM, Van Golde R, Kaaijk EM, Schierbeek JM, Oosterhuis GJE*, et al.* Cost-effectiveness of a structured lifestyle program in overweight and obese subfertile women. Preliminary data from a randomised controlled trial-the LIFEstyle study. *Human Reproduction* 2014: **29**.

Van Uytsel H, Bijlholt M, Devlieger R, Ameye L, Jochems L, van Holsbeke C, Schreurs A, Catry V, and Bogaerts A. Effect of the e-health supported INTER-ACT lifestyle intervention on postpartum weight retention and body composition, and associations with lifestyle behavior: A randomized controlled trial. *Prev Med* 2022: **164**; 107321.

Wang X, Cai S, Tang S, Yang L, Tan J, Sun X, and Gong F. Effect of lifestyle or metformin interventions before IVF/ICSI treatment on infertile women with overweight/obese and insulin resistance: a factorial design randomised controlled pilot trial. *Pilot and Feasibility Studies* 2023: **9**.

Wang Z, Groen H, Cantineau AEP, van Elten TM, Karsten MDA, van Oers AM, Mol BWJ, Roseboom TJ, and Hoek A. Dietary Intake, Eating Behavior, Physical Activity, and Quality of Life in Infertile Women with PCOS and Obesity Compared with Non-PCOS Obese Controls. *Nutrients* 2021: **13**.

Wang Z, Groen H, Cantineau AEP, van Elten TM, Karsten MDA, van Oers AM, Mol BWJ, Roseboom TJ, and Hoek A. Effectiveness of a 6-Month Lifestyle Intervention on Diet, Physical Activity, Quality of Life, and Markers of Cardiometabolic Health in Women with PCOS and Obesity and Non-PCOS Obese Controls: One Size Fits All? *Nutrients* 2021: **13**.

Wang Z, Groen H, Va Zomeren KC, Cantineau AEP, Va Oers A, Va Montfoort APA, Kuchenbecker WKH, Pelinck MJ, Broekmans FJ, Klijn NF*, et al.* Lifestyle intervention prior to IVF does not improve embryo utilization rate and cumulative live birth rate in women with obesity. *Human Reproduction* 2021: **36**.

Williams NI, Lieberman J, and De Souza MJ. Increase in perceived stress distinguishes severity of menstrual disruption in young women in response to a 3 month diet and exercise intervention. *FASEB Journal* 2016: **30**.

Young MF, Nguyen P, Tran LM, Khuong LQ, Tandon S, Martorell R, and Ramakrishnan U. Maternal hemoglobin concentrations across pregnancy and child health and development from birth through 6-7 years. *Frontiers in nutrition* 2023: **10**; 1114101.

Zhang J, Si Q, and Li J. Therapeutic effects of metformin and clomiphene in combination with lifestyle intervention on infertility in women with obese polycystic ovary syndrome. *Pakistan journal of medical sciences* 2017: **33**; 8-12.

제남주 and 최소영. 예비부부 대상 웹 기반 수태 전 건강증진 프로그램 개발 및 평가. *Journal of Korean Academy of Nursing* 2016: **46**; 720-732.
